# New Dual Inhibitors of Bacterial Topoisomerases with Broad-Spectrum Antibacterial Activity and In Vivo Efficacy against Vancomycin-Intermediate *Staphylococcus aureus*

**DOI:** 10.1021/acs.jmedchem.2c01905

**Published:** 2023-03-06

**Authors:** Martina Durcik, Andrej Emanuel Cotman, Žan Toplak, Štefan Možina, Žiga Skok, Petra Eva Szili, Márton Czikkely, Elvin Maharramov, Thu Hien Vu, Maria Vittoria Piras, Nace Zidar, Janez Ilaš, Anamarija Zega, Jurij Trontelj, Luis A. Pardo, Diarmaid Hughes, Douglas Huseby, Tália Berruga-Fernández, Sha Cao, Ivailo Simoff, Richard Svensson, Sergiy V. Korol, Zhe Jin, Francisca Vicente, Maria C. Ramos, Julia E. A. Mundy, Anthony Maxwell, Clare E. M. Stevenson, David M. Lawson, Björn Glinghammar, Eva Sjöström, Martin Bohlin, Joanna Oreskär, Sofie Alvér, Guido V. Janssen, Geert Jan Sterk, Danijel Kikelj, Csaba Pal, Tihomir Tomašič, Lucija Peterlin Mašič

**Affiliations:** † Faculty of Pharmacy, University of Ljubljana, Aškerčeva cesta 7, Ljubljana 1000, Slovenia; ‡ Synthetic and Systems Biology Unit, Institute of Biochemistry, Biological Research Centre, Szeged H-6726, Hungary; § Max Planck Institute for Multidisciplinary Sciences, Oncophysiology, Hermann-Rein-Str. 3, Göttingen 37075, Germany; ∥ Department of Medical Biochemistry and Microbiology, Uppsala University, Husargatan 3, Uppsala 75123, Sweden; ⊥ Drug Optimization and Pharmaceutical Profiling Platform (UDOPP) Department of Pharmacy, Uppsala University, Husargatan 3, Uppsala 75123, Sweden; # Department of Medical Cell Biology, Uppsala University, Husargatan 3, Uppsala 75123, Sweden; ¶ Fundación Medina, Avenida del Conocimiento 34, Parque Tecnológico Ciencias de la Salud, Granada 18016, Spain; ∇ Department of Biochemistry and Metabolism, John Innes Centre, Norwich Research Park, Norwich NR4 7UH, U.K.; ○ Department of Chemical and Pharmaceutical Toxicology, RISE Research Institutes of Sweden, Södertälje 15136, Sweden; ⧫ Department of Chemical Processes and Pharmaceutical Development, RISE Research Institutes of Sweden, Södertälje 15136, Sweden; †† Medicinal Chemistry Division, Vrije Universiteit Amsterdam, De Boelelaan 1108, Amsterdam 1081 HZ, The Netherlands

## Abstract

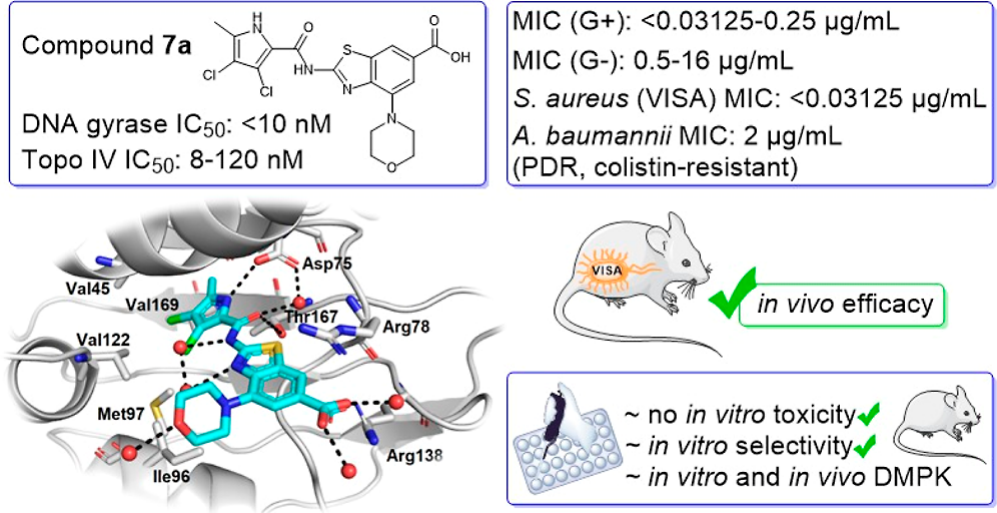

A new series of dual low nanomolar benzothiazole inhibitors of bacterial DNA gyrase and topoisomerase IV were developed. The resulting compounds show excellent broad-spectrum antibacterial activities against Gram-positive *Enterococcus faecalis*, *Enterococcus faecium* and multidrug resistant (MDR) *Staphylococcus aureus* strains [best compound minimal inhibitory concentrations (MICs): range, <0.03125–0.25 μg/mL] and against the Gram-negatives *Acinetobacter baumannii* and *Klebsiella pneumoniae* (best compound MICs: range, 1–4 μg/mL). Lead compound **7a** was identified with favorable solubility and plasma protein binding, good metabolic stability, selectivity for bacterial topoisomerases, and no toxicity issues. The crystal structure of **7a** in complex with *Pseudomonas aeruginosa* GyrB24 revealed its binding mode at the ATP-binding site. Expanded profiling of **7a** and **7h** showed potent antibacterial activity against over 100 MDR and non-MDR strains of *A. baumannii* and several other Gram-positive and Gram-negative strains. Ultimately, in vivo efficacy of **7a** in a mouse model of vancomycin-intermediate *S. aureus* thigh infection was also demonstrated.

## Introduction

Due to the rapid emergence of drug-resistant bacteria, the post-antibiotic era has essentially begun, with fewer drugs being available for the successful treatment of many bacterial infections.^1,2^ Thus, in the 21st century, antimicrobial resistance (AMR) represents a major public health issue, and the World Health Organization (WHO) has listed AMR as one of the 10 biggest threats to global health.^3^ It is predicted that by 2050, AMR will cause at least 10 million deaths per year unless we successfully tackle this problem.^4^ In a recent review, it was estimated that 1.27 million deaths were directly attributable to antibacterial drug resistance in 2019 worldwide.^5^ The six leading pathogens contributing to the burden of AMR in 2019 were Gram-negative *Escherichia coli*, *Klebsiella pneumoniae*, *Acinetobacter baumannii*, and *Pseudomonas aeruginosa* and Gram-positive *Staphylococcus aureus* and *Streptococcus pneumoniae*.^5^ All have been included in the WHO’s 2017 priority list of pathogens for which new antibiotics are urgently needed and are also highlighted in CDC 2019 Antibiotic Resistance Threats Report.^6,7^ Methicillin-resistant *S. aureus* (MRSA) was identified as the leading pathogen responsible for the most deaths related to antibiotic resistance in 2019.^5^ MRSA mainly causes skin, soft tissue, bone and bloodstream infections, and it is the most common cause of postoperative wound infections. In many parts of the world, including Europe and the USA, the levels of community-acquired MRSA infections also tend to increase rapidly.^8−11^

**Figure 1:**
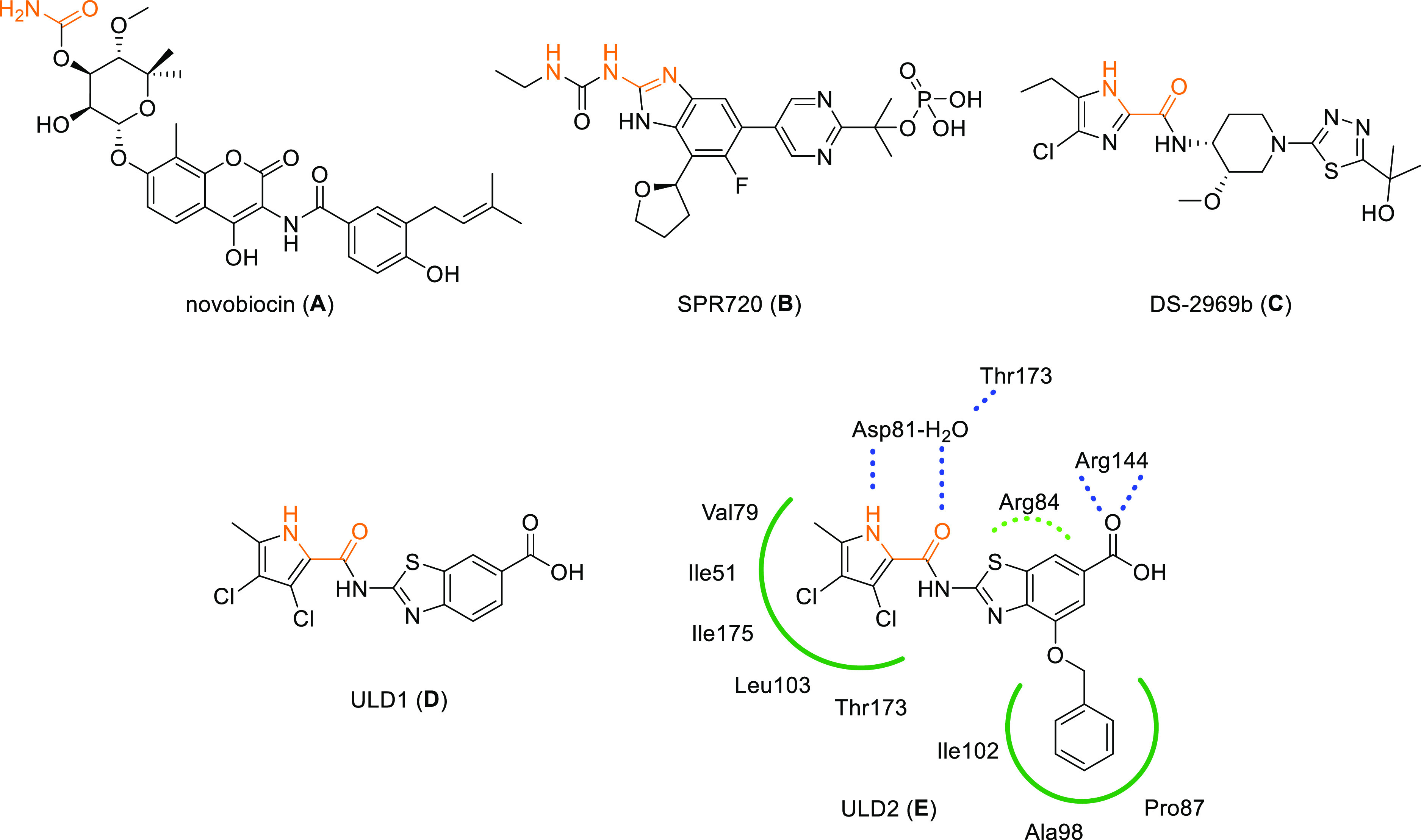
Structures of representative GyrB/ParE inhibitors (A–E). Key parts of the structures that interact with aspartate and the conserved water molecule are shown in orange. Schematic representation of the interactions observed in the crystal structure (PDB code 6TCK)^28^ is shown for inhibitor **E** at the binding site of *S. aureus* GyrB. Hydrogen bonds are presented as blue dashed lines, cation−π interactions as a green dashed curve, and hydrophobic interactions as green solid curves.

Validated targets for the development of new antibacterial agents include bacterial enzymes DNA gyrase and topoisomerase IV (topo IV), which belong to type IIA topoisomerases.^12^ Regarding current antibiotics in clinical use, these are the targets of fluoroquinolones, which are definitely among the most effective antibacterials utilized in clinical practice.^13^ However, even this class of antibiotics faces the challenges of side effects and emerging bacterial resistance.^14−16^ Topoisomerases play an important role in DNA topology related to processes like DNA replication, transcription, repair, and decatenation.^13,17^ During DNA replication, DNA gyrase removes the positive supercoils ahead of the replication fork, while topo IV unlinks replicated daughter chromosomes.^13,18^ Both enzymes are heterotetramers, composed of two ATP-binding subunits, GyrB or ParE in DNA gyrase or topo IV, respectively, and two GyrA or ParC subunits that bind to the DNA. Due to their homologous structures, these enzymes offer the possibility of dual-targeting, which may prevent or prolong the onset of target-based resistance.^12,17^ Fluoroquinolones inhibit GyrA by stabilizing the DNA–enzyme cleavage complex, leading to its conversion into a lethal lesion, that is, a double-stranded DNA break.^19^ On the other hand, GyrB or ParE inhibitors block the ATPase function of the enzymes, depriving the bacterial cell of the topoisomerase activity required for the replication process.^20^ Extensive research has been performed to date on ATP-competitive inhibitors; however, only one compound, novobiocin (Figure 1A), was approved for clinical use, and even this one was withdrawn from therapy in 2011 due to safety concerns and resistance development.^21^ Two other compounds are currently being investigated in clinical trials, namely, fobrepodacin or SPR720 (Figure 1B), for the treatment of nontuberculous mycobacterial infections^22^ and DS-2969b (Figure 1C) for the treatment of *Clostridium difficile* infection.^23^

We have developed and optimized several structural classes of ATP-competitive inhibitors, including *N*-phenylpyrrolamides, tetrahydrobenzothiazoles, and benzothiazoles.^24−31^ Recently, we have developed two novel, potent and balanced dual inhibitors [Figure 1D (ULD1) and 1E (ULD2)] of DNA gyrase (GyrB) and topo IV (ParE) with a benzothiazole core, evaluated their bioactivities, and studied their potential for resistance development.^28^ Moreover, based on these two compounds, we have designed and synthesized a series of new dual-targeting inhibitors that, like **D** and **E**, possess potent antibacterial activities against the problematic ESKAPE (*Enterococcus faecium*, *S. aureus*, *K. pneumoniae*, *A. baumannii*, *P. aeruginosa*, and *Enterobacter* species) pathogens.^28,31^ This group of resistant Gram-negative and Gram-positive bacteria is known to be responsible for the majority of nosocomial infections and is highly resistant to clinically available antibiotics.^32,33^ Most of these microbes are also included in the priority lists of WHO and CDC.^6,7^

With compound **E**, we entered into the IMI ENABLE (European Gram-negative antibacterial engine) hit-to-lead project to optimize its physicochemical properties while retaining the low nanomolar dual inhibition of GyrB and ParE and broad-spectrum antibacterial activity.^34^ In the present study, we present a new series of benzothiazole-based dual inhibitors with potent antibacterial activity. For the most promising dual GyrB and ParE inhibitor **7a**, derived from **E**, we performed in-depth preclinical studies for microbiological evaluation, in vitro safety, DMPK, and selectivity and have demonstrated that it displays favorable properties. Ultimately, we have demonstrated its in vivo efficacy in a neutropenic mouse thigh infection model infected with a vancomycin-intermediate *S. aureus* (VISA) strain.

## Results and Discussion

### Structure-Based Design

**Figure 2:**
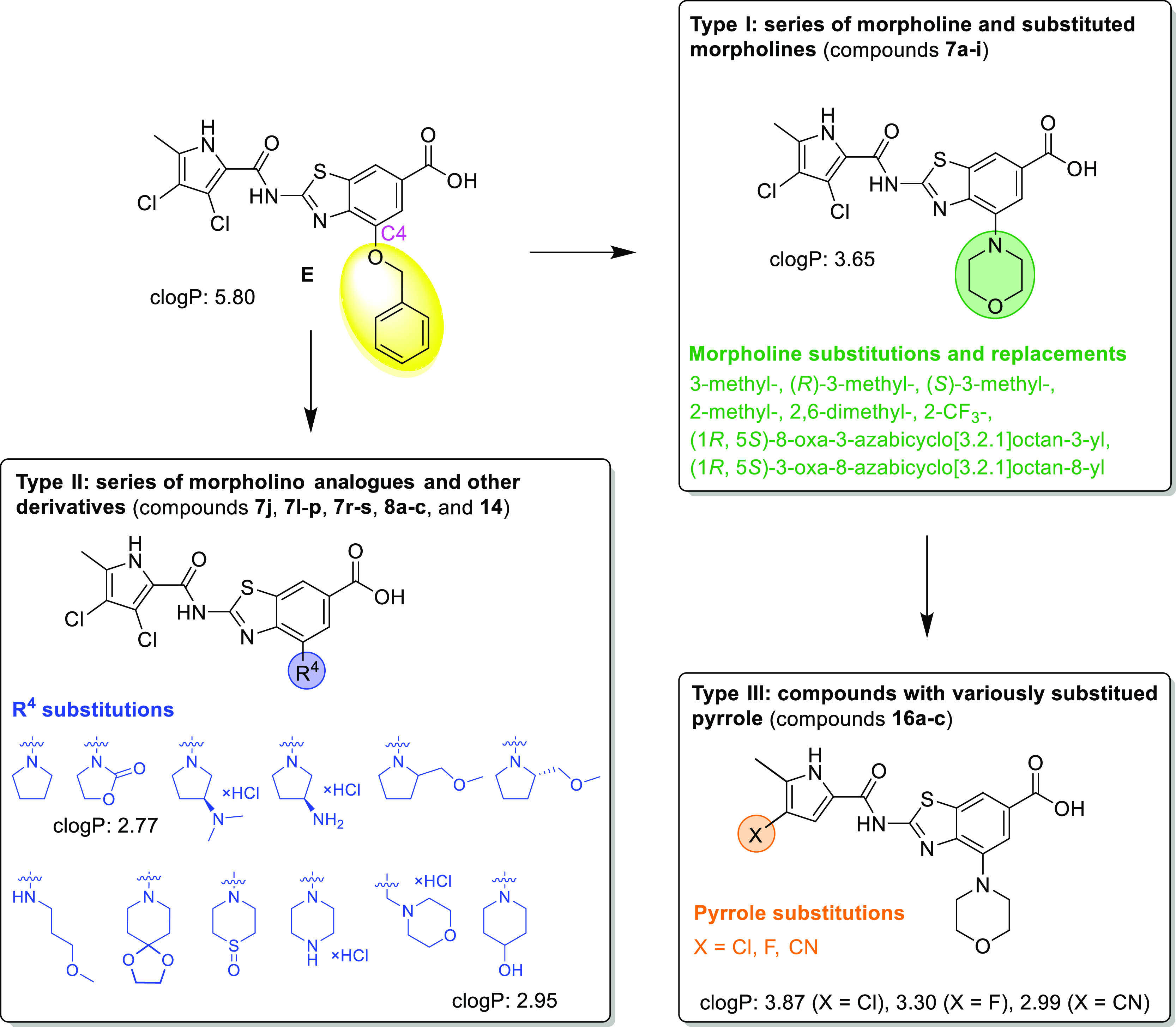
Design of type I–III compounds based on inhibitor **E**. clog*P* values were calculated using ChemDraw Professional 20.0.

Agents **D** and **E**, as well as our recently developed series of novel compounds showed promising results in terms of balanced low nanomolar dual enzyme inhibition and potent antibacterial activity. Moreover, inhibitors **D** and **E** seem to have negligible potential for resistance acquisition.^28,31^ However, these compounds have disadvantages, such as low solubility (**E**: 6.6 μM) and high plasma protein binding (PPB) (**E**: >99%). Therefore, the aim of the present study was the hit-to-lead optimization of this class of dual GyrB/ParE inhibitors, as well as to define a lead compound with favorable properties and potent activity, manifested in in vivo efficacy against systemic infections in mice. The design of new benzothiazole-based analogues is presented in Figure 2.

Based on compound **E**, we first synthesized type I and type II inhibitors with the pyrrolo-benzothiazole core left intact since it forms crucial interactions at the binding site, as depicted in Figure 1. As seen in the crystal structure of **E** forming a complex with *S. aureus* GyrB, the pyrrolamide part forms a hydrogen-bond network with Asp81 and the conserved water molecule, the terminal carboxylate forms a salt bridge with Arg144, and the benzothiazole core forms cation−π interactions with Arg84. The pyrrole ring also forms several hydrophobic interactions at the binding pocket. We focused on the substituent at position C4 of the benzothiazole core, which is also important for additional interactions with the GyrB subunit of the enzyme. We designed novel inhibitors by replacing the benzyloxy group with polar aliphatic substituents to increase the polarity and reduce the aromaticity of the compound, and thus to reduce clog*P* and improve solubility. We used morpholine and substituted morpholines to prepare type I compounds (Figure 2). Morpholine is a privileged fragment in many drugs; its oxygen lone pairs can act as hydrogen-bond acceptors and can form additional interactions at the binding site. Type II compounds have basic substituents such as aminopyrrolidines and morpholino analogues, such as oxidothiomorpholine or piperidine and pyrrolidine derivatives attached to the central core of the molecule (Figure 2). Because type I compounds were later shown to be superior to type II, we also synthesized a set of analogues (type III), in which morpholine was retained at the C4 position of the benzothiazole ring, and 3,4-dichloro-5-methyl-1*H*-pyrrole was replaced by 4-chloro-5-methyl-, 4-fluoro-5-methyl- or 4-cyano-5-methyl-1*H*-pyrrole, again with the aim to reduce log*P* and improve the physicochemical properties of the compounds (type III, Figure 2).

### Chemistry

**Scheme 1:**
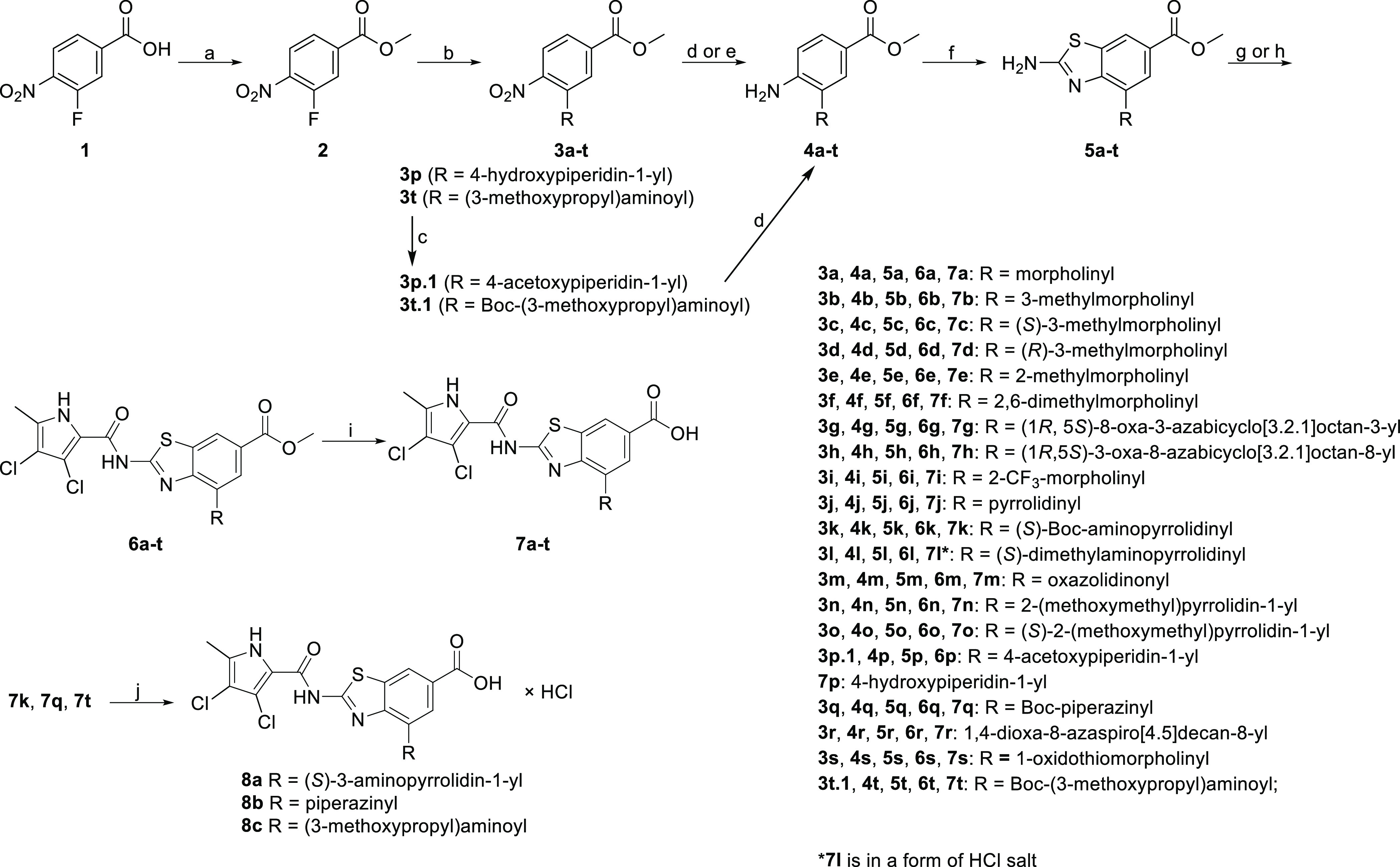
Reagents and conditions: (a) MeOH, H_2_SO_4_, 65 °C, 15 h; (b) corresponding amine, K_2_CO_3_ or DIPEA (for **3g**, **3n–o**, and **3r**), CH_3_CN or *N*,*N*-dimethylformamide (DMF), 40–60 °C or rt (for **3g**, **3p**, and **3r**), 15 h; (c) acetic anhydride, pyridine, acetonitrile, 70 °C, 72 h (for **3p.1**) or di-*tert*-butyl dicarbonate, DMAP, tetrahydrofuran (THF), 40 °C, 15 h (for **3t.1**); (d) H_2_, Pd/C, MeOH, rt, 3 h (for **4a–f**, **4i–4r**, and **4t**); (e) Fe, acetic acid, rt, 15 h (for **4g**, **4h**, and **4s**); (f) KSCN, Br_2_, CH_3_COOH, 10 °C, then 20 °C, 15 h, 25% NH_3_ aq solution or 2 M NaOH (for **5p** and **5s**); (g) (i): 3,4-dichloro-5-methyl-1*H*-pyrrol-2-carboxylic acid, (COCl)_2_, anhydrous dichloromethane (DCM), 20 °C, 15 h, (ii): corresponding amine, toluene, 130 °C, 15 h (for **6a–k**, **6m–p**, **6r**, and **6t**); (h) 2,2,2-trichloro-1-(3,4-dichloro-5-methyl-1*H*-pyrrol-2-yl)ethan-1-one (for **6l**, **6q**, and **6s**), corresponding amine, Na_2_CO_3_, DMF, 60–70 °C, 15 h; (i) 1–2 M NaOH, MeOH or 1,4-dioxane (for **7s**), 40 °C, 24–96 h; and (j) 4 M HCl in 1,4-dioxane, 1,4-dioxane, rt, 7–48 h.

Scheme 1 presents the synthesis of *N*-substituted compounds **7a–j**, **7l–p**, **7r**, **7s**, and **8a–c** of types I and II. 3-Fluoro-4-nitrobenzoic acid (**1**) was converted to **2** with the Fisher esterification method. Compound **2** was then substituted with various amines under basic conditions to get **3a–t**. The hydroxyl group of **3p** and the amino group of **3t** were protected with acetyl and *tert*-butyloxycarbonyl protecting groups, respectively, to get compounds **3p.1** and **3t.1**. Derivatized nitro compounds **3a–o**, **3q–s**, **3p.1**, and **3t.1** were reduced to amino derivatives **4a–t** using catalytic hydrogenation or iron-mediated reduction. Amines were used to synthesize benzothiazoles **5a–t** using bromine and KSCN in acetic acid according to our general protocol, with modifications to the reagents’ equivalents when necessary.^35^ Corresponding benzothiazoles were coupled to either 3,4-dichloro-5-methyl-1*H*-pyrrol-2-carboxylic acid (to get **6a–k**, **6m–p**, **6r**, and **6t**) in a two-step reaction or to 2,2,2-trichloro-1-(3,4-dichloro-5-methyl-1*H*-pyrrol-2-yl)ethan-1-one (to get **6l**, **6q**, and **6s**). Esters **6a–t** were hydrolyzed with 1 or 2 M NaOH to acids **7a–t**. In the final step, the Boc-protecting group was removed from compounds **7k**, **7q**, and **7t** with acidolysis to produce final compounds **8a–c**.

**Scheme 2:**
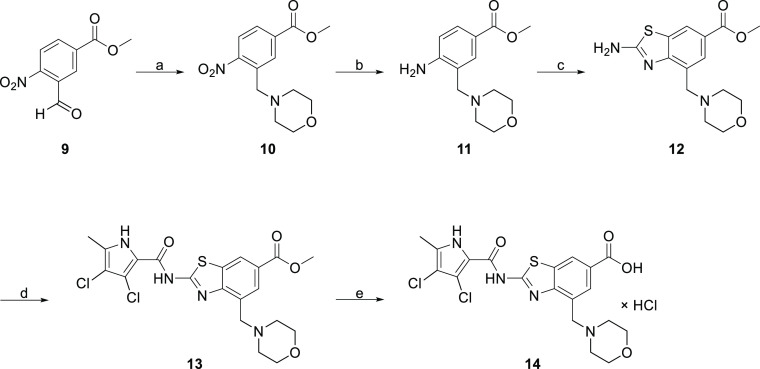
Reagents and conditions: (a) morpholine, NaCNBH_3_, CH_3_COOH, rt, 15 h; (b) H_2_, Pd/C, MeOH, rt, 3 h; (c) KSCN, Br_2_, CH_3_COOH, 10 °C, then 20 °C, 15 h, 4 M NaOH, (d) 2,2,2-trichloro-1-(3,4-dichloro-5-methyl-1*H*-pyrrol-2-yl)ethan-1-one, Na_2_CO_3_, 60 °C, 15 h; and (f) 2 M NaOH, MeOH, 40 °C, 48 h.

Scheme 2 shows the synthesis of type II inhibitor **14**. Morpholine was first attached to compound **9** via reductive amination. The nitro group of the obtained compound **10** was reduced by catalytic hydrogenation to give amine **11**. Next, cyclization was performed to obtain benzothiazole **12** which was coupled with 2,2,2-trichloro-1-(3,4-dichloro-5-methyl-1*H*-pyrrol-2-yl)ethan-1-one. In the final step, ester **13** was hydrolyzed with 2 M NaOH to carboxylic acid **14**.

**Scheme 3:**
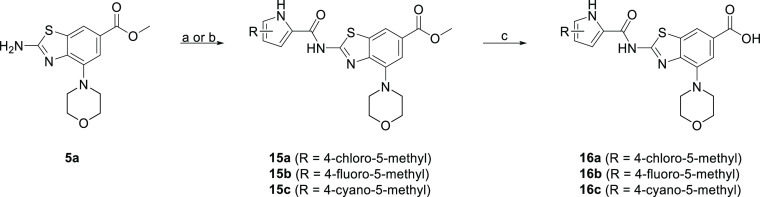
Reagents and conditions: (a) 2,2,2-trichloro-1-(4-chloro-5-methyl-1*H*-pyrrol-2-yl)ethan-1-one^36^ (for **15a**), **5a**, Na_2_CO_3_, DMF, 60–70 °C, 15 h; (b) (i): 4-fluoro-5-methyl-1*H*-pyrrol-2-carboxylic acid^36^ (for **15b**) or 4-cyano-5-methyl-1*H*-pyrrol-2-carboxylic acid (for **15c**), (COCl)_2_, anhydrous DCM, 20 °C, 15 h, (ii): **5a**, toluene, 130 °C, 15 h; and (c) 1 M NaOH (for **16a–b**) or 2 M LiOH (for **16c**), MeOH, 40 °C, 48–96 h.

Inhibitors **16a–c** of type III are synthesized according to Scheme 3. Compound **5a** was coupled with 2,2,2-trichloro-1-(4-chloro-5-methyl-1*H*-pyrrol-2-yl)ethan-1-one using Na_2_CO_3_ in DMF to get **15a** or to 4-fluoro-5-methyl-1*H*-pyrrol-2-carbonyl chloride to get **15b**. The pyrrole building blocks were prepared according to previously published procedures.^36^ Compound **15c** was synthesized by coupling **5a** with 4-cyano-5-methyl-1*H*-pyrrol-2-carbonyl chloride. The obtained esters were then hydrolyzed to their carboxylic acid analogues **16a–c**.

### Enzyme Inhibition and Antibacterial Activity

All compounds were tested for their inhibitory activities against DNA gyrase and topo IV from *E. coli* in supercoiling and relaxation high-throughput plate assays, respectively. Our new series of inhibitors were then tested against a panel of Gram-positive and Gram-negative bacteria. Results [IC_50_ and minimum inhibitory concentration (MIC) values] for compounds of type I are presented in Table 1, and results for compounds of types II and III are shown in Tables S1 and S2 of the Supporting Information supplement. All new inhibitors were found to show low nanomolar inhibition of DNA gyrase from *E. coli* with IC_50_ values <32 nM. Also, type I compounds exhibited low nanomolar IC_50_ values (<100 nM) against Topo IV, indicating a strong dual activity of these novel inhibitors (Table 1). Greater differences were observed for types II and III against Topo IV from *E. coli*, with **7j**, **7s**, **8a**, and **16a** having IC_50_ values of <100 nM similar to type I agents, while **7l**, **8b**, and **14** from type II having secondary or tertiary amine substituents, and **7m** having oxazolidinone attached to C4 inhibited topo IV with IC_50_ values ranging from 120 to 460 nM (Supporting Information, Table S1). Nevertheless, all synthesized inhibitors were found to act as dual-targeting compounds. Type III compounds **16b** and **16c** with a fluoro or cyano group on the pyrrole ring showed IC_50_ values of 120 and 440 nM against *E. coli* topo IV, respectively (Supporting Information, Table S2).

**Table 1:**
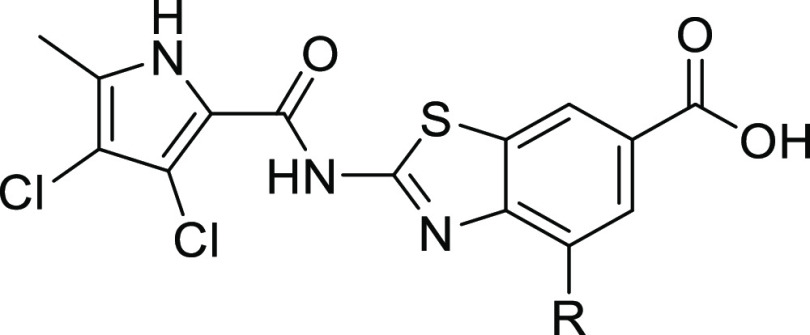

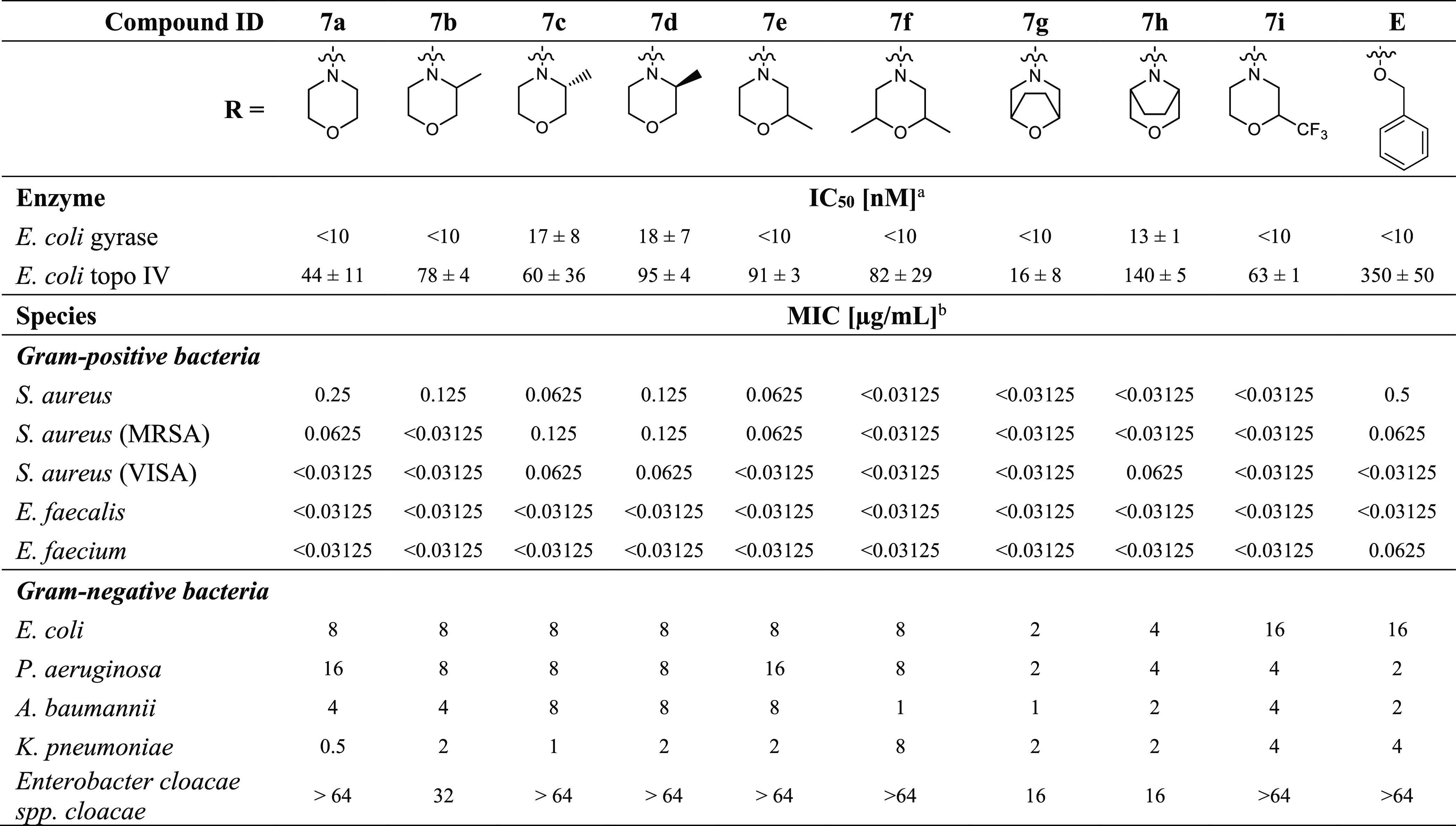
Inhibitory Activities of Type I Compounds (**7a–i**) against DNA Gyrase and Topo IV from *E. coli* in Supercoiling and Relaxation HTS Assays, Respectively, and Their Antibacterial Activities

a IC_50_, concentration (mean ± SD of three independent experiments) that inhibits enzyme activity by 50%.

b MIC, minimum inhibitory concentration. Bacterial strains used for MIC determination: *S. aureus* ATCC 29213, *S. aureus* (MRSA) ATCC 43300, *S. aureus* (VISA) ATCC 700699, *Enterococcus faecalis* ATCC 29212, *E. faecium* ATCC 700221, *E. coli* ATCC 25922, *P. aeruginosa* ATCC 27853, *A. baumannii* ATCC 17978, *K. pneumoniae* ATCC 10031, and *Enterobacter cloacae* spp. *E. cloacae* ATCC 13047. MIC measurements were performed according to the Clinical and Laboratory Standards Institute guidelines, with three independent measurements.

Type I compounds with substituted morpholines were found to have the most balanced dual inhibition of GyrB and ParE, which was translated into potent antibacterial activity. All compounds from this group demonstrated excellent and comparable MIC values against Gram-positive pathogens *S. aureus*, MRSA, VISA, *Enterococcus faecalis*, and *E. faecium*. All MIC values were below 0.25 μg/mL, with the majority being <0.03125 μg/mL (Table 1). Moreover, potent activities were detected against Gram-negative bacteria. Specifically, compounds **7g** and **7h** with bridged morpholines showed the highest antibacterial activities, with MIC values in the range of 1–4 μg/mL against *E. coli*, *P. aeruginosa*, *A. baumannii*, and *K. pneumoniae* and 16 μg/mL against *Enterobacter cloacae*. Of note, these were the only compounds of this series to be active against the bacterial strain *E. cloacae*. Other type I inhibitors displayed comparably good inhibition of Gram-negative strains, with most MIC values being in the range of 1–8 μg/mL (Table 1).

Type II inhibitors were also demonstrated to exhibit dual inhibitory activity against both enzymes; however, this activity was less balanced compared to type I compounds. For type II inhibitors, topo IV inhibition was slightly weaker, with IC_50_ values ranging from 38 to 460 nM. Overall, compounds **7j**, **7r**, and **8c** with pyrrolidine, 1,4-dioxa-8-azaspiro[4.5]decane, and methoxypropylamino substituents showed the highest antibacterial activity in this class of analogues, with good MIC values, ranging from 2 to 16 μg/mL against the Gram-negatives *K. pneumoniae*, *A. baumannii*, *P. aeruginosa*, and *E. coli* (Supporting Information, Table S1). Again, their inhibitory activity against Gram-positive bacteria was more pronounced, characterized by MIC values between <0.03125 and 0.25 μg/mL. Introducing a free hydroxyl group (**7p**) and primary (**8a**), secondary (**8b**), or tertiary (**7l** and **14**) aliphatic amines into the molecule resulted in the loss of antibacterial activity against Gram-negative strains (except for **7p** and **8a** against *K. pneumoniae* with MICs of 4 μg/mL) and also weakened the activities of these derivatives against Gram-positives. Oxazolidinone and oxidothiomorpholine as C4 substituents (compounds **7m** and **7s**) did not improve antibacterial activity despite acceptable enzyme inhibition by these agents. Only **7m** possessed notable activity against VISA, *E. faecalis,* and *E. faecium* (MIC values: 8, 4, 4 μg/mL, respectively; Supporting Information, Table S1).

Modifying the substituents on the pyrrole ring in type III compounds did not yield favorable results as the inhibition of topo IV deteriorated for fluoromethyl pyrrole **16b** and cyanomethyl pyrrole **16c**. These two compounds were devoid of Gram-negative antibacterial activity, and **16b** showed only weak activity against Gram-positive *E. faecium*, VISA, and *E. faecalis* (MIC values: 2, 8, and 16 μg/mL, respectively; Supporting Information, Table S2). Compound **16a** with chloromethyl pyrrole was the best inhibitor of this type, with an IC_50_ value of 38 nM against topo IV and thus a balanced inhibition of both enzymes. This compound also retained antibacterial activity against Gram-positive strains and against the Gram-negative strain *K. pneumoniae* (MIC = 2 μg/mL; Supporting Information, Table S2).

### Physicochemical Properties

Despite its potent activity and in vivo efficacy in a mouse model of dermal infection, compound **E**, our starting point for the optimization and design of new analogues, has some drawbacks that limit its potential for systemic use. These obstacles include low solubility (6.6 μM), high aromaticity and lipophilicity (clog*P*: 5.80), and loss of antibacterial activity in the presence of serum. The present study was designed to overcome these issues, so we assessed the thermodynamic solubility of representatives of all three compound types (Supporting Information, Table S3) and PPB of type I inhibitors (Supporting Information, Table S4) as this type of compounds showed the highest bioactivities.

As predicted in our design, replacing the benzyloxy fragment of compound **E** with morpholine (**7a**) resulted in better thermodynamic solubility of 98 μM (168 μM in a second method) (Supporting Information, Table S3). However, incorporating lipophilic substituents onto the morpholine ring reduced solubility. While the addition of a simple methyl group (**7b** and **7e**) resulted in a slightly lower but comparable solubility of 68 and 80 μM, respectively, the substitution of morpholine with a lipophilic CF_3_ group (**7i**) reduced solubility to 11 μM (Supporting Information, Table S3). The least soluble inhibitors were compounds with dimethylmorpholino (**7f**) and bridged morpholines (**7g** and **7h**), characterized by solubility values in the range of 0.26–3.8 μM. Therefore, increasing the sp^3^ character of the molecule did not lead to improved solubility. On the other hand, **7p** with a 4-hydroxypiperidine attached to the central molecule was the most soluble compound of the series (184 μM, Table S3), but unfortunately it lost antibacterial activity against Gram-negatives. Using **7a** as a model compound and replacing the 3,4-dichloro-5-methyl-1*H*-pyrrole with 4-chloro-5-methyl-1*H*-pyrrole or 4-fluoro-5-methyl-1*H*-pyrrole yielded inhibitors with better solubility: **16a** (137 μM, Table S3) and **16b** (131 μM, Table S3). However, these pyrroles were not studied further as these compounds lost the potent antibacterial activity of the parent compound. Similarly, measuring PPB revealed that substitutions on the morpholine ring, even as small as a methyl group, negatively impacted the amount of the unbound fraction in the presence of mouse serum (Supporting Information, Table S4). The highest percent of unbound fraction was displayed by the morpholino compound **7a** (fu = 1.8%), and then, the percentage dropped from **7a** to methylmorpholino compounds (**7b–d**, fu: 1.1–1.6%), then to bridged morpholino inhibitors (**7g** and **7h**, fu = 0.4 and 0.9%, respectively), and finally to trifluoromethylmorpholine (**7i**) which was completely bound to mouse proteins (fu < 0.1%) (Supporting Information, Table S4).

Compound **7a** showed balanced dual enzyme inhibition of DNA gyrase and topo IV from *E. coli*, potent antibacterial activity, and good solubility. Although some type I compounds demonstrated superior inhibition of Gram-negative bacteria, **7a** has the advantages of showing the highest solubility and the lowest level of PPB in mouse serum, thus having the highest amount of the free fraction (1.8%). Its log*D* value was determined to be 1.83 (pH = 7.4), which is favorable for improved oral bioavailability. Thus, **7a** was defined as the lead of the series, and its potential was further explored with several assays.

### Expanded Enzyme Inhibition Profile of **7a**

In addition to DNA gyrase and topo IV from *E. coli*, **7a** was tested for its inhibitory activity against enzymes from *S. aureus*, *A. baumannii*, and *P. aeruginosa* (Table 2, Supporting Information, Figures S1 and S2). **7a** inhibited DNA gyrase from all strains with a potent IC_50_ of <10 nM. Low nanomolar inhibitory activities were obtained against topo IV from all strains as well; however, the activity against both topoisomerases from *A. baumannii* was the least balanced with a 50-fold weaker inhibition of topo IV. On the other hand, **7a** showed perfectly balanced and potent dual inhibition of DNA gyrase and topo IV from *S. aureus* (IC_50_ values of 1.2 and 8.0 nM, respectively) which could contribute to its potent antibacterial activity against the Gram-positives.

**Table 2:** Inhibitory Activities of **7a** against DNA Gyrase and Topo IV from *S aureus*, *E. coli*, *A. baumannii*, and *P. aeruginosa*

		IC_50_ [nM]^a^		
enzyme		**7a**	ciprofloxacin	novobiocin
*S. aureus*	DNA gyrase^b^	1.22 ± 0.04	12 170 ± 1 245	0.65 ± 0.03
	topo IV^b^	8.0 ± 1.2	6 075 ± 1 648	13 265 ± 7
*E. coli*	DNA gyrase^c^	<10	120 ± 20	170 ± 20
	topo IV^c^	44 ± 11	5 400 ± 2 100	11 000 ± 2 000
*A. baumannii*	DNA gyrase^b^	2.42 ± 0.28	1 267 ± 344	2.17 ± 0.16
	topo IV^b^	119.7 ± 8.34	2 885 ± 332	9 465 ± 799
*P. aeruginosa*	DNA gyrase^c^	<10	nt^d^	nt
	topo IV^b^	27.5 ± 16.3	3 895 ± 63.6	8 365 ± 445

a IC_50_, concentration (mean ± SD of three independent experiments) that inhibits enzyme activity by 50%.

b Tested in gel-based supercoiling (for DNA gyrase) and decatenation (for topo IV) assays.

c Tested in supercoiling (for DNA gyrase) and relaxation (for topo IV) HTS assays.

d nt, not tested.

### Crystal Structure of **7a**

**Figure 3:**
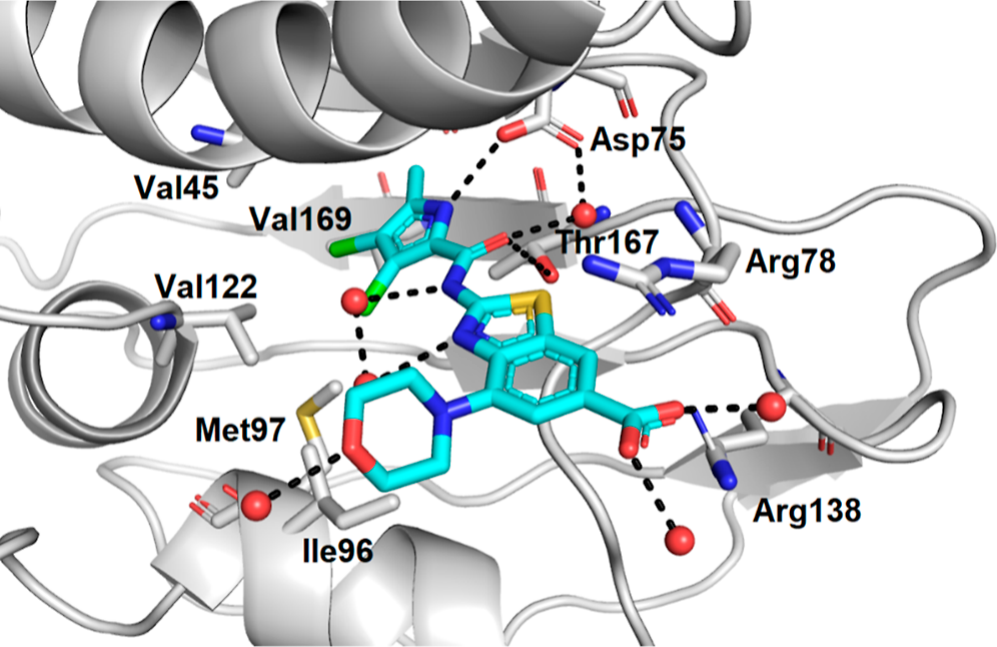
Binding mode of inhibitor **7a** (in cyan sticks) in complex with *Pseudomonas aeruginosa* GyrB24 (in gray cartoon; PDB entry: 8BN6) determined by X-ray crystallography at 1.6 Å resolution. For clarity, only amino acid residues forming hydrophobic interaction, hydrogen bonds, or cation−π interactions are shown as gray sticks. Water molecules are presented as red spheres. Hydrogen bonds are shown as black dashed lines.

The crystal structure of **7a** in complex with *P. aeruginosa* GyrB24 was obtained at a resolution of 1.6 Å (Figure 3). The binding mode of **7a** resembled the binding mode of **E** in its complex with *S. aureus* GyrB24.^28^ The pyrrolamide moiety formed a network of hydrogen bonds with Asp75, Thr167, and water molecules, as well as several hydrophobic contacts with Val45, Val73, Ile96, Met97, Val122, and Val169 in the hydrophobic pocket of the ATP-binding site of GyrB. The benzothiazole scaffold was further stabilized by a cation−π interaction with the Arg78 side chain and a network of water molecules. The strongest interaction formed was a salt bridge between the carboxylate group of **7a** and the Arg138 guanidinium side chain. The morpholine ring was oriented perpendicular to the central benzothiazole core and pointed toward the lipophilic floor of the binding site, where it formed a hydrogen bond with a water molecule.

### In-Depth Microbiological Profiling of **7a**

Balanced dual-targeting compounds are considered to be less prone to resistance as it demands simultaneous acquisition of multiple, specific mutations. Using standard protocols, we assessed the frequencies-of-resistance after exposing up to 10^12^ bacterial cells of *S. aureus* ATCC 43300 (MRSA) and *S. aureus* ATCC 700699 (VISA) to increasing concentrations of **7a** and novobiocin, a reference compound that targets the DNA gyrase subunit B only. Generally, we found that the frequency-of-resistance was lower for **7a** compared to that for novobiocin. In particular, at concentration 20 times above the wild-type MIC, the frequency-of-resistance against **7a** was exceedingly low (5 × 10^–12^, Supporting Information, Table S5 and Figure S3). On the other hand, the frequency-of-resistance against novobiocin was 9 × 10^–10^ (for MRSA) or 5 × 10^–9^ at concentration 20 times above the wild-type novobiocin MIC. When we measured the MIC of **7a** against the isolated *S. aureus* mutants, it was only 32-fold (for MRSA) and 4-fold (for VISA) higher compared to the wild-type MIC, while isolated novobiocin-resistant mutants displayed an upto 128-fold increment in novobiocin MIC (Supporting Information, Table S6). Although more detailed analyses are needed in the future, these results indicate that **7a** is not particularly prone to bacterial resistance.

**Figure 4:**
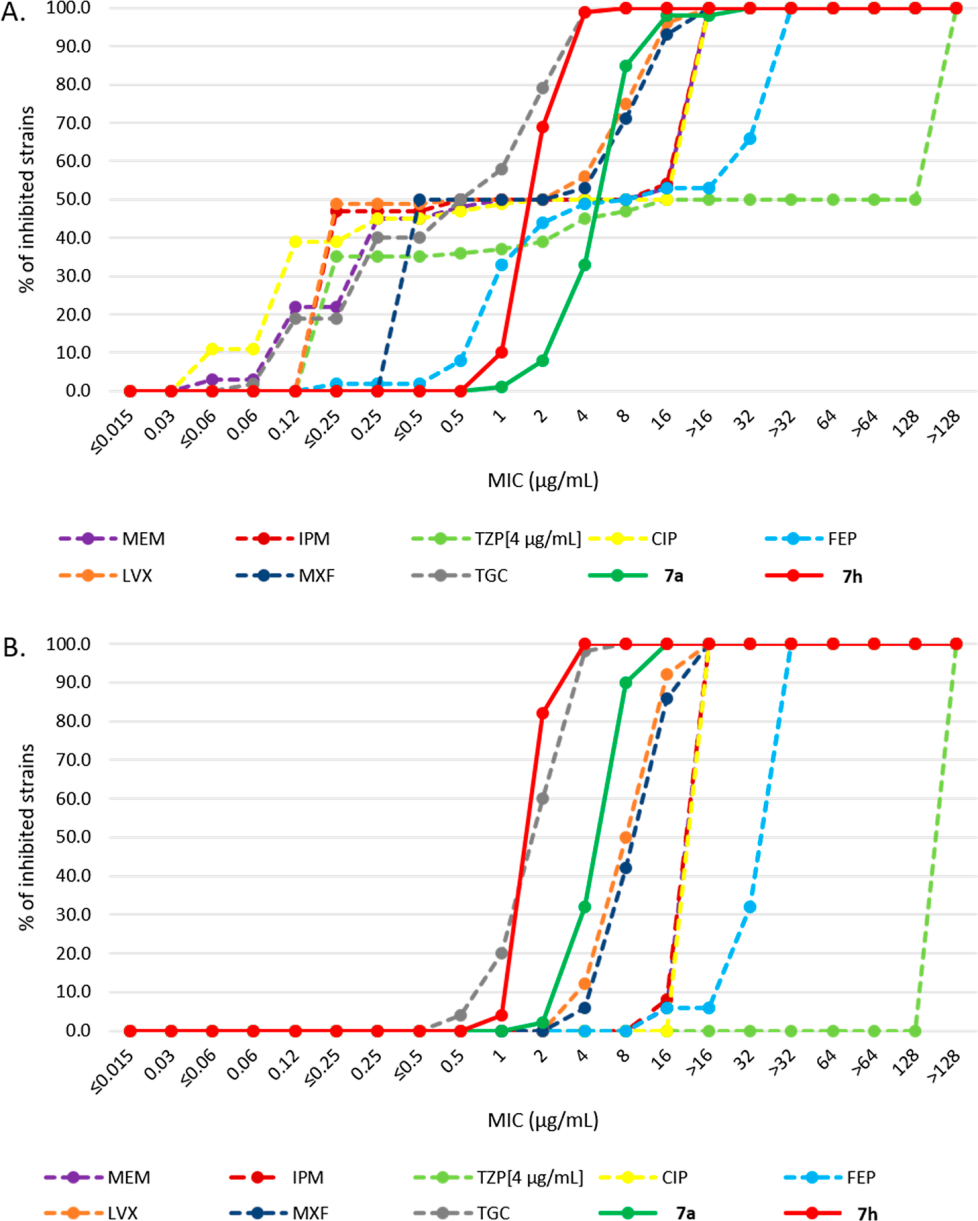
Cumulative MIC distribution against a panel of 100 *A. baumannii* strains (A) and cumulative MIC distribution against 50 MDR *A. baumannii* strains (B). Strains included 50 MDR strains and 50 non-MDR strains of diverse geographic origins. MIC values were determined at IHMA Europe via broth microdilution according to CLSI guidelines. Background data for this figure are available in the file Supporting Information_A.baumannii_MIC. Abbreviations: MEM, meropenem; IPM, imipenem; TZP, piperacillin/tazobactam; CIP, ciprofloxacin; FEP, cefepime; LVX, levofloxacin; MXF, moxifloxacin; and TGC, tigecycline.

**Figure 5:**
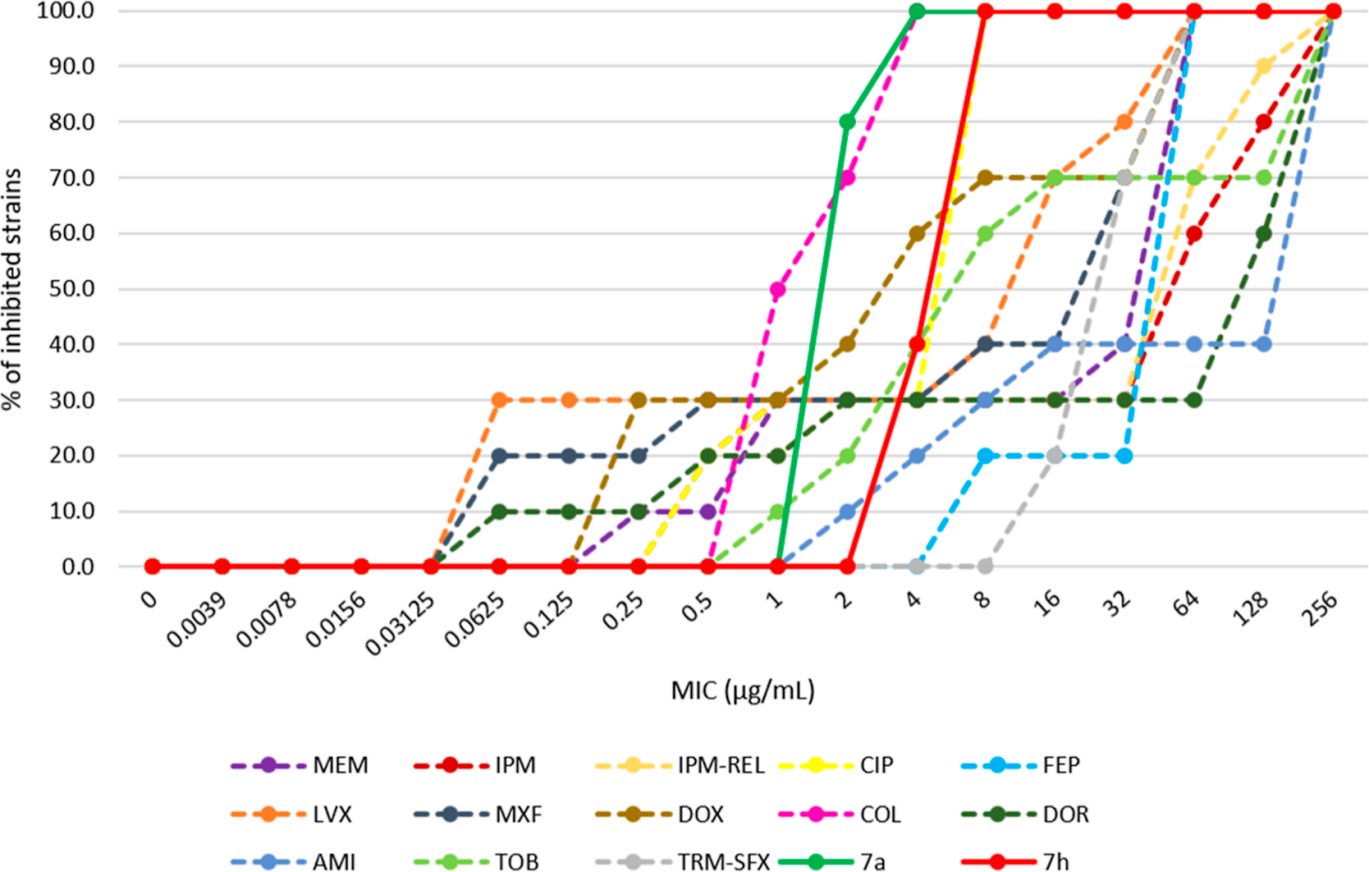
Cumulative MIC distribution against three sensitive and seven MDR *A. baumannii* clinical isolates. Abbreviations: MEM, meropenem; IPM, imipenem; IPM–REL, imipenem/relebactame (4 μg/mL); CIP, ciprofloxacin; FEP, cefepime; LVX, levofloxacin; MXF, moxifloxacin; DOX, doxycycline; COL, colistin; DOR, doripenem; AMI, amikacin; TOB, tobramycin; and TRM–SFX, trimethoprim/sulfamethoxazole (4 μg/mL).

Next, we extended the study of the antibacterial potential of **7a** and also included **7h** because it displayed slightly better antibacterial activity against Gram-negative bacteria (Table 1). To explore their potential against Gram-negatives, **7a** and **7h**, along with control antibiotics, were tested against 100 diverse *A. baumannii* strains [50% multidrug resistant (MDR) and 50% non-MDR] (Figure 4). Compound **7a** inhibited this panel of microorganisms with MIC values in the range of 1–32 μg/mL, while **7h** inhibited all strains (100%) at a concentration as low as 8 μg/mL (Figure 4A). In comparison, only one of the eight control antibiotics, tigecycline inhibited all strains at 8 μg/mL, while the others were weaker inhibitors (100% inhibition at MIC values ranging from >16 to >128 μg/mL). When focusing on MDR bacteria only, 100% inhibition of all strains was reached at 16 μg/mL by **7a** and at 4 μg/mL by **7h** (Figure 4B). Tigecycline inhibited all strains at 8 μg/mL, while the other control antibiotics showed weaker activity (>16 μg/mL). Thus, **7h** performed the best among the 10 compounds tested, and **7a** also yielded promising results. As MDR *A. baumannii* is an emerging healthcare threat in the region in recent years, we also tested **7a** and **7h** against a local subset of *A. baumannii* isolates (*n* = 10). This subset contained four recently obtained MDR clinical isolates from multiple hospital units in Hungary, with two strains showcasing a pan-resistant phenotype (they are resistant against all antibiotics that have a clinical breakpoint defined against *A. baumannii* as of the time of this publication), besides various MDR and non-MDR control strains. On this subset, both **7a** and **7h** proved very effective, with inhibition of all the tested strains at MICs of 2–4 and 4–8 μg/mL, respectively, while the other control antibiotics showed weaker activity (inhibition of 100% of the strains at 32 μg/mL) (Figure 5, Supporting Information, Figure S4, Tables S7 and S8). The activity of **7a** and **7h** was only rivaled by colistin, a last resort antibiotic that inhibited all strains at 4 μg/mL, showing the potential of these compounds against hard-to-treat infections in the region.

Inhibitor **7a** was also tested against *E. coli* ATCC 25922, *A. baumannii* BM4652, and *P. aeruginosa* PAO1, along with efflux-defective mutated strains of these bacteria and against the *E. colilps* mutant with a destabilized outer membrane (Supporting Information, Table S9). We have revealed that efflux mechanisms play an important role in the agents’ weaker activities against Gram-negative bacteria compared to the Gram-positives as the MIC values against mutated strains ranged from <0.125 to 0.25 μg/mL (>64-, >16-, and >256-fold lower compared to wild-type *E. coli*, *A. baumannii*, and *P. aeruginosa*, respectively). The activity against the *lps* mutant was improved 4-fold. Also, **7a** inhibited four additional MDR *A. baumannii* clinical isolates with MIC values of 1–4 μg/mL (Supporting Information, Table S9).

**Figure 6:**
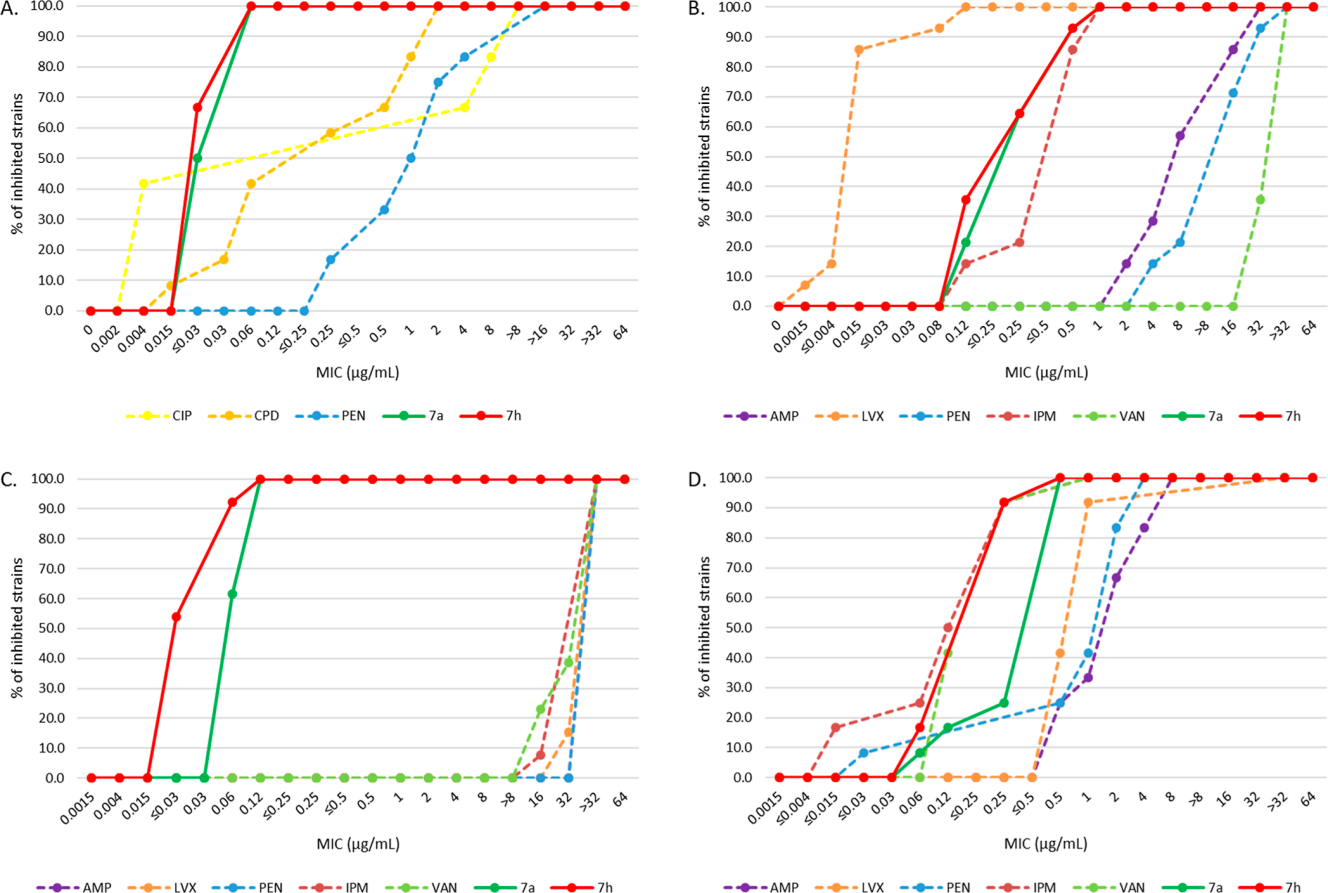
Cumulative MIC distribution against *N. gonorrhoeae* (A), *H. influenzae* (B), *E. faecium* (C), and *S. pneumoniae* strains (D). Experiments were performed at IHMA Europe. Abbreviations: CIP, ciprofloxacin; CPD, cefpodoxime; PEN, penicillin; AMP, ampicillin; LVX, levofloxacin; IPM, imipenem; and VAN, vancomycin.

Additionally, **7a** and **7h** were tested against sets of *Neisseria gonorrhoeae* (*n* = 12), *Haemophilus influenzae* (*n* = 14), *E. faecium* (*n* = 13), and *S. pneumoniae* (*n* = 12) strains and demonstrated superior efficacy compared to the control antibiotics (Figure 6, Supporting Information, Tables S10–S17). MIC values for both compounds against Gram-negative *N. gonorrhoeae* and *H. influenzae* were ≤0.06 and ≤1 μg/mL, respectively, while MIC values for both compounds against Gram-positive *E. faecium* and *S. pneumoniae* were ≤0.12 and ≤0.5 μg/mL, respectively.

### Metabolic Stability of **7a**

Compound **7a** was further examined for its metabolic stability in mouse and human liver microsomes and in mouse hepatocytes (Table 3). While microsomes predominantly contain the metabolizing enzymes belonging to the oxidative phase I metabolic system (cytochrome P450, CYP450), hepatocytes offer a more complete assessment of metabolism as they contain both phase I and phase II enzymes, such as aldehyde oxidase, monoamine oxidases, cytochromes (phase I), and UDP–glucuronyltransferases (phase II).^37,38^

**Table 3:** In Vitro Metabolic Stability of **7a**^a^

mouse hepatocytes	
in vitro *t*_1/2_ [min]	160
Cl_int_ [μL/10^6^ cells/min]	9.0
Cl_int_ (pred. in vivo) [mL/min]	2.1
Cl_H_ (pred.) [mL/min]	0.05
*E*_pred_	0.03

mouse liver microsomes	
in vitro *t*_1/2_ [min]	87
Cl_int_ [μL/10^6^ cells/min]	16
Cl_int_ (pred. in vivo) [mL/min]	1.3
Cl_H_ (pred.) [mL/min]	0.03
*E*_pred_	0.01

human liver microsomes	
in vitro *t*_1/2_ [min]	103
Cl_int_ [μL/10^6^ cells/min]	13
Cl_int_ (pred. in vivo) [mL/min]	630
Cl_H_ (pred.) [mL/min]	14
*E*_pred_	0.01

a Abbreviations: *t*_1/2_, elimination half-life; Cl_int_, intrinsic clearance; Cl_H_, hepatic clearance; *E*_pred_, predicted hepatic extraction ratio (the fraction of drug removed from blood by the liver in one passage); and pred., predicted.

In vitro assays showed that **7a** was stable in both microsomes and hepatocytes, with low predicted hepatic clearance (*E*_pred._) in human/mouse microsomes and hepatocytes (Table 3). The small difference in clearance between microsomal and hepatocyte clearances suggests primarily interaction with phase I enzymes. The compound showed minimal risk for high first-pass metabolism in both species.

### In Vitro Selectivity and Safety Studies of **7a**

To determine the selectivity of **7a** for DNA gyrase and topo IV versus human DNA TopoIIα (hTopoIIα) which has a similar ATP-binding domain to those of bacterial enzymes and belongs to the GHKL ATPase family,^39^ we evaluated the inhibitory activity of **7a** on hTopoIIα in a DNA relaxation assay. Compound **7a** had an IC_50_ of 17.0 μM against hTopoIIα (Supporting Information, Figure S5), indicating that it is over 1700-fold selective for *E. coli* DNA gyrase and over 380-fold selective for *E. coli* topo IV. Of note, most protein kinases are also enzymes with ATP-binding sites; thus, they are targeted by many synthetic inhibitors. Thus, for a thorough characterization of the selectivity of **7a**, we also tested it against 335 protein kinases from various kinase families (Supporting Information, Table S18). The selectivity scores of **7a** at 1 and 10 μM concentrations were 0.006 and 0.233, respectively, indicating and further supporting that this compound is selective for DNA gyrase and topo IV.

In vitro cytotoxicity of **7a** was evaluated in an MTS assay on a breast cancer MCF-7 cell line and a liver cancer HepG2 cell line. **7a** showed no cytotoxicity up to a concentration of 100 μM. Anyway, since the compound was found to bind to serum proteins, we performed the assay without adding fetal bovine serum to the growth media, and again no cytotoxicity was detected up to a concentration of 100 μM (Supporting Information, Figure S6). Mitochondrial toxicity was evaluated in a glu/gal assay on HepG2 cells. Inhibitor **7a** was not mitotoxic at concentrations up to 1000 μM (Supporting Information, Figure S7 and Table S19). Next, **7a** was also assayed for genetic toxicity in a standard micronucleus test on Chinese hamster ovary K1 cells with and without metabolic activation by rat liver S9 fraction (Supporting Information, Table S20). No genotoxicity of **7a** was evident up to a concentration of 50 and 100 μM with S9 activation and without metabolic activation, respectively.

To address cardiac safety, we tested **7a** for its inhibitory activity on the human ether-a-go-go-related gene (hERG) potassium ion channel (at 50 μM) and sodium Na_v_1.5 ion channel (at 10 μM), and no significant inhibition was observed. Likewise, evaluation of hemolytic activity did not raise any concerns about **7a** in this regard either (Supporting Information, Table S21).

### In Vivo Pharmacokinetic Properties of **7a**

In the in vivo pharmacokinetic profiling experiments in mice, following IV administration at 1 mg/kg, **7a** displayed moderate plasma clearance (54.8 mL/min/kg; 61% of liver blood flow; assuming a mouse liver blood flow of 90 mL/min/kg) (Table 4). The in vivo clearance kinetics were more rapid than suggested from the in vitro metabolic studies mentioned above (Table 3). Plausible reasons for this are that other elimination pathways take precedence, for example, active renal or bile clearance, and/or that the in vitro–in vivo scaling of metabolic data is poor for this compound. Also, **7a** showed a moderate volume of distribution (2.3 L/kg) and a half-life of 1.0 h.

**Table 4:** Pharmacokinetic Profiling of **7a** in Mice Following Intravenous (IV) Administration^a^^,^^b^

route	dose [mg/kg]	*t*_1/2_ [h]	*C*_0_ [ng/mL]	AUC_0–24h_ [h*ng/mL]	AUC_0–∞_ [h*ng/mL]	Cl [mL/min/kg]	*V*_ss_ [L/kg]
IV	1	1.0	913	303	304	54.8	2.3

a Male CD-1 mice (Charles River Laboratories, USA), IV, *n* = 6.

b Abbreviations: *t*_1/2_, elimination half-life, *C*_0_, initial concentration at time zero; AUC, area under the concentration–time curve; Cl, clearance; and *V*_ss_, volume of distribution at steady state.

### Formulation Study

To find the optimal formulation which would be used for determination of in vivo efficacy of inhibitor **7a**, a formulation study was performed (Supporting Information, Table S22). The highest solubility of 13.2 mg/mL was detected in 100 mM carbonate buffer (pH 9.0) with 20% hydroxypropyl β-cyclodextrin. For the actual in vivo assay, a lower pH was used, and the compound was prepared in a formulation of 100 mM carbonate buffer at pH 8.4 with 20% hydroxypropyl β-cyclodextrin.

### In Vivo Efficacy of **7a**

**Figure 7:**
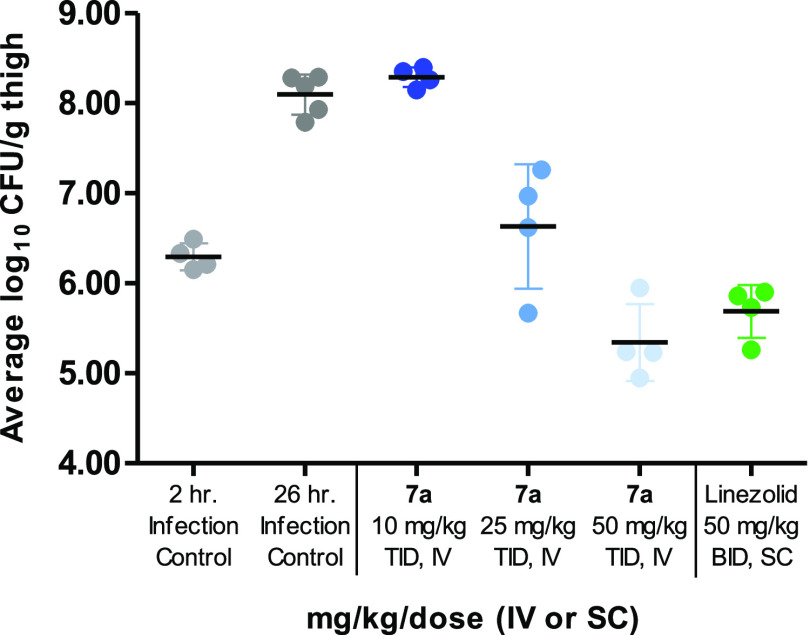
In vivo efficacy of **7a** in a neutropenic mouse thigh infection model infected with *S. aureus* ATCC 700699 (VISA). Figure shows log units of cfu levels in response to treatment with **7a** or linezolid (as a positive control). Cfus values from thigh tissue homogenates were determined at 26 h post-infection. The cfu values from each individual measurement are plotted as colored dots, and the black line represents the average cfu for each animal group. Background data for this figure are available in the Supporting Information supplement (Tables S23 and S24). Abbreviations: cfu, colony-forming unit; VISA, vancomycin-intermediate *S. aureus*; IV, intravenous; and SC, subcutaneous.

In vivo efficacy of **7a** was evaluated in a neutropenic mouse thigh infection model infected with *S. aureus* 700699 (VISA), using intravenous administration (Figure 7). Compound **7a** demonstrated a concentration-dependent dose–response trend with reductions in colony forming units (cfus) with 1.47 and 2.76 log_10_ cfus for 25 and 50 mg/kg doses, respectively, compared to controls at 26 h post-infection. Additionally, at a dose of 50 mg/kg IV (TID), **7a** demonstrated bactericidal activity with a 0.96 log_10_ cfu reduction compared to the 2 hour infection controls. Linezolid, which is one of the main antibiotics used for the treatment of systemic methicillin and vancomycin-resistant infections,^40,41^ was used as a positive control. Mice receiving linezolid at a 50 mg/kg subcutaneous (SC) dose (BID) demonstrated a 2.41 log_10_ cfu reduction compared to the 26 hour infection controls and a 0.61 log_10_ cfu reduction from the initiation of therapy (2 hour infection controls).

## Conclusions

We developed a new and advanced series of low nanomolar dual-targeting DNA gyrase and topo IV inhibitors with a benzothiazole scaffold. A morpholino substituent on C4 of the central core yielded favorable ADME (absorption, distribution, metabolism, excretion) properties for the lead compound **7a**. The crystal structure of inhibitor **7a** in complex with *P. aeruginosa* DNA gyrase was resolved, confirming its binding mode at the ATP-binding site. Compounds with morpholine and morpho derivatives in 4-position (type I) demonstrated potent broad-spectrum antibacterial activity against resistant pathogens belonging to the ESKAPE group, including MRSA, VISA, *A. baumannii*, *K. pneumoniae*, and *P. aeruginosa*. Compounds **7a** and **7h** inhibited Gram-positive strains with MIC values in the range of <0.03125–0.25 μg/mL and also inhibited the Gram-negatives *A. baumannii* and *K. pneumoniae* with MIC values ranging from 0.5 to 4 μg/mL. Moreover, 7a was selective for bacterial DNA gyrase and topo IV over human topoisomerase Iiα and showed no cytotoxicity in MCF-7 and HepG2 cells, no in vitro genotoxicity at concentrations up to 50 μM (with S9) or 100 μM (without S9), no mitotoxicity up to 1 000 μM, and no inhibition of cardiac sodium ion channel Na_v_1.5 and hERG potassium ion channel. Besides, **7a** displayed good metabolic stability and acceptable in vivo pharmacokinetic properties and demonstrated in vivo efficacy against VISA in a neutropenic mouse thigh infection model. Compounds of the presented structural type also offer points for future chemical optimization which can result in further improved properties. Various other substituents can be introduced at the C4 position of the benzothiazole ring that may contain ionizable amine to improve activity against the Gram-negatives or other functional groups that can be used to prepare pro-drug forms of the molecule. Substitutions at the C5 position with such groups could also be explored in more detail in the future. There are also possibilities for optimization at the C6 position of the benzothiazole ring with replacements of the COOH group, for example, with hydroxyalkyl groups. For these reasons and based on its promising results, **7a** may represent a good basis for the development of new antibiotics to fight MDR bacterial strains.

## Experimental Section

### Chemistry

#### General Chemistry Information

Reagents and solvents were obtained from Acros-Organics (Geel, Belgium), Apollo Scientific Ltd. (Stockport, UK), Enamine Ltd. (Kyiv, Ukraine), Fluorochem Ltd. (Derbyshire, UK), and Sigma-Aldrich (St. Louis, MO, USA) and were not further purified. Analytical TLC was performed on silica gel Merck 60 F_254_ plates (0.25 mm). The spots on the plates were visualized with UV light and, if necessary, spray reagent ninhydrin. Column chromatography was performed on silica gel 60 (particle size, 240–400 mesh). ^1^H and ^13^C NMR spectra were recorded on a Bruker AVANCE III 400 spectrometer (Bruker Corporation, Billerica, MA, USA), at 400 and 101 MHz, respectively, or on a Bruker AVANCE II 500 spectrometer (Bruker Corporation, Billerica, MA, USA), at 500 and 126 MHz, respectively, in DMSO-*d*_6_ or CDCl_3_ solutions, with tetramethylsilane as the internal standard. High-resolution mass spectra were recorded on an Exactive Plus Orbitrap mass spectrometer (Thermo Fisher Scientific, Waltham, MA, USA), and mass spectra were obtained using an ADVION expression CMS^L^ mass spectrometer (Advion Inc., Ithaca, USA). HPLC purity analyses were performed on a 1260 Infinity II LC system (Agilent Technologies Inc., Santa Clara, CA, USA). The general method used a Waters XBridge C18 column (3.5 μm, 4.6 mm × 150 mm), with 1.5 mL/min flow rate; 10 μL sample injection volume; mobile phase A: acetonitrile; and mobile phase B: 0.1% formic acid and 1% acetonitrile in ultrapure water. Gradient: 0–1.0 min, 25% A; 1.0–6.0 min, 25–98% A; 6.0–6.5 min, 98% A; 6.5–7.5 min, 98–25% A; and 7.5–10.5 min, 25% A. Optical rotations (in DMF as the solvent) were measured at 589.3 nm on an Anton Paar MCP 150 polarimeter, results for specific rotation are expressed in ° mL dm^–1^ g^–1^, and concentrations are given in mg/100 mL. All tested compounds were more than 95% pure, as established by HPLC, unless indicated otherwise. ^1^H and ^13^C NMR spectra for representative compounds and HRMS and HPLC data for lead compound can be found in the Supporting Information.

#### Synthetic Procedures and Analytical Data

##### Methyl 3-Fluoro-4-nitrobenzoate (**2**)

To a stirred suspension of compound **1** (10 g, 0.054 mol) in methanol (20 mL), H_2_SO_4_ (10.8 mL, 0.20 mol) was added, and the resulting solution was stirred at 65 °C overnight. Methanol was evaporated under reduced pressure, and saturated NaHCO_3_ solution was added and extracted with ethyl acetate. The organic phase was dried over Na_2_SO_4_, filtered, and the solvent was removed in vacuo to obtain **2** as pale-yellow crystals. Yield: 10.5 g (97.6%); pale-yellow crystals. ^1^H NMR (400 MHz, DMSO-*d*_6_): δ 3.92 (s, 3H), 7.96 (ddd, *J* = 1.0, 1.8, 8.5 Hz, 1H), 8.03 (dd, *J* = 1.7, 11.4 Hz, 1H), 8.29 (dd, *J* = 7.4, 8.5 Hz, 1H).

#### General Procedure A: Synthesis of Compounds **3a**, **3b**, **3e**, **3f**, **3i–m**, **3q**, **3s**, and **3t** (with **3j** as an Example)

Compound **2** (1.0 g, 5 mmol) was dissolved in acetonitrile (20 mL), K_2_CO_3_ (1.38 g, 10 mmol) and pyrrolidine (493 μL, 6 mmol) were added, and the reaction mixture was stirred at 60 °C for 2 h. The solvent was evaporated, and to the residue, ethyl acetate and water were added, and the phases were separated. The organic phase was washed with 1% citric acid and brine, dried over Na_2_SO_4_, filtered, and the solvent was removed in vacuo to obtain **3j** as orange crystals.

##### Methyl 3-Morpholino-4-nitrobenzoate (**3a**)

Synthesized according to General procedure A with morpholine (274 μL, 3.13 mmol) as the reactant and stirring the reaction mixture overnight. Yield: 676 mg (96.9%); orange oil. ^1^H NMR (400 MHz, DMSO-*d*_6_): δ 2.94–3.07 (m, 4H), 3.63–3.76 (m, 4H), 3.90 (s, 3H), 7.66 (dd, *J* = 1.7, 8.4 Hz, 1H), 7.78 (d, *J* = 1.7 Hz, 1H), 7.94 (d, *J* = 8.4 Hz, 1H). MS (ESI) *m*/*z*: 226.6 ([M + H]^+^).

##### Methyl 3-(3-Methylmorpholino)-4-nitrobenzoate (**3b**)

Synthesized according to general procedure A with 3-methylmorpholine (0.572 mL, 5.42 mmol) as the reactant and stirring the reaction mixture overnight. The crude product was used in the next step without further purification.

#### General Procedure B: Synthesis of Compounds **3c**, **3d**, **3g**, **3h**, **3n–p**, and **3r** (with **3g** as an Example)

Compound **2** (0.905 g, 4.54 mmol) was dissolved in DMF (15 mL), (1*R*,5*S*)-8-oxa-3-azabicyclo[3.2.1]octane (7.0 mL, 9.05 mmol) was added, and the reaction mixture was flushed with nitrogen. DIPEA (2.37 mL, 13.63 mmol) was added, and the reaction mixture was stirred at rt overnight. The reaction mixture was poured on ice cold water, and the precipitate in the mixture was filtered off to get **3g** as an yellow solid.

##### Methyl (*R*)-3-(3-Methylmorpholino)-4-nitrobenzoate (**3c**)

Synthesized according to general procedure B with (*R*)-3-methylmorpholine hydrochloride (1.00 g, 7.27 mmol) as the reactant. 6 equiv of DIPEA was used, and the reaction mixture was stirred at 60 °C for 6 days. The reaction mixture was poured on ice cold water and a saturated solution of NaHCO_3_ (20 mL). The precipitate in the mixture was filtered off, and the crude product was purified with flash column chromatography using ethyl acetate/hexane 1:2 as the eluent to give of **3c** as an orange solid. Yield: 1.07 g (52.5%); orange solid. ^1^H NMR (400 MHz, DMSO-*d*_6_): δ 0.73 (d, *J* = 6.1 Hz, 3H), 2.52 (m, 1H, signal is overlapped with the signal for DMSO), 2.87 (ddd, *J* = 3.1, 8.3, 11.6 Hz, 1H), 2.99–3.09 (m, 1H), 3.18–3.31 (m, 2H), 3.59 (ddd, *J* = 2.8, 8.3, 11.1 Hz, 1H), 3.70–3.78 (m, 2H), 3.90 (s, 3H), 3.91 (s, 1H), 7.84 (dd, *J* = 1.7, 8.4 Hz, 1H), 7.93 (d, *J* = 8.4 Hz, 1H), 7.99 (d, *J* = 1.7 Hz, 1H). MS (ESI) *m*/*z*: 280.9 ([M + H]^+^).

##### Methyl (*S*)-3-(3-Methylmorpholino)-4-nitrobenzoate (**3d**)

Synthesized according to general procedure B with (*S*)-3-methylmorpholine hydrochloride (1.90 g, 13.81 mmol) as the reactant and stirring the reaction mixture at 65 °C for 6 days. Yield: 3.80 g (98.2%); orange solid. ^1^H NMR (400 MHz, DMSO-*d*_6_): δ 0.73 (d, *J* = 6.1 Hz, 3H), 2.55 (m, 1H, signal is overlapped with the signal for DMSO), 2.87 (ddd, *J* = 3.1, 8.5, 11.7 Hz, 1H), 3.01–3.07 (m, 1H), 3.20–3.33 (m, 3H), 3.59 (ddd, *J* = 2.7, 8.3, 11.1 Hz, 1H), 3.70–3.78 (m, 2H), 3.90 (s, 3H), 7.84 (dd, *J* = 1.7, 8.3 Hz, 1H), 7.93 (d, *J* = 8.3 Hz, 1H), 7.99 (d, *J* = 1.7 Hz, 1H). MS (ESI) *m*/*z*: 280.9 ([M + H]^+^).

##### Methyl 3-(2-Methylmorpholino)-4-nitrobenzoate (**3e**)

Synthesized according to general procedure A with 2-methylmorpholine (1.07 mL, 9.89 mmol) as the reactant and stirring the reaction mixture overnight. Yield: 2.52 g (100%); orange oil. ^1^H NMR (400 MHz, DMSO-*d*_6_): δ 1.11 (d, *J* = 6.3 Hz, 3H), 2.66 (dd, *J* = 9.9, 11.8 Hz, 1H), 2.93 (ddd, *J* = 3.1, 11.1, 12.1 Hz, 1H), 2.99–3.05 (m, 1H), 3.10 (dt, *J* = 2.3, 11.8 Hz, 1H), 3.54–3.67 (m, 2H), 3.81–3.88 (m, 1H), 3.90 (s, 3H), 7.65 (dd, *J* = 1.7, 8.4 Hz, 1H), 7.77 (d, *J* = 1.8 Hz, 1H), 7.94 (d, *J* = 8.4 Hz, 1H). MS (ESI) *m*/*z*: 281.0 ([M + H]^+^).

##### Methyl 3-(2,6-Dimethylmorpholino)-4-nitrobenzoate (**3f**)

Synthesized according to general procedure A with 2,6-dimethylmorpholine (0.816 mL, 6.63 mmol) as the reactant and stirring the reaction mixture overnight. Yield: 1.60 g (98.4%); orange oil. ^1^H NMR (400 MHz, DMSO-*d*_6_): δ 1.10 (d, *J* = 6.3 Hz, 6H), 2.57 (dd, *J* = 10.1, 12.0 Hz, 2H), 3.04–3.11 (m, 2H), 3.62–3.74 (m, 2H), 3.90 (s, 3H), 7.63 (dd, *J* = 1.8, 8.4 Hz, 1H), 7.76 (d, *J* = 1.6 Hz, 1H), 7.94 (d, *J* = 8.4 Hz, 1H). MS (ESI) *m*/*z*: 294.6 ([M + H]^+^).

##### Methyl 3-((1*R*,5*S*)-8-Oxa-3-azabicyclo[3.2.1]octan-3-yl)-4-nitrobenzoate (**3g**)

Yield: 1.09 g (81.7%); yellow solid. ^1^H NMR (500 MHz, DMSO-*d*_6_): δ 1.81 (dd, *J* = 4.4, 7.8 Hz, 2H), 1.90 (q, *J* = 5.2, 6.3 Hz, 2H), 2.83–2.92 (m, 2H), 3.08 (dd, *J* = 2.1, 11.8 Hz, 2H), 3.90 (s, 3H), 4.37 (dq, *J* = 2.2, 4.5 Hz, 2H), 7.65 (dd, *J* = 1.7, 8.3 Hz, 1H), 7.75 (d, *J* = 1.7 Hz, 1H), 7.88 (d, *J* = 8.3 Hz, 1H). MS (ESI) *m*/*z*: 293.1 ([M + H]^+^).

##### Methyl 3-((1*R*,5*S*)-3-Oxa-8-azabicyclo[3.2.1]octan-8-yl)-4-nitrobenzoate (**3h**)

Synthesized according to general procedure B with (1*R*,5*S*)-3-oxa-8-azabicyclo[3.2.1]octane (2.50 g, 16.7 mmol) as the reactant. Yield: 1.709 g (69.6%), orange solid. ^1^H NMR (400 MHz, CDCl_3_): δ 1.92–2.01 (m, 2H), 2.05–2.14 (m, 2H), 3.63 (d, *J* = 10.4 Hz, 2H), 3.79 (s, 2H), 3.91 (d, *J* = 10.4 Hz, 2H), 3.94 (s, 3H), 7.53 (dd, *J* = 1.6, 8.4 Hz, 1H), 7.64 (d, *J* = 1.6 Hz, 1H), 7.77 (d, *J* = 8.4 Hz, 1H). MS (ESI) *m*/*z*: 292.9 ([M + H]^+^).

##### Methyl 4-Nitro-3-(2-(trifluoromethyl)morpholino)benzoate (**3i**)

Synthesized according to general procedure A with 2-(trifluoromethyl)morpholine (701 mg, 4.52 mmol) as the reactant and stirring the reaction mixture overnight. The crude product was used in the next step without further purification.

##### Methyl 4-Nitro-3-(pyrrolidin-1-yl)benzoate (**3j**)

Yield: 1.25 g (99.5%); orange crystals. ^1^H NMR (400 MHz, DMSO-*d*_6_): δ 1.87–1.96 (m, 4H), 3.16–3.19 (m, 4H), 3.88 (s, 3H), 7.24 (dd, *J* = 1.7, 8.5 Hz, 1H), 7.53 (d, *J* = 1.7 Hz, 1H), 7.83 (d, *J* = 8.5 Hz, 1H). MS (ESI) *m*/*z*: 250.6 ([M + H]^+^).

##### Methyl (*S*)-3-(3-((*tert*-Butoxycarbonyl)amino)pyrrolidin-1-yl)-4-nitrobenzoate (**3k**)

Synthesized according to general procedure A. Additional details on the experimental procedure, yield, and analytical data for **3k** were previously described in the authors’ patent application.^42^

##### Methyl (*S*)-3-(3-(Dimethylamino)pyrrolidin-1-yl)-4-nitrobenzoate (**3l**)

Synthesized according to general procedure A using (*S*)-*N*,*N*-dimethylpyrrolidin-3-amine (1.07 mL, 4.82 mmol) as the reactant and stirring the reaction mixture overnight. Yield: 1.17 g (98.8%); orange solid. ^1^H NMR (400 MHz, DMSO-*d*_6_): δ 1.71–1.85 (m, 1H), 2.11–2.16 (m, 1H), 2.18 (s, 6H), 2.68–2.81 (m, 1H), 3.08–3.26 (m, 3H), 3.33–3.40 (m, 1H), 3.88 (s, 3H), 7.26 (dd, *J* = 1.7, 8.5 Hz, 1H), 7.52 (d, *J* = 1.7 Hz, 1H), 7.83 (d, *J* = 8.5 Hz, 1H). MS (ESI) *m*/*z*: 293.6 ([M + H]^+^).

##### Methyl 4-Nitro-3-(2-oxooxazolidin-3-yl)benzoate (**3m**)

Synthesized according to general procedure A using oxazolidin-2-one (315 mg, 3.61 mmol) as the reactant and stirring the reaction mixture at 70 °C overnight. The crude product was purified with flash column chromatography using hexane/ethyl acetate (1:1) as the eluent. Yield: 220 mg (50.3%); pale-yellow crystals. ^1^H NMR (400 MHz, DMSO-*d*_6_): δ 3.93 (s, 3H), 4.23 (dd, *J* = 6.9, 8.7 Hz, 2H), 4.54 (dd, *J* = 6.8, 8.8 Hz, 2H), 8.02 (dd, *J* = 1.8, 8.5 Hz, 1H), 8.11–8.18 (m, 2H). MS (ESI) *m*/*z*: 288.5 ([M + Na]^+^).

##### Methyl 3-(2-(Methoxymethyl)pyrrolidin-1-yl)-4-nitrobenzoate (**3n**)

Synthesized according to general procedure B with (methyl 3-(2-methoxymethyl)pyrrolidin-1-yl)-4-nitrobenzoate (1.00 g, 8.68 mmol) as the reactant and stirring the reaction mixture at 60 °C overnight. Yield: 2.35 g (97.0%); orange solid. ^1^H NMR (400 MHz, DMSO-*d*_6_): δ 1.64–1.84 (m, 2H), 1.92 (s, 1H), 2.14–2.21 (m, 1H), 2.43–2.48 (m, 1H), 2.69–2.77 (m, 1H), 3.21 (s, 3H), 3.27–3.32 (m, 1H), 3.37–3.45 (m, 1H), 3.88 (s, 3H), 4.15 (p, *J* = 6.6 Hz, 1H), 7.29 (dd, *J* = 1.7, 8.5 Hz, 1H), 7.75 (d, *J* = 1.7 Hz, 1H), 7.85 (d, *J* = 8.5 Hz, 1H).

##### Methyl (*S*)-3-(2-(Methoxymethyl)pyrrolidin-1-yl)-4-nitrobenzoate (**3o**)

Synthesized according to general procedure B with (methyl (*S*)-3-(2-methoxymethyl)pyrrolidin-1-yl)-4-nitrobenzoate (1.50 g, 9.90 mmol) as the reactant and stirring the reaction mixture at 40 °C for 48 h. The solvent in the reaction mixture was removed in vacuo; to the residue, ethyl acetate (50 mL) was added which was washed with saturated NaHCO_3_ solution (2 × 30 mL) and brine (2 × 30 mL). The organic phase was dried over Na_2_SO_4_, filtered, and the solvent was removed in vacuo. Yield: 2.73 g (98.8%); orange oil. ^1^H NMR (400 MHz, DMSO-*d*_6_): δ 1.66–1.84 (m, 2H), 1.87–1.96 (m, 1H), 2.14–2.23 (m, 1H), 2.43–2.47 (m, 1H), 2.70–2.77 (m, 1H), 3.21 (s, 3H), 3.27–3.31 (m, 1H), 3.38–3.45 (m, 1H), 3.88 (s, 3H), 4.15 (p, *J* = 6.7 Hz, 1H), 7.29 (dd, *J* = 1.7, 8.5 Hz, 1H), 7.75 (d, *J* = 1.7 Hz, 1H), 7.85 (d, *J* = 8.5 Hz, 1H).

##### Methyl 3-(4-Hydroxypiperidin-1-yl)-4-nitrobenzoate (**3p**)

Synthesized according to general procedure B with methyl 3-(4-hydroxypiperidin-1-yl)-4-nitrobenzoate (500 mg, 4.94 mmol) as the reactant. Yield: 1.056 g (76.2%); yellow solid. ^1^H NMR (400 MHz, DMSO-*d*_6_): δ 1.43–1.53 (m, 2H), 1.78–1.85 (m, 2H), 2.84–2.92 (m, 2H), 3.13–3.20 (m, 2H), 3.61–3.68 (m, 1H), 3.89 (s, 3H), 4.75 (s, 1H), 7.58 (dd, *J* = 1.7, 8.4 Hz, 1H), 7.75 (d, *J* = 1.8 Hz, 1H), 7.90 (d, *J* = 8.4 Hz, 1H). MS (ESI) *m*/*z*: 281.0 ([M + H]^+^).

##### Methyl 3-(4-Acetoxypiperidin-1-yl)-4-nitrobenzoate (**3p.1**)

To methyl 3-(4-hydroxypiperidin-1-yl)-4-nitrobenzoate (**3p**, 1.06 g, 3.77 mmol) dissolved in acetonitrile (15 mL), pirydine (450 μL, 5.7 mmol) and acetic anhydride (0.82 mL, 8.7 mmol) were added, and the reaction mixture was stirred at 70 °C overnight. Additional 1.5 equiv of acetic anhydride was added, and the reaction mixture was stirred for 48 h. The solvent was evaporated in vacuo, and to the residue, ethyl acetate (50 mL) was added which was washed with 1% citric acid (30 mL), saturated NaHCO_3_ solution (5 × 50 mL), and brine (5 × 50 mL). The organic phase was dried over Na_2_SO_4_, filtered, and the solvent was removed under reduced pressure. Yield: 1.10 g (90.5%); orange solid. ^1^H NMR (400 MHz, DMSO-*d*_6_): δ 1.61–1.72 (m, 2H), 1.90–1.98 (m, 2H), 2.03 (s, 3H), 2.97–3.06 (m, 2H), 3.13–3.21 (m, 2H), 3.89 (s, 3H), 4.85 (sept, *J* = 4.0 Hz, 1H), 7.63 (dd, *J* = 1.7, 8.4 Hz, 1H), 7.79 (d, *J* = 1.7 Hz, 1H), 7.93 (d, *J* = 8.4 Hz, 1H).

##### *tert*-Butyl 4-(5-(Methoxycarbonyl)-2-nitrophenyl)piperazine-1-carboxylate (**3q**)

Synthesized according to general procedure A using *N*-Boc-piperazine (1.12 g, 6.03 mmol) as the reactant and stirring the reaction mixture overnight. The crude product was filtered through a plug of silica (50 mL), using ethyl acetate as the eluent. Yield: 2.00 g (100%); orange oil. ^1^H NMR (400 MHz, CDCl_3_): δ 1.48 (s, 9H), 3.01–3.09 (m, 4H), 3.55–3.62 (m, 4H), 3.95 (s, 3H), 7.72 (dd, *J* = 1.6, 8.4 Hz, 1H), 7.77 (d, *J* = 8.4 Hz, 1H), 7.82 (d, *J* = 1.7 Hz, 1H).

##### Methyl 4-Nitro-3-(1,4-dioxa-8-azaspiro[4.5]decan-8-yl)benzoate (**3r**)

Synthesized according to general procedure B with (1,4-dioxa-8-azaspiro[4.5]decane) (705 μL, 5.5 mmol) as the reactant. Yield: 1.454 g (90%); orange solid. ^1^H NMR (400 MHz, CDCl_3_): δ 1.87 (m, 4H), 3.19 (m, 4H), 3.94 (s, 3H), 4.00 (s, 4H), 7.64 (dd, *J* = 1.7, 8.4 Hz, 1H), 7.76 (d, *J* = 8.4 Hz, 1H), 7.84 (d, *J* = 1.7 Hz, 1H). MS (ESI) *m*/*z*: 323.0 ([M + H]^+^).

##### Methyl 4-Nitro-3-(1-oxidothiomorpholino)benzoate (**3s**)

Synthesized according to general procedure A with 1-oxidothiomorpholine (844 mg, 5.42 mmol) as the reactant. Yield: 1.10 g (81.8%); light orange crystals. ^1^H NMR (500 MHz, DMSO-*d*_6_): δ 2.82–2.92 (m, 2H), 3.00 (ddd, *J* = 3.3, 11.2, 14.1 Hz, 2H), 3.14–3.24 (m, 2H), 3.66 (ddd, *J* = 2.0, 11.1, 13.3 Hz, 2H), 3.92 (s, 3H), 7.73 (dd, *J* = 1.7, 8.4 Hz, 1H), 7.91 (d, *J* = 1.7 Hz, 1H), 8.01 (d, *J* = 8.4 Hz, 1H). ^13^C{^1^H} NMR (126 MHz, DMSO-*d*_6_): δ 43.30, 45.61, 53.31, 123.64, 123.69, 126.50, 134.46, 145.63, 146.14, 165.31. MS (ESI) *m*/*z*: 299.0 ([M + H]^+^).

##### Methyl 3-((3-Methoxypropyl)amino)-4-nitrobenzoate (**3t**)

Synthesized according to general procedure A with 3-methoxyproplyamine (1.53 mL, 15 mmol) as the reactant and stirring the reaction mixture at rt overnight. During extraction, water was extracted with ethyl acetate (4 × 50 mL). Combined organic phases were washed with brine, dried over Na_2_SO_4_, filtered, and the solvent was removed in vacuo. Yield: 2.68 g (79.6%); orange crystals. ^1^H NMR (400 MHz, CDCl_3_): δ 1.95–2.09 (m, 2H), 3.40 (s, 3H), 3.49 (td, *J* = 5.1, 6.6 Hz, 2H), 3.52–3.60 (m, 2H), 3.94 (s, 3H), 7.22 (dd, *J* = 1.7, 8.9 Hz, 1H), 7.59 (d, *J* = 1.7 Hz, 1H), 8.22 (d, *J* = 8.9 Hz, 1H), 8.29 (br s, 1H). MS (ESI) *m*/*z*: 269.0 ([M + H]^+^).

##### Methyl 3-((*tert*-Butoxycarbonyl)(3-methoxypropyl)amino)-4-nitrobenzoate (**3t.1**)

**3t** (2.68 g, 10.0 mmol) and DMAP (244 mg, 2.0 mmol) were dissolved in THF (40 mL) followed by addition of di-*tert*-butyl dicarbonate (3.27 g, 15.0 mmol). The reaction mixture was stirred at 40 °C overnight. The solvent was removed under reduced pressure. The residue was dissolved in ethyl acetate and washed with 1% citric acid and brine. The organic phase was dried over Na_2_SO_4_, filtered, and solvent was removed under reduced pressure. Yield: 3.68 g (100%); yellow oil. ^1^H NMR (400 MHz, CDCl_3_): δ 1.47 (s, 9H), 1.99 (m, 2H), 3.29 (s, 3H), 3.45 (td, *J* = 3.5, 5.9 Hz, 2H), 3.72–3.87 (m, 2H), 3.97 (s, 3H), 7.94 (d, *J* = 8.5 Hz, 1H), 7.98–8.07 (m, 2H).

#### General Procedure C: Synthesis of **4a–f**, **4i–r**, **4t**, and **11** (with **4j** as an Example)

Compound **3j** (1.20 g, 4.80 mmol) was dissolved in methanol under an argon atmosphere. Pd/C (10%, 120 mg) was added, and the reaction mixture was purged with hydrogen. Then, it was stirred under a hydrogen atmosphere at rt for 3 h. The reaction mixture was filtered through celite, and the solvent was evaporated in vacuo to get **4j** as yellow oil.

##### Methyl 4-Amino-3-morpholinobenzoate (**4a**)

Synthesized according to general procedure C with **3a** (676 mg, 2.54 mmol) as the reactant. Yield: 565 mg (94.2%); colorless oil. ^1^H NMR (400 MHz, DMSO-*d*_6_): δ 2.77–2.79 (m, 4H), 3.74 (s, 3H), 3.75–3.77 (m, 4H), 5.68 (s, 2H), 6.70 (d, *J* = 8.3 Hz, 1H), 7.45–7.49 (m, 2H). MS (ESI) *m*/*z*: 234.7 ([M – H]^−^).

##### Methyl 4-Amino-3-(3-methylmorpholino)benzoate (**4b**)

Synthesized according to general procedure C with crude **3b** (1.27 g) as the reactant and stirring the reaction mixture for 2 h. The crude product was purified with flash column chromatography using hexane/ethyl acetate 2:1 as the eluent. Yield: 316 mg (27.9%); white crystals. ^1^H NMR (400 MHz, DMSO-*d*_6_): δ 0.69 (d, *J* = 6.2 Hz, 3H), 2.57–2.63 (m, 1H), 2.76 (d, *J* = 11.3 Hz, 1H), 3.03–3.11 (m, 1H), 3.27 (dd, *J* = 8.9, 10.9 Hz, 1H), 3.68–3.73 (m, 2H), 3.74 (s, 3H), 3.81 (dd, *J* = 3.0, 10.9 Hz, 1H), 5.81 (s, 2H), 6.70 (d, *J* = 8.4 Hz, 1H), 7.42–7.60 (m, 2H). MS (ESI) *m*/*z*: 251.2 ([M + H]^+^).

##### Methyl (*R*)-4-Amino-3-(3-methylmorpholino)benzoate (**4c**)

Synthesized according to general procedure C with **3c** (1.00 g, 3.60 mmol) as the reactant. Yield: 0.7 g (77.8%); white solid. ^1^H NMR (400 MHz, DMSO-*d*_6_): δ 0.69 (d, *J* = 6.2 Hz, 3H), 2.45 (m, 1H, signal is overlapped with the signal for DMSO), 2.54–2.63 (m, 2H), 2.76 (d, *J* = 11.8 Hz, 1H), 3.05–3.09 (m, 1H), 3.27 (dd, *J* = 8.9, 10.9 Hz, 1H), 3.68–3.77 (m, 5H), 3.81 (dd, *J* = 3.1, 10.9 Hz, 1H), 5.82 (s, 2H), 6.69 (d, *J* = 8.4 Hz, 1H), 7.50 (dd, *J* = 2.0, 8.4 Hz, 1H), 7.54 (d, *J* = 2.0 Hz, 1H).

##### Methyl (*S*)-4-Amino-3-(3-methylmorpholino)benzoate (**4d**)

Synthesized according to general procedure C with **3d** (3.80 g, 13.6 mmol) as the reactant. The crude product was purified with flash column chromatography using ethyl acetate/hexane 1:2 as the eluent. Yield: 2.26 g (66.6%); white solid. ^1^H NMR (400 MHz, DMSO-*d*_6_): δ 0.69 (d, *J* = 6.2 Hz, 3H), 2.52 (m, 2H), 2.54–2.64 (m, 1H), 2.76 (d, *J* = 11.7 Hz, 1H), 3.05–3.11 (m, 1H), 3.27 (dd, *J* = 8.9, 10.9 Hz, 1H), 3.68–3.77 (m, 5H), 3.81 (dd, *J* = 3.1, 10.9 Hz, 1H), 5.82 (s, 2H), 6.69 (d, *J* = 8.4 Hz, 1H), 7.50 (dd, *J* = 2.0, 8.4 Hz, 1H), 7.54 (d, *J* = 2.0 Hz, 1H). MS (ESI) *m*/*z*: 250.9 ([M + H]^+^).

##### Methyl 4-Amino-3-(2-methylmorpholino)benzoate (**4e**)

Synthesized according to general procedure C with **3e** (1.57 g, 5.60 mmol) as the reactant. Yield: 1.39 g (99.1%); white crystals. ^1^H NMR (400 MHz, DMSO-*d*_6_): δ 1.10 (d, *J* = 6.2 Hz, 3H), 2.32 (dd, *J* = 9.7, 11.3 Hz, 1H), 2.58 (td, *J* = 3.3, 11.3 Hz, 1H), 2.82–2.96 (m, 2H), 3.69–3.87 (m, 6H), 5.67 (s, 2H), 6.70 (d, *J* = 8.3 Hz, 1H), 7.40–7.52 (m, 2H). MS (ESI) *m*/*z*: 250.7 ([M + H]^+^).

##### Methyl 4-Amino-3-(2,6-dimethylmorpholino)benzoate (**4f**)

Synthesized according to general procedure C with **3f** (1.60 g, 5.44 mmol) as the reactant. Yield: 1.42 g (98.8%); white crystals. ^1^H NMR (400 MHz, DMSO-*d*_6_): δ 1.10 (d, *J* = 6.3 Hz, 6H), 2.22 (dd, *J* = 9.9, 11.4 Hz, 2H), 2.89–2.95 (m, 2H), 3.74 (s, 4H), 3.77–3.85 (m, 2H), 5.66 (s, 2H), 6.69 (d, *J* = 8.3 Hz, 1H), 7.42 (d, *J* = 1.9 Hz, 1H), 7.47 (dd, *J* = 1.9, 8.3 Hz, 1H). ^13^C{^1^H} NMR (101 MHz, DMSO-*d*_6_): δ 19.24, 51.71, 56.89, 71.76, 113.63, 116.98, 120.93, 127.11, 136.93, 148.12, 166.84. MS (ESI) *m*/*z*: 264.6 ([M + H]^+^).

##### Methyl 4-Amino-3-((1*R*,5*S*)-8-oxa-3-azabicyclo[3.2.1]octan-3-yl)benzoate (**4g**)

Compound **3g** (1.11 g, 3.80 mmol) was dissolved in acetic acid (35 mL), iron (2.12 g, 37.98 mmol) was added, and the reaction mixture was stirred at rt overnight. To the reaction mixture, water (25 mL) was added, and the excess iron was filtered over celite and flushed with water. The precipitate that formed in the filtrate was filtered off to give 180 mg of the product. The excess of the product that crystallized on the celite was dissolved in ethyl acetate (200 mL). The organic phase was washed with saturated NaHCO_3_ solution (5 × 50 mL) and brine (5 × 50 mL), dried over Na_2_SO_4_, and concentrated in vacuo to obtain 520 mg of the product as a white solid. Yield: 0.700 g (70.3%); white solid. ^1^H NMR (500 MHz, DMSO-*d*_6_): δ 1.85 (tq, *J* = 5.6, 6.4, 9.5 Hz, 2H), 2.07 (t, *J* = 6.4 Hz, 2H), 2.74 (d, *J* = 11.0 Hz, 2H), 2.81 (dd, *J* = 2.0, 11.3 Hz, 2H), 3.76 (s, 3H), 4.34 (dd, *J* = 2.4, 4.7 Hz, 2H), 5.51 (s, 2H), 6.75 (d, *J* = 8.1 Hz, 1H), 7.47–7.53 (m, 2H). MS (ESI) *m*/*z*: 263.1 ([M + H]^+^).

##### Methyl 4-Amino-3-((1*R*,5*S*)-3-oxa-8-azabicyclo[3.2.1]octan-8-yl)benzoate (**4h**)

Compound **3h** (1.69 g, 5.85 mmol) was dissolved in acetic acid (55 mL), iron (3.27 g, 58.5 mmol) was added, and the reaction mixture was stirred at rt overnight. To the reaction mixture, a few drops of water were added, and the excess iron was filtered over celite and flushed with water. The precipitate that formed in the filtrate was filtered off to give 80 mg of the product. The rest of the product was extracted from the filtrate with ethyl acetate (200 mL). The organic phase was washed with saturated NaHCO_3_ solution (3 × 50 mL) and brine (2 × 50 mL), dried over Na_2_SO_4_, and concentrated in vacuo to obtain additional 940 mg of the product. Yield: 1.02 g (66%); white crystals. ^1^H NMR (400 MHz, CDCl_3_): δ 2.05 (d, *J* = 2.0 Hz, 4H), 3.63 (s, 2H), 3.70 (dd, *J* = 2.0, 10.4 Hz, 2H), 3.85 (s, 3H), 3.89 (d, *J* = 10.4 Hz, 2H), 4.30 (s, 2H), 6.70 (d, *J* = 8.2 Hz, 1H), 7.47 (d, *J* = 1.8 Hz, 1H), 7.60 (dd, *J* = 1.8, 8.2 Hz, 1H). MS (ESI) *m*/*z*: 263.0 ([M + H]^+^).

##### Methyl 4-Amino-3-(2-(trifluoromethyl)morpholino)benzoate (**4i**)

Synthesized according to general procedure C with crude **3i** (1.26 g) as the reactant. Yield: 330 mg (28.8%); white crystals. ^1^H NMR (400 MHz, DMSO-*d*_6_): δ 2.58 (t, *J* = 10.7 Hz, 1H), 2.79–2.93 (m, 2H), 3.10–3.17 (m, 1H), 3.75 (s, 3H), 3.80–3.90 (m, 1H), 4.00–4.08 (m, 1H), 4.47–4.55 (m, 1H), 5.83 (s, 2H), 6.71 (d, *J* = 8.2 Hz, 1H), 7.47–7.54 (m, 2H). MS (ESI) *m*/*z*: 304.9 ([M + H]^+^).

##### Methyl 4-Amino-3-(pyrrolidin-1-yl)benzoate (**4j**)

Yield: 1.0 g (94.7%); yellow oil. ^1^H NMR (400 MHz, DMSO-*d*_6_): δ 1.79–1.90 (m, 4H), 2.94–2.97 (m, 4H), 3.73 (s, 3H), 5.53 (s, 2H), 6.66 (d, *J* = 8.2 Hz, 1H), 7.38–7.45 (m, 2H). ^13^C{^1^H} NMR (101 MHz, DMSO-*d*_6_): δ 23.99, 50.40, 51.67, 113.62, 116.82, 119.23, 125.83, 135.87, 147.91, 166.99. MS (ESI) *m*/*z*: 220.8 ([M + H]^+^).

##### Methyl (*S*)-4-Amino-3-(3-((*tert*-butoxycarbonyl)amino)pyrrolidin-1-yl)benzoate (**4k**)

Synthesized according to general procedure C. Additional details on the experimental procedure, yield, and analytical data for **4k** were previously described in the authors’ patent application.^42^

##### Methyl (*S*)-4-Amino-3-(3-(dimethylamino)pyrrolidin-1-yl)benzoate (**4l**)

Synthesized according to general procedure C with **3l** (1.121 g, 3.82 mmol) as the reactant. Yield: 1.01 g (100%); green oil. ^1^H NMR (400 MHz, DMSO-*d*_6_): δ 1.67–1.83 (m, 1H), 1.96–2.11 (m, 1H), 2.16 (s, 6H), 2.74–2.94 (m, 3H), 3.05–3.24 (m, 2H), 3.74 (s, 3H), 5.50 (s, 2H), 6.67 (d, *J* = 8.1 Hz, 1H), 7.41–7.44 (m, 2H). MS (ESI) *m*/*z*: 263.7 ([M + H]^+^).

##### Methyl 4-Amino-3-(2-oxooxazolidin-3-yl)benzoate (**4m**)

Synthesized according to general procedure C with **3m** (200 mg, 0.751 mmol) as the reactant. Yield: 170 mg (95.8%); white crystals. ^1^H NMR (400 MHz, DMSO-*d*_6_): δ 3.77–3.81 (m, 2H), 4.40–4.48 (m, 2H), 6.20 (s, 2H), 6.74 (d, *J* = 8.5 Hz, 1H), 7.64 (dd, *J* = 2.1, 8.5 Hz, 1H), 7.68 (d, *J* = 2.0 Hz, 1H). ^13^C{^1^H} NMR (101 MHz, DMSO-*d*_6_): δ 46.32, 51.84, 62.78, 114.86, 116.62, 121.71, 130.61, 130.84, 150.71, 157.40, 166.27. MS (ESI) *m*/*z*: 236.7 ([M + H]^+^).

##### Methyl 4-Amino-3-(2-(methoxymethyl)pyrrolidin-1-yl)benzoate (**4n**)

Synthesized according to general procedure C with **3n** (2.35 g, 8.0 mmol) as the reactant. The crude product was purified with flash column chromatography using ethyl acetate/hexane 1:3 as the eluent. Yield: 1.36 g (64.4%); white solid. ^1^H NMR (400 MHz, DMSO-*d*_6_): δ 1.63–1.75 (m, 1H), 1.76–1.91 (m, 2H), 2.02–2.14 (m, 1H), 2.59–2.67 (m, 1H), 3.04 (dd, *J* = 7.0, 9.4 Hz, 1H), 3.11–3.18 (m, 4H), 3.35–3.38 (m, 1H), 3.49–3.59 (m, 1H), 3.73 (s, 3H), 5.65 (s, 2H), 6.66 (d, *J* = 8.4 Hz, 1H), 7.44 (dd, *J* = 2.0, 8.4 Hz, 1H), 7.58 (d, *J* = 2.0 Hz, 1H).

##### Methyl (*S*)-4-Amino-3-(2-(methoxymethyl)pyrrolidin-1-yl)benzoate (**4o**)

Synthesized according to general procedure C with **3o** (2.70 g, 9.17 mmol) as the reactant. Yield: 2.19 g (61.1%); gray solid. ^1^H NMR (400 MHz, DMSO-*d*_6_): δ 1.65–1.73 (m, 1H), 1.78–1.91 (m, 2H), 2.04–2.13 (m, 1H), 2.59–2.64 (m, 1H), 3.04 (dd, *J* = 6.9, 9.4 Hz, 1H), 3.13–3.17 (m, 4H), 3.36–3.40 (m, 1H), 3.50–3.57 (m, 1H), 3.73 (s, 3H), 5.65 (s, 2H), 6.66 (d, *J* = 8.4 Hz, 1H), 7.44 (dd, *J* = 1.9, 8.3 Hz, 1H), 7.58 (d, *J* = 2.0 Hz, 1H).

##### Methyl 3-(4-Acetoxypiperidin-1-yl)-4-aminobenzoate (**4p**)

Synthesized according to general procedure C with **3p.1** (1.10 g, 3.40 mmol) as the reactant. Yield: 0.70 g (70.2%); gray solid. ^1^H NMR (400 MHz, DMSO-*d*_6_): δ 1.74–1.83 (m, 2H), 1.92–1.99 (m, 2H), 2.04 (s, 3H), 2.67–2.75 (m, 2H), 2.90–2.97 (m, 2H), 3.74 (s, 3H), 4.81 (s, 1H), 5.65 (s, 2H), 6.68 (d, *J* = 8.9 Hz, 1H), 7.45–7.49 (m, 2H).

##### *tert*-Butyl 4-(2-Amino-5-(methoxycarbonyl)phenyl)piperazine-1-carboxylate (**4q**)

Synthesized according to general procedure C with **3q** (1.90 g, 5.20 mmol) as the reactant and stirring the reaction mixture overnight. Yield: 1.65 g, (95%); white solid. ^1^H NMR (400 MHz, CDCl_3_): δ 1.49 (s, 9H), 2.79–2.93 (m, 6H), 3.31–3.78 (m, 2H), 3.85 (s, 3H), 4.40 (s, 2H), 6.70 (d, *J* = 8.7 Hz, 1H), 7.63–7.71 (m, 2H).

##### Methyl 4-Amino-3-(1,4-dioxa-8-azaspiro[4.5]decan-8-yl)benzoate (**4r**)

Synthesized according to general procedure C with **3r** (1.44 g, 4.47 mmol) as the reactant. Yield: 1.29 g (98.8%); white solid. ^1^H NMR (400 MHz, CDCl_3_): δ 1.87 (t, *J* = 5.6 Hz, 4H), 2.99 (dd, *J* = 4.3, 6.9 Hz, 4H), 3.84 (s, 3H), 4.01 (s, 4H), 4.41 (s, 2H), 6.69 (d, *J* = 8.3 Hz, 1H), 7.65 (dd, *J* = 1.9, 8.3 Hz, 1H), 7.75 (d, *J* = 1.9 Hz, 1H). MS (ESI) *m*/*z*: 293.0 ([M + H]^+^).

##### Methyl 4-Amino-3-(1-oxidothiomorpholino)benzoate (**4s**)

Compound **3s** (1.10 g, 3.70 mmol) was dissolved in acetic acid (40 mL), iron (2.06 g, 36.98 mmol) was added, and the reaction mixture was stirred at rt overnight. To the reaction mixture, water (25 mL) was added, and the excess iron was filtered over celite and flushed with water. The filtrate was concentrated under reduced pressure, and ethyl acetate was added. The organic phase was washed with saturated NaHCO_3_ solution and brine, dried over Na_2_SO_4_, and concentrated in vacuo. Yield: 0.706 g (71.2%); opaque powder. ^1^H NMR (500 MHz, CDCl_3_): δ 2.82–3.27 (m, 6H), 3.63 (s, 2H), 3.79 (s, 3H), 4.44 (s, 2H), 6.67 (d, *J* = 8.4 Hz, 1H), 7.66 (dd, *J* = 1.9, 8.4 Hz, 1H), 7.75 (s, 1H). MS (ESI) *m*/*z*: 269.1 ([M + H]^+^).

##### Methyl 4-Amino-3-((*tert*-butoxycarbonyl)(3-methoxypropyl)amino)benzoate (**4t**)

Synthesized according to general procedure C with **3t.1** (3.68 g, 10.0 mmol) as the reactant. Yield: 3.17 g (93.8%); yellow oil. ^1^H NMR (400 MHz, CDCl_3_): δ 1.39 (s, 9H), 1.80 (dq, *J* = 5.8, 6.3, 11.6 Hz, 2H), 3.29 (s, 3H), 3.45 (t, *J* = 5.8 Hz, 2H), 3.55–3.75 (m, 2H), 3.85 (s, 3H), 4.20–4.50 (m, 2H), 6.70 (d, *J* = 8.4 Hz, 1H), 7.69 (br s, 1H), 7.76 (dd, *J* = 2.0, 8.4 Hz, 1H).

#### General Procedure D: Synthesis of Compounds **5a**, **5b**, **5e–m**, **5q**, **5s**, **5t**, and **12** (with **5b** as an Example)

Compound **4b** (310 mg, 1.24 mmol) and KSCN (361 mg, 3.72 mmol) were dissolved in glacial acetic acid (15 mL), stirred for 30 min, cooled to 10 °C, and bromine (96 μL, 1.86 mmol) was added dropwise. The reaction mixture was stirred at rt overnight. The reaction mixture was neutralized with 25% aq NH_3_ solution to pH = 9, and the precipitate was filtered off. The precipitate was suspended in methanol, heated, and filtered out of the hot suspension to wash the product in methanol. The procedure was repeated three times. Methanol was evaporated, and the residue was suspended in cold methanol, filtered off, and dried to give **5b** as pale-yellow crystals.

##### Methyl 2-Amino-4-morpholinobenzo[*d*]thiazole-6-carboxylate (**5a**)

Synthesized according to general procedure D with **4a** (550 mg, 2.33 mmol) as the reactant. 4 equiv of KSCN (0.905 g, 9.32 mmol) and 2 equiv of bromine (239 μL, 4.66 mmol) were used instead of 3 and 1.5 equiv. The crude product was additionally purified with flash column chromatography using ethyl acetate/hexane (1:1) as the eluent. Yield: 259 mg (37.9%), pale-yellow solid. ^1^H NMR (400 MHz, DMSO-*d*_6_): δ 3.27 (t, *J* = 4.7 Hz, 4H), 3.77 (t, *J* = 4.6 Hz, 4H), 3.82 (s, 3H), 7.28 (d, *J* = 1.6 Hz, 1H), 7.84 (s, 2H), 7.94 (d, *J* = 1.6 Hz, 1H). ^13^C{^1^H} NMR (101 MHz, DMSO-*d*_6_): δ 50.42, 52.31, 66.80, 113.56, 116.46, 122.72, 132.27, 141.92, 148.60, 166.80, 168.12. MS (ESI) *m*/*z*: 291.5 ([M – H]^−^).

##### Methyl 2-Amino-4-(3-methylmorpholino)benzo[*d*]thiazole-6-carboxylate (**5b**)

Yield: 251 mg (65.9%), pale-yellow crystals. ^1^H NMR (400 MHz, DMSO-*d*_6_): δ 0.85 (d, *J* = 6.6 Hz, 3H), 2.88–2.98 (m, 1H), 3.52 (dd, *J* = 3.8, 11.0 Hz, 1H), 3.60–3.71 (m, 1H), 3.77–3.93 (m, 5H), 4.43 (s, 1H), 7.29 (d, *J* = 1.6 Hz, 1H), 7.84 (s, 2H), 7.95 (d, *J* = 1.6 Hz, 1H). ^13^C{^1^H} NMR (101 MHz, DMSO-*d*_6_): δ 12.49, 46.12, 50.71, 52.33, 67.10, 71.73, 116.69, 116.81, 122.63, 132.44, 140.54, 149.61, 166.79, 168.13. MS (ESI) *m*/*z*: 307.9 ([M + H]^+^).

##### Methyl (*R*)-2-Amino-4-(3-methylmorpholino)benzo[*d*]thiazole-6-carboxylate (**5c**)

KSCN (1.09 g, 11.2 mmol) was dissolved in acetic acid (14 mL) under an argon atmosphere, followed by the addition of Br_2_ (460 μL, 5.59 mmol). The reaction mixture was stirred for 30 min and then added dropwise to compound **4c** (0.70 g, 2.80 mmol) in acetic acid (14 mL). The reaction mixture was stirred at rt under an argon atmosphere overnight. The reaction mixture was neutralized with 2 M NaOH to pH = 9, and the precipitate was filtered off. The precipitate was suspended in methanol, heated, and filtered out of the hot suspension to wash the product in methanol. The procedure was repeated two times. Methanol was evaporated, and the residue was purified with reverse-phase flash chromatography on a Biotage Isolera One System using a Biotage SNAP Cartridge KP-C18-HS column and acetonitrile/0.1% trifluoroacetic acid mixture as a mobile phase. Acetonitrile was evaporated, and trifluoroacetic acid was neutralized with a few drops of saturated NaHCO_3_ solution. The solution was extracted with ethyl acetate (3 × 50 mL), the combined organic phases were washed with brine (2 × 50 mL), dried over Na_2_SO_4_, and the solvent was evaporated under reduced pressure. Yield: 80 mg (9.3%), orange solid. ^1^H NMR (400 MHz, DMSO-*d*_6_): δ 0.85 (d, *J* = 6.5 Hz, 3H), 2.52 (d, *J* = 1.9 Hz, 2H, signal is overlapped with the signal for DMSO), 2.92 (dt, *J* = 3.1, 11.9 Hz, 1H), 3.27–3.32 (m, 1H, signal is overlapped with the signal for water), 3.52 (dd, *J* = 3.9, 11.0 Hz, 1H), 3.65 (ddd, *J* = 2.3, 8.6, 11.1 Hz, 1H), 3.79–3.88 (m, 5H), 4.38–4.50 (m, 1H), 7.29 (d, *J* = 1.7 Hz, 1H), 7.85 (s, 2H), 7.95 (d, *J* = 1.6 Hz, 1H). MS (ESI) *m*/*z*: 307.8 ([M + H]^+^).

##### Methyl (*S*)-2-Amino-4-(3-methylmorpholino)benzo[*d*]thiazole-6-carboxylate (**5d**)

KSCN (3.51 g, 36.2 mmol) was dissolved in acetic acid (40 mL) under an argon atmosphere, followed by the addition of Br_2_ (0.96 mL, 18.1 mmol). The reaction mixture was stirred for 30 min and then added dropwise to compound **4d** (2.26 g, 9.04 mmol) in acetic acid (45 mL). The reaction mixture was stirred at rt under an argon atmosphere overnight. The reaction mixture was neutralized with 2 M NaOH to pH = 9, and the precipitate was filtered off. The precipitate was suspended in methanol, heated, and filtered out of the hot suspension to wash the product in methanol. The procedure was repeated two times. Methanol was evaporated, and the residue was suspended in cold methanol, filtered off, and dried to give **5d** as orange crystals. Yield: 1.77 g (63.8%), orange crystals. ^1^H NMR (400 MHz, DMSO-*d*_6_): δ 0.85 (d, *J* = 6.5 Hz, 3H), 2.88–2.98 (m, 1H), 3.49–3.57 (m, 2H), 3.61–3.70 (m, 2H), 3.78–3.91 (m, 6H), 4.40–4.50 (m, 1H), 7.30 (d, *J* = 1.5 Hz, 1H), 7.86 (s, 2H), 7.94–7.98 (m, 1H). MS (ESI) *m*/*z*: 307.8 ([M + H]^+^).

##### Methyl 2-Amino-4-(2-methylmorpholino)benzo[*d*]thiazole-6-carboxylate (**5e**)

Synthesized according to general procedure D with **4e** (1.38 g, 5.51 mmol) as the reactant. 4 equiv of KSCN (2.14 g, 22.0 mmol) and 2 equiv of bromine (565 μL, 11.0 mmol) were used instead of 3 and 1.5 equiv. After methanol was evaporated, the residue was purified with flash column chromatography using hexane/ethyl acetate 1:2 as the eluent. Yield: 150 mg (8.9%), pale-yellow crystals. ^1^H NMR (400 MHz, DMSO-*d*_6_): δ 1.15 (d, *J* = 6.2 Hz, 3H), 2.39 (dd, *J* = 10.0, 11.4 Hz, 1H), 2.65 (td, *J* = 3.0, 11.4 Hz, 1H), 3.66–3.78 (m, 3H), 3.82 (s, 3H), 3.84–3.94 (m, 2H), 7.27 (d, *J* = 1.7 Hz, 1H), 7.84 (s, 2H), 7.94 (d, *J* = 1.6 Hz, 1H). ^13^C{^1^H} NMR (101 MHz, DMSO-*d*_6_): δ 19.38, 49.83, 52.32, 56.35, 66.53, 71.68, 113.67, 116.46, 122.71, 132.27, 141.78, 148.67, 166.80, 168.12. MS (ESI) *m*/*z*: 305.1 ([M – H]^−^).

##### Methyl 2-Amino-4-(2,6-dimethylmorpholino)benzo[*d*]thiazole-6-carboxylate (**5f**)

Synthesized according to general procedure D with **4f** (1.10 g, 4.16 mmol) as the reactant. The purification procedure using methanol was not performed, and the crude precipitate was purified with flash column chromatography using hexane/ethyl acetate 1:1 as the eluent. Yield: 469 mg (35.1%), pale-yellow crystals. ^1^H NMR (400 MHz, DMSO-*d*_6_): δ 1.15 (d, *J* = 6.1 Hz, 6H), 2.22–2.33 (m, 2H), 3.73–3.84 (m, 7H), 7.26 (d, *J* = 1.7 Hz, 1H), 7.84 (s, 2H), 7.94 (d, *J* = 1.6 Hz, 1H). ^13^C{^1^H} NMR (101 MHz, DMSO-*d*_6_): δ 19.36, 52.32, 55.81, 71.54, 113.79, 116.44, 122.72, 132.27, 141.64, 148.73, 166.81, 168.12. MS (ESI) *m*/*z*: 322.3 ([M + H]^+^).

##### Methyl 2-Amino-4-((1*R*,5*S*)-8-oxa-3-azabicyclo[3.2.1]octan-3-yl)benzo[*d*]thiazole-6-carboxylate (**5g**)

Synthesized according to general procedure D with **4g** (620 mg, 2.36 mmol) as the reactant. Yield: 525 mg (69.6%), yellow crystals. ^1^H NMR (400 MHz, DMSO-*d*_6_): δ 1.77–1.88 (m, 2H), 2.02–2.08 (m, 2H), 2.80–2.89 (m, 2H), 3.72–3.80 (m, 2H), 3.81 (s, 3H), 4.32–4.44 (m, 2H), 7.17 (d, *J* = 1.6 Hz, 1H), 7.76 (s, 2H), 7.89 (d, *J* = 1.5 Hz, 1H). ^13^C{^1^H} NMR (101 MHz, DMSO-*d*_6_): δ 28.45, 52.29, 55.10, 74.11, 113.13, 115.71, 122.77, 132.30, 141.90, 148.13, 166.83, 167.69. MS (ESI) *m*/*z*: 317.6 ([M – H]^−^).

##### Methyl 2-Amino-4-((1*R*,5*S*)-3-oxa-8-azabicyclo[3.2.1]octan-8-yl)benzo[*d*]thiazole-6-carboxylate (**5h**)

Synthesized according to general procedure D with **4h** (1.00 g, 3.82 mmol) as the reactant. The crude product was additionally purified with flash column chromatography using ethyl acetate/hexane = 1:3 as the eluent. Yield 238 mg (20%), yellow powder. ^1^H NMR (400 MHz, DMSO-*d*_6_): δ 1.83–1.96 (m, 4H), 3.51 (d, *J* = 10.7 Hz, 2H), 3.75 (d, *J* = 10.7 Hz, 2H), 3.80 (s, 3H), 4.65 (s, 2H), 7.21 (d, *J* = 1.6 Hz, 1H), 7.72 (s, 2H), 7.78 (d, *J* = 1.6 Hz, 1H). MS (ESI) *m*/*z*: 320.0 ([M + H]^+^).

##### Methyl 2-Amino-4-(2-(trifluoromethyl)morpholino)benzo[*d*]thiazole-6-carboxylate (**5i**)

Synthesized according to general procedure D with **4i** (315 mg, 1.04 mmol) as the reactant. After neutralization, ethyl acetate was added, and the precipitate formed which was filtered out of the two-phase system. The organic and water phase of the mother liquid were separated, organic phase was dried over Na_2_SO_4_, filtered, and solvent was removed in vacuo. The crude product was purified with flash column chromatography using hexane/ethyl acetate 1:1 as the eluent. Yield: 150 mg (40.1%), white solid. ^1^H NMR (400 MHz, DMSO-*d*_6_): δ 2.77 (t, *J* = 10.8 Hz, 1H), 2.86 (td, *J* = 3.3, 11.7 Hz, 1H), 3.77–3.88 (s, 5H), 4.03–4.14 (m, 2H), 4.32–4.41 (m, 1H), 7.32 (d, *J* = 1.7 Hz, 1H), 7.89 (s, 2H), 8.00 (d, *J* = 1.6 Hz, 1H). ^13^C{^1^H} NMR (101 MHz, DMSO-*d*_6_): δ 48.16, 49.34, 52.35, 66.73, 114.23, 117.20, 122.71, 125.53, 128.31, 132.39, 140.80, 148.77, 166.69, 168.53. MS (ESI) *m*/*z*: 359.9 ([M – H]^−^).

##### Methyl 2-Amino-4-(pyrrolidin-1-yl)benzo[*d*]thiazole-6-carboxylate (**5j**)

Synthesized according to general procedure D. Additional details on the experimental procedure, yield, and analytical data for **5j** were previously described in the authors’ patent application.^42^

##### Methyl (*S*)-2-Amino-4-(3-((*tert*-butoxycarbonyl)amino)pyrrolidin-1-yl)benzo[*d*]thiazole-6-carboxylate (**5k**)

Synthesized according to general procedure D. Additional details on the experimental procedure, yield, and analytical data for **5k** were previously described in the authors’ patent application.^42^

##### Methyl (*S*)-2-Amino-4-(3-(dimethylamino)pyrrolidin-1-yl)benzo[*d*]thiazole-6-carboxylate (**5l**)

Synthesized according to general procedure D with **4l** (1.00 g, 3.80 mmol) as the reactant. 4 equiv of KSCN (1.49 g, 15.2 mmol) and 2 equiv of bromine (389 μL, 7.6 mmol) were used instead of 3 and 1.5 equiv. After removing methanol, the crude product was purified with flash column chromatography using DCM/methanol 9:1 as the eluent. Yield: 150 mg (12.3%), pale-yellow crystals. ^1^H NMR (400 MHz, DMSO-*d*_6_): δ 1.81–1.86 (m, 1H), 2.12–2.16 (m, 1H), 2.32 (s, 6H), 3.46–3.67 (m, 4H), 3.74 (dd, *J* = 7.1, 10.0 Hz, 1H), 3.81 (s, 3H), 7.00 (d, *J* = 1.7 Hz, 1H), 7.59 (s, 2H), 7.70 (d, *J* = 1.6 Hz, 1H). MS (ESI) *m*/*z*: 321.2 ([M + H]^+^).

##### Methyl 2-Amino-4-(2-oxooxazolidin-3-yl)benzo[*d*]thiazole-6-carboxylate (**5m**)

Synthesized according to general procedure D with **4m** (170 mg, 0.719 mmol) as the reactant. 4 equiv of KSCN (279 mg, 2.88 mmol) and 2 equiv of bromine (74 μL, 1.44 mmol) were used instead of 3 and 1.5 equiv. After neutralization, ethyl acetate was added, and the phases were separated. The organic phase was dried over Na_2_SO_4_, filtered, and the solvent was removed in vacuo. The crude product was purified twice with flash column chromatography using DCM/methanol (20:1) as the eluent. Yield: 60 mg (28%), white solid. ^1^H NMR (400 MHz, DMSO-*d*_6_): δ 3.84 (s, 3H), 4.16 (dd, *J* = 7.0, 9.0 Hz, 2H), 4.46 (dd, *J* = 6.9, 9.0 Hz, 2H), 7.89 (d, *J* = 1.7 Hz, 1H), 8.16 (s, 2H), 8.26 (d, *J* = 1.7 Hz, 1H). MS (ESI) *m*/*z*: 293.6 ([M + H]^+^).

##### Methyl 2-Amino-4-(2-(methoxymethyl)pyrrolidin-1-yl)benzo[*d*]thiazole-6-carboxylate (**5n**)

KSCN (1.33g, 13.7 mmol) was dissolved in acetic acid (23 mL) under an argon atmosphere, followed by the addition of Br_2_ (370 μL, 6.87 mmol). The reaction mixture was stirred for 30 min and then added dropwise to compound **4n** (1.21g, 4.58 mmol) in acetic acid (23 mL). The reaction mixture was stirred at rt under an argon atmosphere overnight. The reaction mixture was neutralized with 25% aq NH_3_ solution to pH = 9, and the precipitate was filtered off. The precipitate was suspended in methanol, heated, and filtered out of the hot suspension to wash the product in methanol. The procedure was repeated two times. Methanol was evaporated, and the residue was purified with flash column chromatography using ethyl acetate/hexane/acetone 1:3:1 as the eluent. Yield: 430 mg (29.2%), orange crystals. ^1^H NMR (400 MHz, DMSO-*d*_6_): δ 1.82–2.04 (m, 4H), 3.13 (dd, *J* = 7.5, 9.4 Hz, 1H), 3.19 (s, 3H), 3.21–3.26 (m, 1H), 3.29–3.33 (m, 1H), 3.66–3.76 (m, 1H), 3.81 (s, 3H), 4.78–4.88 (m, 1H), 7.08 (d, *J* = 1.7 Hz, 1H), 7.58 (s, 2H), 7.69 (d, *J* = 1.6 Hz, 1H). MS (ESI) *m*/*z*: 322.0 ([M + H]^+^).

##### Methyl (*S*)-2-Amino-4-(2-(methoxymethyl)pyrrolidin-1-yl)benzo[*d*]thiazole-6-carboxylate (**5o**)

KSCN (2.42 g, 24.9 mmol) was dissolved in acetic acid (40 mL) under an argon atmosphere, followed by the addition of Br_2_ (660 μL, 12.4 mmol). The reaction mixture was stirred for 30 min and then added dropwise to compound **4o** (2.19 g, 8.29 mmol) in acetic acid (40 mL). The reaction mixture was stirred at rt under an argon atmosphere overnight. The reaction mixture was neutralized with 25% aq NH_3_ solution to pH = 9, and the precipitate was filtered off. The precipitate was suspended in methanol, heated, and filtered out of the hot suspension to wash the product in methanol. The procedure was repeated two times. Methanol was evaporated, and the residue was purified with flash column chromatography using ethyl acetate/hexane 1:3 to 1:2 as the eluent. Yield: 549 mg (20.6%), orange crystals. MS (ESI) *m*/*z*: 322.4 ([M + H]^+^). ^1^H NMR (400 MHz, DMSO-*d*_6_): δ 1.79–2.06 (m, 4H), 3.13 (dd, *J* = 7.5, 9.4 Hz, 1H), 3.18 (s, 3H), 3.20–3.27 (m, 1H), 3.28–3.31 (m, 1H), 3.66–3.77 (m, 1H), 3.80 (s, 3H), 4.77–4.86 (m, 1H), 7.07 (d, *J* = 1.7 Hz, 1H), 7.57 (s, 2H), 7.68 (d, *J* = 1.6 Hz, 1H).

##### Methyl 4-(4-Acetoxypiperidin-1-yl)-2-aminobenzo[*d*]thiazole-6-carboxylate (**5p**)

KSCN (0.70 g, 7.18 mmol) was dissolved in acetic acid (24 mL) under an argon atmosphere, followed by the addition of Br_2_ (190 μL, 3.59 mmol). The reaction mixture was stirred for 30 min and then added dropwise to compound **4p** (0.70 g, 2.39 mmol) in acetic acid (24 mL). The reaction mixture was stirred at rt under an argon atmosphere overnight. The reaction mixture was neutralized with 2 M NaOH to pH = 9, and the precipitate was filtered off. The precipitate was suspended in methanol, heated, and filtered out of the hot suspension to wash the product in methanol. The procedure was repeated two times. Methanol was evaporated, and the residue was purified with flash column chromatography using ethyl acetate/hexane 1:2 to 1:1 as the eluent. Yield: 115 mg (13.8%), orange crystals. ^1^H NMR (400 MHz, DMSO-*d*_6_): δ 1.69–1.79 (m, 2H), 1.95–2.01 (m, 2H), 2.04 (s, 3H), 3.02–3.09 (m, 2H), 3.55–3.62 (m, 2H), 3.81 (s, 3H), 4.81–4.88 (m, 1H), 7.33 (s, 1H), 7.84 (s, 2H), 7.94 (d, *J* = 1.6 Hz, 1H).

##### Methyl 2-Amino-4-(4-(*tert*-butoxycarbonyl)piperazin-1-yl)benzo[*d*]thiazole-6-carboxylate (**5q**)

Synthesized according to general procedure D with **4q** (1.62 g, 4.83 mmol) as the reactant. 4 equiv of KSCN (1.89 g, 19.3 mmol) and 2 equiv of bromine (495 μL, 9.66 mmol) were used instead of 3 and 1.5 equiv. Instead of NH_3_ solution, the reaction mixture was neutralized with 2 M NaOH, the precipitate filtered off, washed with water, and dried. The crude product was suspended in methanol, filtered off, and then washed with ethyl acetate to get a white powder. The crude product was additionally purified with flash column chromatography using DCM/THF (10:1) as the eluent. Yield: 125 mg, (6.6%); white solid. ^1^H NMR (400 MHz, DMSO-*d*_6_): δ 1.43 (s, 9H), 3.18–3.24 (m, 4H), 3.46–3.54 (m, 4H), 3.81 (s, 3H), 7.29 (d, *J* = 1.7 Hz, 1H), 7.84 (s, 2H), 7.95 (d, *J* = 1.6 Hz, 1H). MS (ESI) *m*/*z*: 392.9 ([M + H]^+^).

##### Methyl 2-Amino-4-(1,4-dioxa-8-azaspiro[4.5]decan-8-yl)benzo[*d*]thiazole-6-carboxylate (**5r**)

KSCN (1.69 g, 17.4 mmol) was dissolved in acetic acid (12 mL) under an argon atmosphere, followed by the addition of Br_2_ (447 μL, 8.7 mmol). The reaction mixture was stirred for 1 h and then added dropwise to compound **4r** (1.27 g, 4.35 mmol) in acetic acid (10 mL). The reaction mixture was stirred at rt under an argon atmosphere overnight. The reaction mixture was neutralized with 25% aq NH_3_ solution to pH = 9, and the precipitate was filtered off. The precipitate was suspended in methanol, heated, and filtered out of the hot suspension to wash the product in methanol. The procedure was repeated three times. Methanol was evaporated, and the residue was suspended in cold methanol, filtered off, and dried to give **5o** as yellow powder. Yield 0.791 g (52.1%); yellow powder. ^1^H NMR (400 MHz, DMSO-*d*_6_): δ 1.78 (t, *J* = 5.3 Hz, 4H), 3.32 (m, 4H), 3.81 (s, 3H), 3.92 (m, 4H), 7.30 (d, *J* = 1.7 Hz, 1H), 7.81 (s, 2H), 7.91 (d, *J* = 1.6 Hz, 1H). MS (ESI) *m*/*z*: 349.9 ([M + H]^+^).

##### Methyl 2-Amino-4-(1-oxidothiomorpholino)benzo[*d*]thiazole-6-carboxylate (**5s**)

Synthesized according to general procedure D with **4s** (0.666 g, 1.97 mmol) as the reactant. Instead of NH_3_ solution, the reaction mixture was neutralized with 2 M NaOH (200 mL) to pH 5 and basified to pH 10 with saturated Na_2_CO_3_ solution. The resulting suspension was filtered to get 225 mg of the product. The filtrate was extracted with ethyl acetate (3 × 125 mL). The combined organic phases were dried over MgSO_4_, filtered, and the solvent evaporated in vacuo another 225 mg of the product which was used in the next step without further purification. MS (ESI) *m*/*z*: 326.1 ([M + H]^+^).

##### Methyl 2-Amino-4-((*tert*-butoxycarbonyl)(3-methoxypropyl)amino)benzo[*d*]thiazole-6-carboxylate (**5t**)

KSCN (3.81 g, 39.2 mmol) was dissolved in acetic acid (15 mL) under an argon atmosphere, followed by the addition of Br_2_ (1.00 mL, 19.6 mmol). The reaction mixture was stirred for 1 h and then added dropwise to compound **4t** (3.32 g, 9.81 mmol) in acetic acid (35 mL). The reaction mixture was stirred at rt under an argon atmosphere overnight. The reaction mixture was neutralized with 25% aq NH_3_ solution to pH = 9, and the precipitate was filtered off. The precipitate was suspended in methanol, heated, and filtered out of the hot suspension to wash the product in methanol. The procedure was repeated three times. Methanol was evaporated, and the residue was suspended in cold methanol, filtered off, and dried. Yield 2.04 g (52%), yellow powder. ^1^H NMR (400 MHz, DMSO-*d*_6_): δ 1.23 (s, 6H), 1.45 (s, 3H), 1.64 (m, 2H), 3.13 (s, 3H), 3.30 (m, 2H), 3.65 (m, 2H), 3.83 (s, 3H), 7.58 (d, *J* = 1.7 Hz, 1H), 8.06 (br s, 2H), 8.20 (s, 1H).

#### General Procedure E: Synthesis of Compounds **6a–k**, **6m–p**, **6r**, **6t**, and **15b–c** (with **6j** as an Example)

To a suspension of 3,4-dichloro-5-methyl-1*H*-pyrrole-2-carboxylic acid (38 mg, 0.198 mmol) in dry DCM (8 mL), oxalyl chloride (85 μL, 0.991 mmol) was added under an argon atmosphere, and the reaction mixture was stirred at rt overnight. The solvent was evaporated, and the product dried in vacuo. **5j** (55 mg, 0.198 mmol) and toluene were added, and the reaction mixture was stirred at 130 °C overnight. The reaction mixture was cooled to rt, and the precipitate was filtered off. The crude product was suspended in methanol, heated, and filtered off to give **6j** as a green solid.

##### Methyl 2-(3,4-Dichloro-5-methyl-1*H*-pyrrole-2-carboxamido)-4-morpholinobenzo[*d*]thiazole-6-carboxylate (**6a**)

Synthesized according to General procedure E with **5a** (200 mg, 0.682 mmol) as the reactant. The crude product was suspended in methanol and filtered off. Yield: 185 mg (57.8%); gray solid. ^1^H NMR (400 MHz, DMSO-*d*_6_): δ 2.29 (s, 3H), 3.37–3.43 (m, 4H), 3.82–3.86 (m, 4H), 3.87 (s, 3H), 7.42 (d, *J* = 1.6 Hz, 1H), 8.25 (d, *J* = 1.5 Hz, 1H), 11.96 (s, 1H), 12.34 (s, 1H). MS (ESI) *m*/*z*: 469.0 ([M + H]^+^).

##### Methyl 2-(3,4-Dichloro-5-methyl-1*H*-pyrrole-2-carboxamido)-4-(3-methylmorpholino)benzo[*d*]thiazole-6-carboxylate (**6b**)

Synthesized according to general procedure E with **5b** (60 mg, 0.195 mmol) as the reactant. The crude product was suspended in methanol and filtered off. Yield: 66 mg (65.7%); gray solid. ^1^H NMR (400 MHz, DMSO-*d*_6_): δ 0.91 (d, *J* = 6.6 Hz, 3H), 2.28 (s, 3H), 3.05 (d, *J* = 11.9 Hz, 1H), 3.37–3.46 (m, 1H), 3.58–3.76 (m, 2H), 3.83–3.98 (m, 5H), 4.61 (s, 1H), 7.42 (d, *J* = 1.7 Hz, 1H), 8.26 (d, *J* = 1.6 Hz, 1H), 11.96 (s, 1H), 12.33 (s, 1H). MS (ESI) *m*/*z*: 482.9 ([M + H]^+^).

##### Methyl (*R*)-2-(3,4-Dichloro-5-methyl-1*H*-pyrrole-2-carboxamido)-4-(3-methylmorpholino)benzo[*d*]thiazole-6-carboxylate (**6c**)

Synthesized according to general procedure E with **5c** (80 mg, 0.26 mmol) as the reactant. The crude product was purified with flash column chromatography using DCM/methanol/NH_4_OH 30:1:0.1 as the eluent to give **6c** as a gray solid. Yield: 30 mg (23.9%); gray solid. ^1^H NMR (400 MHz, DMSO-*d*_6_): δ 0.92 (d, *J* = 6.6 Hz, 3H), 2.29 (s, 3H), 2.99–3.08 (m, 1H), 3.60–3.66 (m, 2H), 3.67–3.75 (m, 2H), 3.86–3.98 (m, 6H), 4.59–4.67 (m, 1H), 7.43 (s, 1H), 8.27 (s, 1H), 12.00 (s, 1H), 12.35 (s, 1H). MS (ESI) *m*/*z*: 482.9 ([M + H]^+^).

##### Methyl (*S*)-2-(3,4-Dichloro-5-methyl-1*H*-pyrrole-2-carboxamido)-4-(3-methylmorpholino)benzo[*d*]thiazole-6-carboxylate (**6d**)

Synthesized according to general procedure E with **5d** (300 mg, 0.97 mmol) as the reactant and stirring the reaction mixture in toluene for 2 days. After 2 days, additional 0.5 equiv of 3,4-dichloro-5-methyl-1*H*-pyrrole-2-carbonyl chloride was added, and the reaction mixture was stirred overnight. Yield: 297 mg (63.0%); gray solid. ^1^H NMR (400 MHz, DMSO-*d*_6_): δ 0.91 (d, *J* = 6.5 Hz, 3H), 2.98–3.01 (m, 1H), 3.56–3.65 (m, 2H), 3.66–3.73 (m, 2H), 3.86 (s, 3H), 3.87–3.98 (m, 3H), 4.56–4.67 (m, 1H), 7.41 (s, 1H), 8.26 (s, 1H), 11.98 (s, 1H), 12.35 (s, 1H). MS (ESI) *m*/*z*: 482.8 ([M + H]^+^).

##### Methyl 2-(3,4-Dichloro-5-methyl-1*H*-pyrrole-2-carboxamido)-4-(2-methylmorpholino)benzo[*d*]thiazole-6-carboxylate (**6e**)

Synthesized according to general procedure E with **6e** (90 mg, 0.293 mmol) as the reactant. The crude product was suspended in methanol and filtered off. Yield: 85 mg (60.1%); light gray solid. ^1^H NMR (400 MHz, DMSO-*d*_6_): δ 1.19 (d, *J* = 6.2 Hz, 3H), 2.29 (s, 3H), 2.78 (td, *J* = 3.1, 11.6 Hz, 1H), 3.74–3.84 (m, 3H), 3.85–3.87 (m, 4H), 3.92–4.04 (m, 2H), 7.41 (d, *J* = 1.6 Hz, 1H), 8.25 (d, *J* = 1.5 Hz, 1H), 11.96 (s, 1H), 12.35 (s, 1H). MS (ESI) *m*/*z*: 481.0 ([M – H]^−^).

##### Methyl 2-(3,4-Dichloro-5-methyl-1*H*-pyrrole-2-carboxamido)-4-(2,6-dimethylmorpholino)benzo[*d*]thiazole-6-carboxylate (**6f**)

Synthesized according to general procedure E with **5f** (280 mg, 0.871 mmol) as the reactant. The reaction mixture was cooled to rt, and the precipitate was filtered off, washed with methanol, and dried. Yield: 152 mg (35.1%); light gray solid. ^1^H NMR (400 MHz, DMSO-*d*_6_): δ 1.19 (d, *J* = 6.2 Hz, 6H), 2.29 (s, 3H), 2.41 (t, *J* = 10.8 Hz, 2H), 3.76–3.86 (m, 2H), 3.87 (s, 3H), 3.94 (d, *J* = 11.2 Hz, 2H), 7.40 (d, *J* = 1.6 Hz, 1H), 8.25 (d, *J* = 1.5 Hz, 1H), 11.96 (s, 1H), 12.34 (s, 1H). MS (ESI) *m*/*z*: 496.8 ([M + H]^+^).

##### Methyl 4-((1*R*,5*S*)-8-Oxa-3-azabicyclo[3.2.1]octan-3-yl)-2-(3,4-dichloro-5-methyl-1*H*-pyrrole-2-carboxamido)benzo[*d*]thiazole-6-carboxylate (**6g**)

Synthesized according to general procedure E with **5g** (300 mg, 0.939 mmol) as the reactant. The reaction mixture was cooled to rt, and the precipitate was filtered off, washed with methanol, and dried. Yield: 125 mg (26.9%); light gray solid. ^1^H NMR (400 MHz, DMSO-*d*_6_): δ 1.89 (s, 2H), 2.12 (t, *J* = 6.9 Hz, 2H), 2.30 (s, 3H), 2.98 (d, *J* = 11.0 Hz, 2H), 3.86 (s, 3H), 3.93 (t, *J* = 13.8 Hz, 2H), 4.45 (s, 2H), 7.30 (s, 1H), 8.18 (d, *J* = 1.4 Hz, 1H), 11.85 (s, 1H), 12.38 (s, 1H). HRMS (ESI^+^) *m*/*z*: 495.0650 [M + H]^+^ (calcd *m*/*z*: 495.0655 for C_21_H_21_Cl_2_N_4_O_4_S).

##### Methyl 4-((1*R*,5*S*)-3-Oxa-8-azabicyclo[3.2.1]octan-8-yl)-2-(3,4-dichloro-5-methyl-1*H*-pyrrole-2-carboxamido)benzo[*d*]thiazole-6-carboxylate (**6h**)

Synthesized according to general procedure E with **5h** (228 mg, 0.71 mmol) as the reactant. Yield: 211 mg (60%), gray solid. ^1^H NMR (400 MHz, DMSO-*d*_6_): δ 1.90–2.05 (m, 4H), 2.28 (s, 3H), 3.56 (d, *J* = 10.8 Hz, 2H), 3.80 (d, *J* = 10.8 Hz, 2H), 3.86 (s, 3H), 4.84 (s, 2H), 7.34 (d, *J* = 1.5 Hz, 1H), 8.06 (d, *J* = 1.5 Hz, 1H), 11.83 (s, 1H), 12.35 (s, 1H). MS (ESI) *m*/*z*: 494.8 ([M – H]^−^).

##### Methyl 2-(3,4-Dichloro-5-methyl-1*H*-pyrrole-2-carboxamido)-4-(2-(trifluoromethyl)morpholino)benzo[*d*]thiazole-6-carboxylate (**6i**)

Synthesized according to general procedure E with **5i** (72 mg, 0.199 mmol) as the reactant. The crude product was suspended in methanol, filtered off, and dried. Yield: 65 mg (60.1%); gray solid. ^1^H NMR (400 MHz, DMSO-*d*_6_): δ 2.29 (s, 3H), 2.91–3.00 (m, 2H), 3.89 (s, 3H), 3.90–4.03 (m, 2H), 4.08 (d, *J* = 11.1 Hz, 1H), 4.12–4.20 (m, 1H), 4.44 (s, 1H), 7.47 (d, *J* = 1.6 Hz, 1H), 8.32 (d, *J* = 1.5 Hz, 1H), 11.96 (s, 1H), 12.37 (s, 1H). MS (ESI) *m*/*z*: 534.8 ([M – H]^−^).

##### Methyl 2-(3,4-Dichloro-5-methyl-1*H*-pyrrole-2-carboxamido)-4-(pyrrolidin-1-yl)benzo[*d*]thiazole-6-carboxylate (**6j**)

Yield: 80 mg (89.0%); green solid. ^1^H NMR (400 MHz, DMSO-*d*_6_): δ 1.90–2.04 (m, 4H), 2.28 (s, 3H), 3.68–3.71 (m, 4H), 3.85 (s, 3H), 7.07 (d, *J* = 1.7 Hz, 1H), 7.89 (d, *J* = 1.6 Hz, 1H), 11.66 (s, 1H), 12.36 (s, 1H). HRMS (ESI^+^) *m*/*z*: 453.0543 [M + H]^+^ (calcd *m*/*z*: 453.0549 for C_19_H_19_Cl_2_N_4_O_3_S).

##### Methyl (*S*)-4-(3-((*tert*-Butoxycarbonyl)amino)pyrrolidin-1-yl)-2-(3,4-dichloro-5-methyl-1*H*-pyrrole-2-carboxamido)benzo[*d*]thiazole-6-carboxylate (**6k**)

Synthesized according to general procedure E. Additional details on the experimental procedure, yield, and analytical data for **6k** were previously described in the authors’ patent application.^42^

#### General Procedure F: Synthesis of Compounds **6l**, **6q**, **6s**, **9a**, **13**, and **15a** (with **6l** as an Example)

Compound **5l** (71 mg, 0.222 mmol) was suspended in DMF (4 mL) and heated to 60 °C upon which it dissolved completely. Na_2_CO_3_ (24 mg, 0.222 mmol) was added, 2,2,2-trichloro-1-(3,4-dichloro-5-methyl-1*H*-pyrrol-2-yl)ethan-1-one (66 mg, 0.222 mmol) dissolved in 1 mL of DMF was added dropwise, and the reaction mixture was stirred at 60 °C overnight. The solvent was evaporated in vacuo, ethyl acetate (10 mL) and water (10 mL) were added, and the phases were separated. The organic phase was dried over Na_2_SO_4_, filtered, and the solvent was evaporated in vacuo. The crude product was suspended in methanol and filtered off.

##### Methyl (*S*)-2-(3,4-Dichloro-5-methyl-1*H*-pyrrole-2-carboxamido)-4-(3-(dimethylamino)pyrrolidin-1-yl)benzo[*d*]thiazole-6-carboxylate (**6l**)

Yield: 66 mg (60.0%); black solid. ^1^H NMR (400 MHz, DMSO-*d*_6_): δ 1.93–2.13 (m, 1H), 2.29 (s, 3H), 3.56 (d, *J* = 8.3 Hz, 6H), 3.66 (d, *J* = 8.5 Hz, 1H), 3.77–3.85 (m, 2H), 3.86 (s, 3H), 3.91–3.99 (m, 2H), 4.44–4.64 (m, 1H), 7.10 (s, 1H), 8.02 (s, 1H), 11.89 (s, 1H), 12.81 (s, 1H). MS (ESI) *m*/*z*: 494.0 ([M – H]^−^).

##### Methyl 2-(3,4-Dichloro-5-methyl-1*H*-pyrrole-2-carboxamido)-4-(2-oxooxazolidin-3-yl)benzo[*d*]thiazole-6-carboxylate (**6m**)

Synthesized according to general procedure E with **5m** (55 mg, 0.188 mmol) as the reactant. The reaction mixture was cooled to rt, and the precipitate was filtered off, washed with methanol, and dried. Yield: 62 mg (70.5%); light gray solid. ^1^H NMR (400 MHz, DMSO-*d*_6_): δ 2.29 (s, 3H), 3.90 (s, 3H), 4.31 (t, *J* = 8.0 Hz, 2H), 4.54 (dd, *J* = 6.8, 9.2 Hz, 2H), 8.14 (d, *J* = 1.6 Hz, 1H), 8.63 (d, *J* = 1.7 Hz, 1H), 12.15 (s, 1H), 12.38 (s, 1H). HRMS (ESI^+^) *m*/*z*: 469.0132 [M + H]^+^ (calcd *m*/*z*: 469.0135 for C_18_H_15_Cl_2_N_4_O_5_S).

##### Methyl 2-(3,4-Dichloro-5-methyl-1*H*-pyrrole-2-carboxamido)-4-(2-(methoxymethyl)pyrrolidin-1-yl)benzo[*d*]thiazole-6-carboxylate (**6n**)

Synthesized according to general procedure E with **5n** (400 mg, 1.24 mmol) as the reactant and stirring the reaction mixture for 48 h. Additional 0.5 equiv of 3,4-dichloro-5-methyl-1*H*-pyrrole-2-carbonyl chloride was added, and the reaction mixture was stirred at 130 °C overnight to get **6n** (523 mg; 84.5%) as a gray solid. The product was used in the next step without further purification.

##### Methyl (*S*)-2-(3,4-Dichloro-5-methyl-1*H*-pyrrole-2-carboxamido)-4-(2-(methoxymethyl)pyrrolidin-1-yl)benzo[*d*]thiazole-6-carboxylate (**6o**)

Synthesized according to general procedure E with **5o** (540 mg, 1.70 mmol) as the reactant. Additional 0.9 equiv of 3,4-dichloro-5-methyl-1*H*-pyrrole-2-carbonyl chloride was added, and the reaction mixture was stirred at 130 °C overnight. Yield: 0.76 g (90.9%); gray solid. ^1^H NMR (400 MHz, DMSO-*d*_6_): δ 1.89–2.11 (m, 4H), 2.28 (s, 3H), 2.44–2.46 (m, 1H), 2.54–2.57 (m, 1H), 3.22 (s, 3H), 3.36–3.41 (m, 2H), 3.85 (s, 3H), 4.86–4.95 (m, 1H), 7.17 (d, *J* = 1.6 Hz, 1H), 7.93 (d, *J* = 1.5 Hz, 1H), 11.55 (s, 1H), 12.41 (s, 1H). MS (ESI) *m*/*z*: 497.5 ([M + H]^+^).

##### Methyl 4-(4-Acetoxypiperidin-1-yl)-2-(3,4-dichloro-5-methyl-1*H*-pyrrole-2-carboxamido)benzo[*d*]thiazole-6-carboxylate (**6p**)

Synthesized according to general procedure E with **5p** (120 mg, 0.35 mmol) as the reactant. Additional 0.5 equiv of 3,4-dichloro-5-methyl-1*H*-pyrrole-2-carbonyl chloride was added, and the reaction mixture was stirred at 130 °C overnight. Yield: 150 mg (83.1%); gray solid. ^1^H NMR (400 MHz, DMSO-*d*_6_): δ 1.75–1.85 (m, 2H), 2.00–2.10 (m, 5H), 2.28 (s, 3H), 3.64–3.75 (m, 2H), 3.87 (s, 3H), 4.04–4.16 (m, 2H), 4.86–4.93 (m, 1H), 7.45 (s, 1H), 8.23 (s, 1H), 11.91 (s, 1H), 12.39 (s, 1H).

##### Methyl 4-(4-(*tert*-Butoxycarbonyl)piperazin-1-yl)-2-(3,4-dichloro-5-methyl-1*H*-pyrrole-2-carboxamido)benzo[*d*]thiazole-6-carboxylate (**6q**)

Synthesized according to general procedure F with **5q** (118 mg, 0.301 mmol) as the reactant. After the addition of ethyl acetate (3 mL) and water (3 mL), the product precipitated and filtered off. The crude product was washed with MeOH to get the title compound (24 mg, 14% yield) which was used in the next step without further purification. MS (ESI) *m*/*z*: 566.1 ([M – H]^−^).

##### Methyl 2-(3,4-Dichloro-5-methyl-1*H*-pyrrole-2-carboxamido)-4-(1,4-dioxa-8-azaspiro[4.5]decan-8-yl)benzo[*d*]thiazole-6-carboxylate (**6r**)

Synthesized according to general procedure E with **5r** (774 mg, 2.22 mmol) as the reactant. Yield: 517 mg (44.4%); brown solid. The crude product was used in the next step without further purification.

##### Methyl 2-(3,4-Dichloro-5-methyl-1*H*-pyrrole-2-carboxamido)-4-(1-oxidothiomorpholino)benzo[*d*]thiazole-6-carboxylate (**6s**)

Synthesized according to general procedure F with **5s** (440 mg, 1.35 mmol) as the reactant and heating the reaction mixture at 45 °C. Instead of extraction, the residue was suspended in water (3 mL) and acidified to pH 2–3 with 10% citric acid. The suspension was stirred for 30 min and filtered over glass filter. The residue was triturated with water and methanol and dried to give 158 mg (65%) of the product as a brown solid, which was used in the next step without further purification. MS (ESI) *m*/*z*: 501.2 ([M + H]^+^).

##### Methyl 4-((*tert*-Butoxycarbonyl)(3-methoxypropyl)amino)-2-(3,4-dichloro-5-methyl-1*H*-pyrrole-2-carboxamido)benzo[*d*]thiazole-6-carboxylate (**6t**)

Synthesized according to general procedure E with **5t** (791 mg, 2.00 mmol) as the reactant. The product was additionally washed with hot acetonitrile. Yield: 309 mg; brown solid. A part (19%) of the product was in the form without the boc-protecting group; however, this did not prove to be problematic, and future reactions were carried out with this mixture without difficulty. ^1^H NMR (400 MHz, DMSO-*d*_6_): δ 1.23 (s, 6H), 1.47 (br s, 3H), 1.70 (m, 2H), 2.28 (s, 3H), 3.14 (s, 3H), 3.33 (m, 2H), 3.76 (br s, 2H), 3.89 (s, 3H), 7.79 (d, *J* = 1.7 Hz, 1H), 8.59 (s, 1H), 12.11 (br s, 1H), 12.36 (br s, 1H). MS (ESI) *m*/*z*: 570.8 ([M + H]^+^).

#### General Procedure G: Synthesis of Compounds **7a–t**, **14**, and **16a–c** (with **7a** as an Example)

Compound **6a** (160 mg, 0.341 mmol) was dissolved in methanol (15 mL), 1 M NaOH (1.71 mL, 1.71 mmol) was added, and the reaction mixture was stirred at 40 °C for 72 h. The solvent was evaporated, and the residue was acidified with 1 M HCl to pH = 1. The precipitate was filtered off, washed with water, the crude product resuspended in methanol, filtered off, and dried to afford **7a** as a beige solid.

##### 2-(3,4-Dichloro-5-methyl-1*H*-pyrrole-2-carboxamido)-4-morpholinobenzo[*d*]thiazole-6-carboxylic Acid (**7a**)

Yield: 120 mg (77.3%); beige solid. ^1^H NMR (400 MHz, DMSO-*d*_6_): δ 2.29 (s, 3H), 3.38–3.42 (m, 5H), 3.79–3.93 (m, 4H), 7.43 (d, *J* = 1.6 Hz, 1H), 8.22 (d, *J* = 1.5 Hz, 1H), 11.93 (s, 1H), 12.34 (s, 1H), 12.90 (s, 1H). ^13^C{^1^H} NMR (101 MHz, DMSO-*d*_6_): δ 11.50, 50.69, 66.77, 110.45, 113.54, 116.12, 116.87, 117.28, 126.98, 130.34, 133.40, 143.84, 144.16, 157.09, 158.69, 167.77. HRMS (ESI^+^) *m*/*z*: 455.0338 [M + H]^+^ (calcd *m*/*z*: 455.0342 for C_18_H_17_Cl_2_N_4_O_4_S). HPLC: *t*_r_ 6.41 min (98.5% at 254 nm).

##### 2-(3,4-Dichloro-5-methyl-1*H*-pyrrole-2-carboxamido)-4-(3-methylmorpholino)benzo[*d*]thiazole-6-carboxylic Acid (**7b**)

Synthesized according to general procedure G with **6b** (55 mg, 0.114 mmol) as the reactant. The reaction mixture was stirred at 50 °C for 48 h. Instead of methanol, the crude product was resuspended in ethyl acetate, filtered off, and dried. Yield: 37 mg (69.3%); gray solid. ^1^H NMR (400 MHz, DMSO-*d*_6_): δ 0.92 (d, *J* = 6.6 Hz, 3H), 2.29 (s, 3H), 3.06 (s, 1H), 3.73 (s, 2H), 3.94 (s, 3H), 4.59 (s, 1H), 7.46 (s, 1H), 8.25 (s, 1H), 11.98 (s, 1H), 12.38 (s, 1H), signal for COOH group is not seen. HRMS (ESI^+^) *m*/*z*: 469.0495 [M + H]^+^ (calcd *m*/*z*: 469.0499 for C_19_H_19_Cl_2_N_4_O_4_S). HPLC: *t*_r_ 6.48 min (97.0% at 254 nm).

##### (*R*)-2-(3,4-Dichloro-5-methyl-1*H*-pyrrole-2-carboxamido)-4-(3-methylmorpholino)benzo[*d*]thiazole-6-carboxylic Acid (**7c**)

Synthesized according to general procedure G with **6c** (30 mg, 0.064 mmol) as the reactant. 5 equiv of 2 M NaOH was used, and after 3 days, additional 7 equiv of 2 M NaOH was added, and the reaction mixture was stirred for 4 more days. Yield: 7 mg (24.0%); gray solid. [α]_D_^25^ + 51.6 (*c* 0.202, DMF). ^1^H NMR (400 MHz, DMSO-*d*_6_): δ 0.93 (d, *J* = 6.5 Hz, 3H), 2.29 (s, 3H), 3.77 (s, 3H), 3.97 (s, 3H), 4.63 (s, 1H), 7.51 (s, 1H), 8.32 (s, 1H), 12.09 (s, 1H), 12.44 (s, 1H), signal for COOH group is not seen. HRMS (ESI^+^) *m*/*z*: 469.0494 [M + H]^+^ (calcd *m*/*z*: 469.0499 for C_19_H_19_Cl_2_N_4_O_4_S). HPLC: *t*_r_ 6.46 min (96.8% at 254 nm).

##### (*S*)-2-(3,4-Dichloro-5-methyl-1*H*-pyrrole-2-carboxamido)-4-(3-methylmorpholino)benzo[*d*]thiazole-6-carboxylic Acid (**7d**)

Synthesized according to general procedure G with **6d** (297 mg, 0.61 mmol) as the reactant. 5 equiv of 2 M NaOH was used, and after 2 days, additional 5 equiv of 2 M NaOH was added, and the reaction mixture was stirred for 3 more days. Yield: 168 mg (58.3%); gray solid. [α]_D_^25^ – 117.7 (*c* 0.026, DMF). ^1^H NMR (400 MHz, DMSO-*d*_6_): δ 0.91 (d, *J* = 6.6 Hz, 3H), 2.28 (s, 3H), 2.98–3.08 (m, 1H), 3.36–3.46 (m, 2H), 3.62 (dd, *J* = 11.2, 3.5 Hz, 1H), 3.66–3.75 (m, 1H), 3.87–3.97 (m, 1H), 4.58 (br s, 1H), 7.43 (s, 1H), 8.22 (d, *J* = 1.5 Hz, 1H), 11.95 (s, 1H), 12.34 (s, 1H), 12.91 (s, 1H). HRMS (ESI^+^) *m*/*z*: 469.0492 [M + H]^+^ (calcd *m*/*z*: 469.0499 for C_19_H_19_Cl_2_N_4_O_4_S). HPLC: *t*_r_ 6.49 min (98.3% at 254 nm).

##### 2-(3,4-Dichloro-5-methyl-1*H*-pyrrole-2-carboxamido)-4-(2-methylmorpholino)benzo[*d*]thiazole-6-carboxylic Acid (**7e**)

Synthesized according to general procedure G with **6e** (73 mg, 0.151 mmol) as the reactant. 10 equiv of 1 M NaOH (1.51 mL, 1.51 mmol) was used, and the reaction mixture was stirred at 50 °C. Instead of methanol, the crude product was resuspended in ethyl acetate, filtered off, and dried. Yield: 60 mg (84.7%); gray solid. ^1^H NMR (400 MHz, DMSO-*d*_6_): δ 1.20 (d, *J* = 6.2 Hz, 3H), 2.29 (s, 3H), 2.51–2.59 (m, 1H), 2.72–2.84 (m, 1H), 3.74–3.90 (m, 3H), 3.91–4.05 (m, 2H), 7.42 (d, *J* = 1.6 Hz, 1H), 8.21 (d, *J* = 1.5 Hz, 1H), 11.96 (s, 1H), 12.41 (s, 1H), 12.83 (s, 1H). ^13^C{^1^H} NMR (101 MHz, DMSO-*d*_6_): δ 10.92, 18.77, 49.60, 55.95, 65.78, 71.04, 109.90, 113.35, 115.76, 116.62, 126.37, 129.70, 132.92, 142.80, 143.28, 156.51, 158.23, 162.92, 167.15. HRMS (ESI^+^) *m*/*z*: 469.0500 [M + H]^+^ (calcd *m*/*z*: 469.0499 for C_19_H_19_Cl_2_N_4_O_4_S). HPLC: *t*_r_ 6.69 min (97.9% at 254 nm).

##### 2-(3,4-Dichloro-5-methyl-1*H*-pyrrole-2-carboxamido)-4-(2,6-dimethylmorpholino)benzo[*d*]thiazole-6-carboxylic Acid (**7f**)

Synthesized according to general procedure G with **6f** (100 mg, 0.201 mmol) as the reactant. After 24 h, additional 5 equiv of 1 M NaOH (1.01 mL) was added, and the reaction mixture was stirred at 40 °C for another 72 h. Instead of methanol, the crude product was resuspended in diethyl ether, filtered off, and dried. Yield: 76 mg (78.2%); beige solid. ^1^H NMR (400 MHz, DMSO-*d*_6_): δ 1.19 (d, *J* = 6.2 Hz, 6H), 2.29 (s, 3H), 2.41 (t, *J* = 10.8 Hz, 2H), 3.80–3.91 (m, 2H), 3.95 (d, *J* = 11.2 Hz, 2H), 7.41 (d, *J* = 1.6 Hz, 1H), 8.20 (d, *J* = 1.5 Hz, 1H), 12.00 (s, 1H), 12.46 (s, 1H), 12.93 (br s, 1H). ^13^C{^1^H} NMR (101 MHz, DMSO-*d*_6_): δ 11.47, 19.33, 55.92, 71.52, 110.48, 113.74, 116.40, 116.87, 117.16, 126.94, 130.21, 133.45, 143.44, 143.80, 157.05, 158.63, 167.76. HRMS (ESI^+^) *m*/*z*: 483.0653 [M + H]^+^ (calcd *m*/*z*: 483.0655 for C_20_H_21_Cl_2_N_4_O_4_S). HPLC: *t*_r_ 7.01 min (97.3% at 254 nm).

##### 4-((1*R*,5*S*)-8-Oxa-3-azabicyclo[3.2.1]octan-3-yl)-2-(3,4-dichloro-5-methyl-1*H*-pyrrole-2-carboxamido)benzo[*d*]thiazole-6-carboxylic Acid (**7g**)

Synthesized according to general procedure G with **6g** (100 mg, 0.202 mmol) as the reactant. 10 equiv of 1 M NaOH (2.02 mL, 2.02 mmol) was used, and after 24 h, additional 5 equiv of 1 M NaOH (1.01 mL, 1.01 mmol) was added, and the reaction mixture was stirred at 40 °C for another 72 h. Yield: 57 mg (58.7%); beige solid. [α]_D_^25^ + 63.31 (*c* 0.046, DMF). ^1^H NMR (400 MHz, DMSO-*d*_6_): δ 1.82–1.95 (m, 2H), 2.12 (d, *J* = 6.7 Hz, 2H), 2.29 (s, 3H), 2.97 (d, *J* = 10.1 Hz, 2H), 3.91 (d, *J* = 11.1 Hz, 2H), 4.39–4.51 (m, 2H), 7.31 (d, *J* = 1.6 Hz, 1H), 8.14 (d, *J* = 1.5 Hz, 1H), 11.79 (s, 1H), 12.38 (s, 1H), 12.84 (s, 1H). ^13^C{^1^H} NMR (101 MHz, DMSO-*d*_6_): δ 11.46, 28.45, 55.31, 74.11, 110.46, 112.97, 115.86, 116.06, 117.23, 126.97, 130.40, 133.45, 143.33, 144.19, 157.06, 158.09, 167.86. HRMS (ESI^+^) *m*/*z*: 481.0481 [M + H]^+^ (calcd *m*/*z*: 481.0499 for C_20_H_19_Cl_2_N_4_O_4_S). HPLC: *t*_r_ 6.99 min (99.6% at 254 nm).

##### 4-((1*R*,5*S*)-3-Oxa-8-azabicyclo[3.2.1]octan-8-yl)-2-(3,4-dichloro-5-methyl-1*H*-pyrrole-2-carboxamido)benzo[*d*]thiazole-6-carboxylic Acid (**7h**)

Synthesized according to general procedure G with **6h** (130 mg, 0.26 mmol) as the reactant. 10 equiv of 1 M NaOH (2.6 mL, 2.6 mmol) was used, and after 24 h, additional 5 equiv of 1 M NaOH (1.3 mL, 1.3 mmol) was added and left to stir for another 24 h. Yield: 170 mg (88%), gray solid. [α]_D_^25^ + 54.6 (*c* 0.046, DMF). ^1^H NMR (400 MHz, DMSO-*d*_6_): δ 1.87–2.04 (m, 4H), 2.28 (s, 3H), 3.55 (d, *J* = 10.8 Hz, 2H), 3.81 (d, *J* = 10.8 Hz, 2H), 4.82 (s, 2H), 7.34 (s, 1H), 8.02 (s, 1H), 11.78 (s, 1H), 12.34 (s, 1H), 12.84 (s, 1H). ^13^C{^1^H} NMR (101 MHz, DMSO-*d*_6_): δ 11.50, 26.75, 49.06, 57.85, 70.21, 110.38, 112.74, 114.03, 115.94, 117.32, 127.33, 130.22, 133.86, 140.22, 141.76, 156.99, 157.37, 167.89. HRMS (ESI^+^) *m*/*z*: 481.0497 [M + H]^+^ (calcd *m*/*z*: 481.0499 for C_20_H_19_Cl_2_N_4_O_4_S). HPLC: *t*_r_ 6.82 min (97.7% at 254 nm).

##### 2-(3,4-Dichloro-5-methyl-1*H*-pyrrole-2-carboxamido)-4-(2-(trifluoromethyl)morpholino)benzo[*d*]thiazole-6-carboxylic Acid (**7i**)

Synthesized according to general procedure G with **6i** (58 mg, 0.108 mmol) as the reactant. The reaction mixture was stirred at 50 °C. Instead of methanol, the crude product was resuspended in acetonitrile, filtered off, and dried. Yield: 32 mg (56.6%); purple solid. ^1^H NMR (400 MHz, DMSO-*d*_6_): δ 2.29 (s, 3H), 2.90–2.99 (m, 2H), 3.88–4.11 (m, 3H), 4.12–4.21 (m, 1H), 4.40–4.48 (m, 1H), 7.47 (d, *J* = 1.5 Hz, 1H), 8.28 (d, *J* = 1.5 Hz, 1H), 11.95 (s, 1H), 12.41 (s, 1H), 12.96 (s, 1H). ^13^C{^1^H} NMR (101 MHz, DMSO-*d*_6_): δ 10.93, 47.70, 49.24, 66.17, 71.93, 72.23, 72.53, 109.89, 113.59, 115.57, 116.70, 117.10, 122.18, 124.97, 126.42, 129.81, 132.91, 142.57, 143.43, 156.58, 158.60, 167.09. HRMS (ESI^–^) *m*/*z*: 521.0068 [M – H]^−^ (calcd *m*/*z*: 521.0070 for C_19_H_14_Cl_2_F_3_N_4_O_4_S). HPLC: *t*_r_ 7.29 min (97.3% at 254 nm).

##### 2-(3,4-Dichloro-5-methyl-1*H*-pyrrole-2-carboxamido)-4-(pyrrolidin-1-yl)benzo[*d*]thiazole-6-carboxylic Acid (**7j**)

Synthesized according to general procedure G. HPLC: *t*_r_ 7.65 min (92.2% at 254 nm). Additional details on the experimental procedure, yield, and analytical data for **7j** were previously described in the authors’ patent application.^42^

##### (*S*)-4-(3-((*tert*-Butoxycarbonyl)amino)pyrrolidin-1-yl)-2-(3,4-dichloro-5-methyl-1*H*-pyrrole-2-carboxamido)benzo[*d*]thiazole-6-carboxylic Acid (**7k**)

Synthesized according to general procedure G. HPLC: *t*_r_ 7.39 min (97.0% at 254 nm). Additional details on the experimental procedure, yield, and analytical data for **7k** were previously described in the authors’ patent application.^42^ HPLC: *t*_r_ 7.39 min (97.0% at 254 nm).

##### (*S*)-2-(3,4-Dichloro-5-methyl-1*H*-pyrrole-2-carboxamido)-4-(3-(dimethylamino)pyrrolidin-1-yl)benzo[*d*]thiazole-6-carboxylic Acid (**7l**)

Synthesized according to general procedure G with **6l** (55 mg, 0.111 mmol) as the reactant. 10 equiv of 1 M NaOH was used instead of 5. The residue was acidified with 1 M HCl to pH = 3. Instead of methanol, the precipitate was resuspended in ethyl acetate, filtered off, and dried. Yield: 15 mg (28.0%); brown solid. [α]_D_^25^ + 71.9 (*c* 0.014, DMF). ^1^H NMR (400 MHz, DMSO-*d*_6_): δ 2.18–2.28 (m, 1H), 2.29 (s, 3H), 2.92 (dd, *J* = 4.8, 18.2 Hz, 6H), 3.87 (s, 2H), 3.98 (q, *J* = 6.8, 7.8 Hz, 2H), 4.18 (d, *J* = 5.4 Hz, 1H), 4.47–4.68 (m, 1H), 7.20 (dd, *J* = 1.6, 24.9 Hz, 1H), 8.03 (dd, *J* = 1.5, 23.6 Hz, 1H), 10.41 (s, 1H), 12.11 (d, *J* = 6.3 Hz, 1H), 12.99 (d, *J* = 15.3 Hz, 1H). HRMS (ESI^+^) *m*/*z*: 482.0809 [M + H]^+^ (calcd *m*/*z*: 482.0815 for C_20_H_22_Cl_2_N_5_O_3_S). HPLC: *t*_r_ 4.84 min (96.2% at 254 nm).

##### 2-(3,4-Dichloro-5-methyl-1*H*-pyrrole-2-carboxamido)-4-(2-oxooxazolidin-3-yl)benzo[*d*]thiazole-6-carboxylic Acid (**7m**)

Synthesized according to general procedure G with **6m** (50 mg, 0.107 mmol) as the reactant. Yield: 30 mg, (61.9%); white solid. ^1^H NMR (400 MHz, DMSO-*d*_6_): δ 2.29 (s, 3H), 4.30 (t, *J* = 8.0 Hz, 2H), 4.49–4.59 (m, 2H), 8.11 (d, *J* = 1.7 Hz, 1H), 8.59 (d, *J* = 1.6 Hz, 1H), 12.12 (s, 1H), 12.38 (s, 1H), 13.12 (s, 1H). ^13^C{^1^H} NMR (101 MHz, DMSO-*d*_6_): δ 11.41, 47.62, 63.14, 110.63, 116.32, 117.07, 122.61, 124.69, 126.48, 129.49, 131.02, 132.60, 133.51, 151.95, 157.06, 161.58, 167.14, 170.27. HRMS (ESI^+^) *m*/*z*: 454.9978 [M + H]^+^ (calcd *m*/*z*: 454.9978 for C_18_H_13_Cl_2_N_4_O_5_S). HPLC: *t*_r_ 5.95 min (96.4% at 254 nm).

##### 2-(3,4-Dichloro-5-methyl-1*H*-pyrrole-2-carboxamido)-4-(2-(methoxymethyl)pyrrolidin-1-yl)benzo[*d*]thiazole-6-carboxylic Acid (**7n**)

Synthesized according to general procedure G with **6n** (520 mg, 1.05 mmol) as the reactant and using 2 M NaOH. The reation mixture was stirred for 2 days. Additional 5 equiv of 2 M NaOH was added every 2 days. The reaction mixture was stirred at 40 °C for 6 days in total. Yield: 83 mg (16.4%); gray solid. ^1^H NMR (400 MHz, DMSO-*d*_6_): δ 1.90–2.22 (m, 4H), 2.29 (s, 3H), 3.20 (s, 3H), 3.24–3.34 (m, 1H), 3.42–3.57 (m, 2H), 3.84–3.96 (m, 1H), 4.80–4.91 (m, 1H), 7.42 (s, 1H), 8.05 (s, 1H), 11.83 (s, 1H), 12.73 (s, 1H), signal for COOH group is not seen. HRMS (ESI^+^) *m*/*z*: 483.0652 [M + H]^+^ (calcd *m*/*z*: 483.0655 for C_20_H_21_Cl_2_N_4_O_4_S). HPLC: *t*_r_ 7.50 min (97.7% at 254 nm).

##### (*S*)-2-(3,4-Dichloro-5-methyl-1*H*-pyrrole-2-carboxamido)-4-(2-(methoxymethyl)pyrrolidin-1-yl)benzo[*d*]thiazole-6-carboxylic Acid (**7o**)

Synthesized according to general procedure G with **6o** (0.76 g, 1.52 mmol) as the reactant and using 2 M NaOH. The reaction mixture was stirred for 2 days. Additional 5 equiv of 2 M NaOH was added, and the reaction mixture was stirred at 40 °C overnight. Yield: 353 mg (47.8%); gray solid. [α]_D_^25^ – 69.4 (*c* 0.036, DMF). ^1^H NMR (400 MHz, DMSO-*d*_6_): δ 1.89–2.26 (m, 4H), 2.29 (s, 3H), 3.19 (s, 3H), 3.27–3.39 (m, 1H), 3.54 (s, 2H), 3.84–3.94 (m, 1H), 4.78–4.90 (m, 1H), 7.54 (s, 1H), 8.12 (s, 1H), 12.05 (s, 1H), 12.97 (s, 1H), signal for COOH group is not seen. HRMS (ESI^+^) *m*/*z*: 483.0649 [M + H]^+^ (calcd *m*/*z*: 483.0655 for C_20_H_21_Cl_2_N_4_O_4_S). HPLC: *t*_r_ 7.60 min (95.6% at 254 nm).

##### 2-(3,4-Dichloro-5-methyl-1*H*-pyrrole-2-carboxamido)-4-(4-hydroxypiperidin-1-yl)benzo[*d*]thiazole-6-carboxylic Acid (**7p**)

Synthesized according to general procedure G with **6p** (145 mg, 0.28 mmol) as the reactant and using 2 M NaOH. The reaction mixture was stirred for 5 days. Additional 3 equiv of 2 M NaOH was added, and the reaction mixture was stirred at 40 °C for 2 days. Yield: 14 mg (10.8%); gray solid. ^1^H NMR (400 MHz, DMSO-*d*_6_): δ 1.57–1.73 (m, 2H), 1.88–2.00 (m, 2H), 2.29 (s, 3H), 2.95–3.08 (m, 2H), 3.63–3.74 (m, 1H), 3.75–3.87 (m, 2H), 7.49 (s, 1H), 8.19 (s, 1H), 11.90 (s, 1H), 12.42 (s, 1H), signal for OH and COOH groups are not seen. HRMS (ESI^–^) *m*/*z*: 467.0349 [M – H]^−^ (calcd *m*/*z*: 467.0353 for C_19_H_17_Cl_2_N_4_O_4_S). HPLC: *t*_r_ 5.78 min (95.7% at 254 nm).

##### 4-(4-(*tert*-Butoxycarbonyl)piperazin-1-yl)-2-(3,4-dichloro-5-methyl-1*H*-pyrrole-2-carboxamido)benzo[*d*]thiazole-6-carboxylic Acid (**7q**)

Synthesized according to general procedure G with **6q** (24 mg, 42 μmol) as the reactant. 10 equiv of 2 M NaOH (422 μmol, 211 μL) was used, and the reaction mixture was stirred only for 24 h. Yield: 12 mg, (51.3%); white powder. The crude product was used in the next step without further purification. HPLC: *t*_r_ 7.45 min (93.9% at 254 nm). MS (ESI) *m*/*z*: 552.0 ([M – H]^−^).

##### 2-(3,4-Dichloro-5-methyl-1*H*-pyrrole-2-carboxamido)-4-(1,4-dioxa-8-azaspiro[4.5]decan-8-yl)benzo[*d*]thiazole-6-carboxylic Acid (**7r**)

Synthesized according to general procedure G with **6r** (501 mg, 0.95 mmol) as the reactant and using 15 equiv of 1 M NaOH. Yield: 340 mg (96.2%); beige solid. ^1^H NMR (400 MHz, DMSO-*d*_6_): δ 1.82–1.91 (m, 4H), 2.28 (s, 3H), 3.45–3.54 (m, 4H), 3.95 (s, 4H), 7.51 (s, 1H), 8.22 (s, 1H), 11.90 (s, 1H), 12.39 (s, 1H), signal for COOH group is not seen. HRMS (ESI^+^) *m*/*z*: 511.0599 [M + H]^+^ (calcd *m*/*z*: 511.0604 for C_21_H_21_Cl_2_N_4_O_5_S). HPLC: *t*_r_ 6.81 min (94.0% at 254 nm).

##### 2-(3,4-Dichloro-5-methyl-1*H*-pyrrole-2-carboxamido)-4-(1-oxidothiomorpholino)benzo[*d*]thiazole-6-carboxylic Acid (**7s**)

Synthesized according to general procedure G with **6s** (270 mg, 0.54 mmol) as the reactant and using 1,4-dioxane as the solvent. The product was additionally purified by suspending it in methanol/THF (1:1) and filtering off the undissolved part. The solvent in the mother liquid was evaporated, and the residue was purified with flash column chromatography using DCM/methanol (7:1 to 4:1 to pureDCM) as the eluent. Yield: 15 mg (5.7%); beige solid. ^1^H NMR (400 MHz, DMSO-*d*_6_): δ 2.26 (s, 3H), 2.84–2.93 (m, 2H), 3.04–3.16 (m, 2H), 3.73–3.90 (m, 4H), 7.50 (s, 1H), 8.14 (s, 1H), 12.50 (s, 2H), signal for COOH group is not seen. HRMS (ESI^+^) *m*/*z*: 487.0047 [M + H]^+^ (calcd *m*/*z*: 487.0063 for C_18_H_17_Cl_2_N_4_O_4_S_2_). HPLC: *t*_r_ 5.49 min (97.2% at 254 nm).

##### 4-((*tert*-Butoxycarbonyl)(3-methoxypropyl)amino)-2-(3,4-dichloro-5-methyl-1*H*-pyrrole-2-carboxamido)benzo[*d*]thiazole-6-carboxylic Acid (**7t**)

Synthesized according to general procedure G with **6t** (291 mg, 0.509 mmol) as the reactant. 10 equiv of 1 M NaOH (5.1 mL, 10 equiv) was added, and the reaction was stirred at 40 °C overnight. Additional 5 equiv of 1 M NaOH (2.5 mL) was added, and the reaction mixture was stirred at 40 °C for another 24 h. Yield: 245 mg (86%), brown solid. ^1^H NMR (400 MHz, DMSO-*d*_6_): δ 1.23 (s, 6H), 1.47 (br s, 3H), 1.69 (p, *J* = 7.0 Hz, 2H), 2.28 (s, 3H), 3.33 (m, 2H), 3.49 (m, 5H), 7.77 (s, 1H), 8.55 (s, 1H), 12.09 (s, 1H), 12.39 (s, 1H), 13.00 (s, 1H).

#### General Procedure H: Synthesis of Compounds **8a–c** (with **8a** as an Example)

To a suspension of **7k** (39 mg, 0.070 mmol) in 1,4-dioxane (1 mL), 4 M HCl in 1,4-dioxane (2 mL) was added, and the reaction mixture was stirred at rt for 7 h. The precipitate in the reaction mixture was filtered off, and the crude product was purified with crystallization from methanol.

##### (*S*)-1-(6-Carboxy-2-(3,4-dichloro-5-methyl-1*H*-pyrrole-2-carboxamido)benzo[*d*]thiazol-4-yl)pyrrolidin-3-aminium Chloride (**8a**)

Yield: 23 mg (66.6%); brown solid. [α]_D_^25^ + 17.2 (*c* 0.064, DMF). ^1^H NMR (400 MHz, DMSO-*d*_6_): δ 2.05–2.16 (m, 1H), 2.29 (s, 3H), 3.66–3.80 (m, 3H), 3.86–4.05 (m, 3H), 7.11 (d, *J* = 1.6 Hz, 1H), 7.96 (d, *J* = 1.4 Hz, 1H), 8.20 (s, 3H), 11.73 (s, 1H), 12.65 (s, 1H), signal for COOH is not seen. HRMS (ESI^+^) *m*/*z*: 454.0498 [M + H]^+^ (calcd *m*/*z*: 454.0502 for C_18_H_18_Cl_2_N_5_O_3_S). HPLC: *t*_r_ 1.36 min (98.9% at 254 nm).

##### 4-(6-Carboxy-2-(3,4-dichloro-5-methyl-1*H*-pyrrole-2-carboxamido)benzo[*d*]thiazol-4-yl)piperazin-1-ium Chloride (**8b**)

Synthesized according to general procedure H with **7n** (12 mg, 0.216 mmol) as the reactant. The reaction was stirred for 48 h instead of 7 h and was periodically sonicated, and the progress was monitored by HPLC–MS. The crude product was washed with hexane and DCM and dried. Yield: 10 mg (96.5%); white powder. ^1^H NMR (400 MHz, DMSO-*d*_6_): δ 2.29 (s, 3H), 3.29–3.40 (m, 4H), 3.58–3.64 (m, 4H), 7.45 (d, *J* = 1.5 Hz, 1H), 8.27 (d, *J* = 1.5 Hz, 1H), 8.96 (s, 2H), 11.94 (s, 1H), 12.61 (s, 1H), 12.92 (br s, 1H). HRMS (ESI^+^) *m*/*z*: 454.0494 [M + H]^+^ (calcd *m*/*z*: 454.0502 for C_18_H_18_Cl_2_N_5_O_3_S). HPLC: *t*_r_ 4.59 min (95.2% at 254 nm).

##### 2-(3,4-Dichloro-5-methyl-1*H*-pyrrole-2-carboxamido)-4-((3-methoxypropyl)amino)benzo[*d*]thiazole-6-carboxylic Acid (**8c**)

Synthesized according to general procedure H with **7q** (235 mg, 0.42 mmol) as the reactant. The crude product was purified with 1,4-dioxane instead of methanol. Yield: 189 mg (98.0%); gray solid. ^1^H NMR (400 MHz, DMSO-*d*_6_): δ 1.90 (p, *J* = 6.5 Hz, 2H), 2.27 (s, 3H), 3.26 (s, 3H), 3.37 (t, *J* = 6.9 Hz, 2H), 3.45 (t, *J* = 6.0 Hz, 2H), 6.00 (br s, 1H), 7.33 (s, 1H), 8.00 (s, 1H), 11.78 (br s, 1H), 12.71 (s, 1H), signal for COOH group is not seen. ^13^C{^1^H} NMR (101 MHz, DMSO-*d*_6_): δ 11.47, 28.61, 58.49, 66.81, 70.31, 108.76, 110.35, 113.87, 115.73, 117.35, 127.51, 130.57, 131.90, 138.21, 140.57, 157.21, 159.47, 167.82. HRMS (ESI^+^) *m*/*z*: 457.0492 [M + H]^+^ (calcd *m*/*z*: 457.0499 for C_18_H_19_Cl_2_N_4_O_4_S). HPLC: *t*_r_ 6.85 min (98.7% at 254 nm).

##### Methyl 3-(Morpholinomethyl)-4-nitrobenzoate (**10**)

To the solution of methyl 3-formyl-4-nitrobenzoate (**9**, 1.05 g, 5.0 mmol) and morpholine (481 μL, 5.5 mmol) in 1,2-dichloroethene (25 mL), NaCNBH_3_ (413 mg, 6.25 mmol) and acetic acid (286 μL, 5.0 mmol) were added, and the reaction mixture was stirred at rt overnight. Water was added, and the pH was adjusted to 1 using 1 M HCl. The phases were separated, pH of the water phase was adjusted to 10 using 1 M NaOH, and the alkaline water phase was extracted with DCM. The organic phase was washed with brine, dried over Na_2_SO_4_, filtered, and the solvent was removed in vacuo. Yield: 604 mg (42.9%); yellow oil. ^1^H NMR (400 MHz, CDCl_3_): δ 2.43 (t, *J* = 4.5 Hz, 4H), 3.65 (t, *J* = 4.5 Hz, 4H), 3.79 (s, 2H), 3.97 (s, 3H), 7.81 (d, *J* = 8.3 Hz, 1H), 8.06 (d, *J* = 8.3 Hz, 1H), 8.20 (s, 1H). MS (ESI) *m*/*z*: 281.4 ([M + H]^+^).

##### Methyl 4-Amino-3-(morpholinomethyl)benzoate (**11**)

Synthesized according to general procedure C with **10** (604 mg, 2.15 mmol) as the reactant. Yield: 486 mg (90.1%); colourless oil. ^1^H NMR (400 MHz, CDCl_3_): δ 2.42 (m, 4H), 3.55 (s, 2H), 3.63–3.73 (m, 4H), 3.85 (s, 3H), 5.26 (br s, 2H), 6.60 (d, *J* = 8.2 Hz, 1H), 7.71 (d, *J* = 2.0 Hz, 1H), 7.79 (dd, *J* = 2.0, 8.4 Hz, 1H). MS (ESI) *m*/*z*: 251.1 ([M + H]^+^).

##### Methyl 2-Amino-4-(morpholinomethyl)benzo[*d*]thiazole-6-carboxylate (**12**)

Synthesized according to general procedure D with **11** (453 mg, 1.81 mmol) as the reactant. Instead of NH_3_ solution, the reaction mixture was neutralized with 4 M NaOH. The product was filtered off, washed with water, and dried. Yield: 351 mg (63.1%); orange solid. ^1^H NMR (400 MHz, DMSO-*d*_6_): δ 3.59 (m, 4H), 3.78 (m, 4H), 3.86 (s, 3H), 4.59 (s, 2H), 8.06 (s, 1H), 8.15 (s, 2H), 8.41 (s, 1H). MS (ESI) *m*/*z*: 308.1 ([M + H]^+^).

##### Methyl 2-(3,4-Dichloro-5-methyl-1*H*-pyrrole-2-carboxamido)-4-(morpholinomethyl)benzo[*d*]thiazole-6-carboxylate (**13**)

Synthesized according to general procedure F with **12** (154 mg, 0.501 mmol) as the reactant. After evaporation of the solvent, ethyl acetate and 10% citric acid were added to the reaction mixture, and the product was filtered off. 326 mg of brown powder was obtained which contained product and citric acid. The crude product was used in the next step without further purification. ^1^H NMR (400 MHz, DMSO-*d*_6_): δ 2.29 (s, 3H), 3.92 (s, 3H), 4.73 (s, 2H), 8.28 (s, 1H), 8.80 (s, 1H), 12.48 (s, 1H), signals for the morpholino group are overlapping with the signal for water and DMSO. MS (ESI) *m*/*z*: 483.1 ([M + H]^+^).

##### 4-((6-Carboxy-2-(3,4-dichloro-5-methyl-1*H*-pyrrole-2-carboxamido)benzo[*d*]thiazol-4-yl)methyl)morpholin-4-ium Chloride (**14**)

Synthesized according to general procedure G with crude **13** (326 mg, 0.67 mmol) as the reactant. 2 M NaOH was used instead of 1 M NaOH. In the isolation step, pH was first lowered to 7 with 1 M HCl, the mixture was filtered, and filtrate was acidified with 1 M HCl to pH = 2. The precipitate that formed was filtered off, washed with water and acetonitrile/methanol (2:1) mixture, and dried. Yield: 15 mg (4.4%); gray solid. ^1^H NMR (400 MHz, DMSO-*d*_6_): δ 3.68 (m, 4H), 3.95 (m, 4H), 4.75 (s, 2H), 8.27 (s, 1H), 8.77 (s, 1H), 10.17 (s, 1H), 11.99 (s, 1H), 12.62 (s, 1H), 13.15 (br s, OH). HRMS (ESI^+^) *m*/*z*: 469.0480 [M + H]^+^ (calcd *m*/*z*: 469.0499 for C_19_H_19_Cl_2_N_4_O_4_S). HPLC: *t*_r_ 4.79 min (95.2% at 254 nm).

##### Methyl 2-(4-Chloro-5-methyl-1*H*-pyrrole-2-carboxamido)-4-morpholinobenzo[*d*]thiazole-6-carboxylate (**15a**)

Synthesized according to general procedure F with 2,2,2-trichloro-1-(4-chloro-5-methyl-1*H*-pyrrol-2-yl)ethan-1-one (178 mg, 0.682 mmol) and **5a** (200 mg, 0.682 mmol) as reactants. The reaction mixture was stirred at 70 °C for 48 h. During isolation, after the addition of ethyl acetate and water, the precipitate formed which was filtered off, washed with ethyl acetate, and dried to give **9a**. Yield: 258 mg (87.0%); off-white solid. ^1^H NMR (400 MHz, DMSO-*d*_6_): δ 2.22 (s, 3H), 3.36–3.44 (m, 4H), 3.81–3.86 (m, 4H), 3.87 (s, 3H), 7.39–7.45 (m, 2H), 8.23 (d, *J* = 1.5 Hz, 1H), 12.19 (s, 2H). MS (ESI) *m*/*z*: 433.0 ([M – H]^−^).

##### Methyl 2-(4-Fluoro-5-methyl-1*H*-pyrrole-2-carboxamido)-4-morpholinobenzo[*d*]thiazole-6-carboxylate (**15b**)

Synthesized according to general procedure E with 4-fluoro-5-methyl-1*H*-pyrrole-2-carboxylic acid (61 mg, 0.426 mmol) and **5a** (125 mg, 0.426 mmol) as reactants. The crude product was suspended in methanol, heated, and filtered off. Yield: 70 mg (39.3%); white solid. ^1^H NMR (400 MHz, DMSO-*d*_6_): δ 2.20 (s, 3H), 3.38–3.40 (m, 4H), 3.81–3.86 (m, 4H), 3.87 (s, 3H), 7.26 (d, *J* = 3.0 Hz, 1H), 7.41 (d, *J* = 1.6 Hz, 1H), 8.23 (d, *J* = 1.5 Hz, 1H), 11.85 (s, 1H), 12.42 (s, 1H). MS (ESI) *m*/*z*: 419.0 ([M + H]^+^).

##### Methyl 2-(4-Cyano-5-methyl-1*H*-pyrrole-2-carboxamido)-4-morpholinobenzo[*d*]thiazole-6-carboxylate (**15c**)

Synthesized according to general procedure E with 4-cyano-5-methyl-1*H*-pyrrole-2-carboxylic acid (70 mg, 0.466 mmol) and **5a** (139 mg, 0.466 mmol) as reactants. The crude product was extensively washed with hot methanol. Yield 90 mg (45%); brown solid. ^1^H NMR (400 MHz, DMSO-*d*_6_): δ 2.40 (s, 3H), 3.39 (s, 4H), 3.79–3.89 (m, 7H), 7.42 (s, 1H), 7.71 (s, 1H), 8.25 (s, 1H), 12.72 (s, 1H), 12.74 (s, 1H). MS (ESI) *m*/*z*: 425.9 ([M + H]^+^).

##### 2-(4-Chloro-5-methyl-1*H*-pyrrole-2-carboxamido)-4-morpholinobenzo[*d*]thiazole-6-carboxylic Acid (**16a**)

Synthesized according to general procedure G with **15a** (200 mg, 0.460 mmol) as the reactant and stirring the reaction mixture for 48 h. Yield: 150 mg (77.5%); pale-yellow solid. ^1^H NMR (400 MHz, DMSO-*d*_6_): δ 2.22 (s, 3H), 3.38 (s, 4H), 3.84 (t, *J* = 4.5 Hz, 4H), 7.40–7.45 (m, 2H), 8.19 (d, *J* = 1.5 Hz, 1H), 12.18 (s, 1H), 12.44 (s, 1H), 12.86 (s, 1H). ^13^C{^1^H} NMR (101 MHz, DMSO): δ 10.42, 50.38, 66.36, 109.84, 113.45, 116.56, 120.83, 126.34, 131.75, 133.10, 143.52, 143.74, 158.15, 159.01, 163.14, 167.42. HRMS (ESI^–^) *m*/*z*: 419.0586 [M – H]^−^ (calcd *m*/*z*: 419.0586 for C_18_H_16_ClN_4_O_4_S). HPLC: *t*_r_ 5.82 min (96.7% at 254 nm).

##### 2-(4-Fluoro-5-methyl-1*H*-pyrrole-2-carboxamido)-4-morpholinobenzo[*d*]thiazole-6-carboxylic Acid (**16b**)

Synthesized according to general procedure G with **15b** (50 mg, 0.119 mmol) as the reactant. After 24 h, additional 5 equiv of 1 M NaOH was added, and the reaction mixture was stirred at 50 °C for 72 h. Yield: 29 mg (60.4%); white solid. ^1^H NMR (400 MHz, DMSO-*d*_6_): δ 2.20 (s, 3H), 3.34–3.44 (m, 4H), 3.84–3.86 (m, 4H), 7.25 (d, *J* = 2.9 Hz, 1H), 7.45 (d, *J* = 1.6 Hz, 1H), 8.20 (d, *J* = 1.5 Hz, 1H), 11.85 (s, 1H), 12.40 (s, 1H), signal for COOH group is not seen. ^13^C{^1^H} NMR (101 MHz, DMSO-*d*_6_): δ 8.69, 50.21, 66.13, 100.68, 100.84, 113.21, 116.49, 116.57, 116.65, 118.78, 119.02, 126.10, 132.93, 143.17, 143.58, 146.66, 149.01, 158.39, 158.97, 167.19. HRMS (ESI^–^) *m*/*z*: 403.0880 [M – H]^−^ (calcd *m*/*z*: 403.0882 for C_18_H_16_FN_4_O_4_S). HPLC: *t*_r_ 6.33 min (97.1% at 254 nm).

##### 2-(4-Cyano-5-methyl-1*H*-pyrrole-2-carboxamido)-4-morpholinobenzo[*d*]thiazole-6-carboxylic Acid (**16c**)

Synthesized according to general procedure G with **15c** (87 mg, 0.204 mmol) as the reactant. 2 M LiOH was used instead of 1 M NaOH. After 24 h, additional 5 equiv of 2 M LiOH was added, and the reaction mixture was stirred overnight. Yield 61 mg (73%); pale-yellow solid. ^1^H NMR (400 MHz, DMSO-*d*_6_): δ 2.39 (s, 4H), 3.38 (s, 4H), 3.84 (s, 5H), 7.43 (s, 1H), 7.70 (s, 1H), 8.21 (s, 1H), 12.69 (s, 1H), 12.74 (s, 1H), 12.88 (s, 1H). ^13^C{^1^H} NMR (101 MHz, DMSO-*d*_6_): δ 11.16, 49.65, 65.72, 91.30, 112.49, 115.45, 115.74, 116.03, 123.04, 125.87, 132.39, 142.27, 142.94, 143.23, 157.43, 157.97, 166.71. HRMS (ESI^+^) *m*/*z*: 412.1070 [M + H]^+^ (calcd *m*/*z*: 412.1074 for C_19_H_18_N_5_O_4_S). HPLC: *t*_r_ 4.97 min (98.2% at 254 nm).

### Enzymology

#### Determination of Inhibitory Activities on *E. coli* and *P. aeruginosa* DNA Gyrase and *E. coli* Topoisomerase IV

The assay to determine IC_50_ values was performed using assay kits from Inspiralis in accordance with previously described procedures.^43^ Seven concentrations of the inhibitors were used to determine IC_50_ values which were then calculated with GraphPad Prism 6.0 software. Three independent measurements were performed to determine IC_50_ values, and mean values are reported as the final result.

#### Determination of Inhibitory Activities on *S. aureus* and *A. baumannii* DNA Gyrase and Topoisomerase IV and *P. aeruginosa* Topoisomerase IV

Inhibitory activities of **7a** against *S. aureus* and *A. baumannii* DNA gyrase and topo IV and *P. aeruginosa* topo IV were determined at Inspiralis Ltd. (Norwich, UK) using gel-based supercoiling (for gyrase) and decatenation (for topo IV) assays. Details on these assays can be found in the Supporting Information.

#### Determination of Inhibitory Activity on Human DNA Topoisomerase IIα

The assay to determine IC_50_ value for **7a** was performed using assay kits from Inspiralis according to previously described procedures.^44^ Seven concentrations of the inhibitors were used to determine IC_50_ values which were then calculated with GraphPad Prism 6.0 software. Three independent measurements were performed to determine IC_50_ values, and mean values are reported as the final result.

#### Selectivity Profiling against Protein Kinases

The kinase inhibition profile of **7a** was determined at Reaction Biology Europe GmbH (Freiburg, Germany). A radiometric protein kinase assay (^33^PanQinase activity assay) was used for measuring the kinase activity of the 335 protein kinases. For details on protein kinase assay, see the Supporting Information.

### Determination of Antibacterial Activity

MICs (Tables 1 and S1–S3) were determined with a standard serial broth microdilution technique according to Clinical and Laboratory Standards Institute (CLSI) guidelines.^45^ Cation-adjusted Mueller Hinton broth 2 (MHBII) was used for bacterial growth under standard laboratory conditions and for antimicrobial susceptibility tests. MHBII broth was prepared by dissolving 22 g of MHBII powder [containing beef extract (3 g), acid hydrolysate of casein (17.5 g), and starch (1.5 g); Becton Dickinson and Co.] in 1 L of water. To prepare MHBII agar, 14 g of agar (Bacto; Molar Chemicals) was added to 1 L of broth.

Bacterial strains were inoculated onto MHBII agar plates and grown overnight at 37 °C. Next, three individual colonies from each strain were inoculated into 1 mL of MHBII medium and propagated at 37 °C overnight, with agitation at 250 rpm. For *Enterococcus* sp., the cells were plated in brain–heart infusion [(BHI) medium] agar plates, and BHI broth was used to determine the MICs. To perform the MIC assays, 12-step serial dilutions using two-fold dilution steps of the given compound (each dissolved in 100% DMSO) were generated in 96-well microtiter plates (Corning Inc.). The highest final DMSO concentration in the experiments was 0.64% (in the case of the 64 μg/mL concentration of the inhibitor). Following the dilutions, each well was seeded with 5 × 10^4^ bacterial cells (in 100 μL final volume). Each measurement was performed in three parallel replicates, and to avoid possible edge effects in the microwell plates, the outside rows (A, H) were filled with sterile medium. Following the inoculations, the plates were covered with the lids and wrapped in polyethylene plastic bags, to minimize evaporation but to allow O_2_ transfer. The plates were incubated at 37 °C under continuous shaking at 150 rpm for 18 h. After incubation, the OD_600_ of each well was measured using a microplate reader (Synergy 2; Biotek). The MICs were defined as the antibiotic concentrations which inhibited the growth of the bacterial cultures; that is, the drug concentration where the average OD_600_ increment of the three technical replicates was below 1.5-fold the background OD increment.

The expanded panel antibacterial spectrum of **7a** and **7h** and comparator antibiotics (Figures 4 and 5 and Tables S4–S11) were tested at IHMA Europe Sàrl (Switzerland) in broth microdilution assays according to the corresponding CLSI guidelines.^45^ The panel of Hungarian clinical isolates and controls measured following the same method as detailed above, and albeit measurements were carried out in 384-well microtiter plates (Corning Inc.).

### Frequency-of-Resistance Assay

To determine the spontaneous frequency-of-resistance, approximately 10^10^ cells from stationary-phase MHBII broth cultures of *S. aureus* ATCC 700699 and *S. aureus* ATCC 43300 were plated to antibiotic-containing plates according to a standard protocol to determine frequency-of-resistance.^28,46−48^ Prior to plating, bacteria were grown overnight in MHBII medium at 37 °C, 250 rpm, collected by centrifugation, and washed once in equal volumes of MHBII broth. From this concentrated cell suspension (250 μL), approximately 10^10^ cells were then plated to each MHBII agarose plates. Using agarose instead of agar, reduced drug adsorption and improved the performance of the assay. Petri-dishes (145 mm) were filled with 40 mL of MHBII agarose medium containing the selective drug at the desired concentration (i.e., 2×, 4×, 8×, and 20× MIC of each given antibiotic). All experiments were performed in at least three replicates. Plates were grown at 37 °C for 72 h. Total cfus were determined simultaneously in each experiment by plating appropriate dilutions to antibiotic-free MHBII agar plates. Finally, resistance frequencies for each strain were calculated by dividing the number of colonies formed after a 72 h incubation at 37 °C by the initial viable cell count. From each strain-antibiotic pair, 10–10 colonies were collected from each replicate from the highest selection concentration. After that, we pooled together the 10 colonies and measured the MIC of each population representing one parallel of each strain-antibiotic pair.

### Plasma Protein Binding

The PPB studies were performed by Selvita d.o.o. (Zagreb, Croatia) in mouse plasma (CD-1 male, pooled, commercially obtained from BioIVT) via equilibrium dialysis. Compounds were assayed at 5 μM (0.5% DMSO) in two replicates, and incubation time was 4 h at 37 °C. Samples were taken at 0 min and 4 h. Aliquots from both buffer and plasma compartment matrix were matched and mixed with 6 volumes of STOP solution (acetonitrile/methanol (2:1) + internal standard – diclofenac) and analyzed by LC–MS/MS. Assay controls were caffeine (low), verapamil (moderate binding), and nicardipine (very high binding), and protein contamination was checked. No protein contamination was observed in the samples from test compounds.

### Thermodynamic Solubility Assay

Thermodynamic solubility was determined as a concentration of a saturated solution in equilibrium (37 °C) in phosphate-buffered saline (PBS) (pH = 7.4) using a HPLC method.

For the PBS, 2.38 g of disodium hydrogen phosphate dodecahydrate, 0.19 g of potassium dihydrogen phosphate, and 8.0 g of sodium chloride were added to distilled water (900 mL), and the solution was mixed overnight. The next day, it was diluted to 1000 mL with the same solvent, and the pH was adjusted to 7.4. Samples were prepared by weighing the exact mass of solid compounds (about 1 mg), and then, the appropriate volume of PBS was added to reach the final concentration of ∼1 mg/mL. The samples were incubated at 600 rpm, in a 37 °C orbital shaking incubator for 24 h. After 24 h, they were centrifuged at 18,000 rpm for 10 min. The supernatant was diluted 3× with a 1:1 mixture of 0.1% TFA in water and acetonitrile and analyzed by HPLC. For the 7-point calibration curves, concentrated stock solutions of the compounds were prepared at concentrations of 10 and 0.5 mM in DMSO, which were diluted with a 1:1 mixture of 0.1% TFA in water and acetonitrile to the desired final concentrations and analyzed by HPLC. Results are given in μM as an average value of two independent experiments. For details on calibration curves, concentrations, analytical method, and HPLC method, see the Supporting Information.

### Log*D* Determination

Log*D* of **7a** was determined in HPLC vials with the miniaturized shake-flask method. The used solutions were 0.05 M potassium phosphate at pH 7.4 (KP) saturated with octanol and octanol saturated with KP. The phase ratios used were 1:1 (0.8 mL octanol + 0.8 mL KP) and 1:3 (0.4 mL octanol + 1.2 mL KP). First, **7a** was dissolved in DMSO to a 10 mM stock solution, and 1.6 μL of the solution was pipetted into an HPLC vial. Then, octanol was added followed by KP. The vial was sealed, vortexed, and phase separation was set during 48 h at room temperature (∼23 °C) in the dark. After separation, the octanol phase was transferred to another vial. Both phases were analyzed with a standard curve-based method by liquid chromatography coupled to triple quadrupole mass spectrometry (LC–MS/MS).

#### Liquid Chromatography Coupled to Triple Quadrupole Mass Spectrometry (LC–MS/MS)

The test compound was optimized on a Waters Acquity UPLC XEVO TQ-S microsystem (Waters Corporation) running in the multiple reaction monitoring mode with negative or positive electrospray ionization using QuanOptimize software (Waters Corporation).

The column used for chromatographic separation was a C18 BEH 1.7 μm column, with 0.5 mL/min flow rate; 5 μL sample injection volume; mobile phase A: 5% acetonitrile and 0.1% formic acid in purified water; mobile phase B: 0.1% formic acid in acetonitrile; general gradient: 1% to 90% (for B); 2 min total running time.

### X-ray Crystallography

X-ray structure of **7a** in complex with *P. aeruginosa* GyrB24 was obtained at 1.6 Å resolution according to our published procedures.^34^ Additional details on protein expression and purification, crystallization, X-ray data collection, and structure solution can be found in the Supporting Information.

### Metabolic Stability Assay

Metabolic stability was determined in mouse hepatocytes and human and mouse liver microsomes.

#### Mouse Hepatocytes

The assay was performed in cryopreserved mouse hepatocytes. First, the vials were thawed, the suspension added to “thawing media” and centrifuged to pellet the hepatocytes. The cell pellet was resuspended in Williams medium E (Invitrogen A1217601). Sample of **7a** was prepared from 10 mM DMSO stock solution, which was first diluted to 1 mM in DMSO and tested at a final concentration of 1 μM (0.1% final DMSO concentration). A mixture of midazolam, diclofenac, and bufuralol at 1 μM was used as a positive control. The incubation was carried out on a heater-shaker at 37 °C, in a 24-well Picoplate (round bottom, PerkinElmer), with total incubation volume of 700 μL. First, 0.7 μL of 1 mM DMSO stock of **7a** was pipetted to the wells, and then, cell suspension at a density of 1.0 × 10^6^ cells/mL (in 700 μL) was added to initiate the reaction. The plate was incubated at 37 °C for 60 s. A zero sample was taken by removing 100 μL to a 96-well plate, and the reaction was quenched by adding 100 μL of acetonitrile with 50 nM warfarin as the internal standard. Consecutive samples were taken at 5, 15, 30, 60, and 90 min. Prior to analysis by LC–MS/MS, the samples were centrifuged at 3500 rpm for 15 min. The LC–MS/MS method was the same as described above in Chapter LogD Determination.

#### Human and Mouse Liver Microsomes

Liver microsomal fractions were purchased from XenoTech LLC, (KS, USA). Metabolic stability was determined in 100 mM KPO_4_ buffer (pH 7.4) in 0.5 mg/mL human and mouse liver microsomes with total incubation volume of 500 μL. The final tested concentration of **7a** was 1 μM. To initiate the reaction, 1 mM NADPH was added. Samples were taken at 0, 5, 10, 20, 40, and 60 min. To terminate the reaction, cold acetonitrile with warfarin as an internal standard was added. LC–MS/MS was used to analyze the samples and determine the amount of remaining parent compound. The LC–MS/MS method was the same as described above in Chapter LogD Determination.

#### Calculations

The in vitro data were calculated essentially according to Obach et al.,^49^ using Barter et al.^50^ for scaling factors. fu measured in mouse is assumed to be the same for human, and the blood to plasma ratio = 1. The predicted fraction unbound in the incubation (cell and microsome) was calculated using fu_mic/hep_ = 1/(1 + *C*_prot/cell_ × 10^0.56×log*P*/*D*–1.41^).^51^

### In Vivo Pharmacokinetic Studies

The in vivo pharmacokinetic studies of **7a** were performed by Selvita d.o.o. (Zagreb, Croatia) in male CD-1 mice (Charles River). The test compound was administered via intravenous (iv) bolus at a dose of 1 mg/kg (dose volume 5 mL/kg). Blood samples were collected from the tail vein 0.05, 0.25, 0.5, 1.0, 2.0, 4.0, 8.0, and 24 h after administration from three animals per time point. Each animal was sampled 2–4 times throughout the time course of collection (six animals were used totally per each route). Blood samples were centrifuged at 1560*g* for 10 min at 4 °C. The samples were analyzed by LC–MS/MS. Prior to analysis, the samples were prepared by protein precipitation {20 μL of plasma + 20 μL of water + 240 μL of extraction solvent [acetonitrile/methanol (2:1 v/v) with 100 ng/mL diclofenac (IS)]}, and the extracts were diluted with water (1:3 v/v).

For LC–MS/MS analysis, a Shimadzu prominence HPLC system was used (HPLC pump: LC30AD, autosampler: SIL30ACMP, column oven: CTO30A) connected to an Applied Biosystems mass spectrometer (QTRAP 5500) operating in the positive TurboIonSpray mode. For separation, a Waters Acquity UPLC BEH C18 1.7 μm, 2.1 × 50 mm (Waters, Milford, MA) column was used, with an injection volume of 1 or 2 μL and a flow rate of 0.5 mL/min. The mobile phase consisted of 0.1% formic acid in water (solvent A) and 0.1% formic acid in acetonitrile (solvent B). Gradient: 0–0.05 min, 5% B; 0.05–0.80 min, 5–85% B; 0.80–1.00, 85% B; 1.00–1.01, 85–95% B; 1.01–1.50, 95% B; 1.50–1.51, 95–5% B, 1.51–2.00, 5% B.

Pharmacokinetic analysis was performed using WinNonlin Phoenix software (Certara, version 8.3) from the mean animal plasma concentration per group, per time point, non-compartmental analysis, and the target dose.

All animals were managed similarly and with due regard for their well-being according to prevailing practices and the experimental plan approved by Committee for Animal Research Ethics (CARE) Zagreb.

### In Vitro Cytotoxicity Measurements

To determine the cytotoxicity of inhibitor **7a** on HepG2 and MCF-7 cell lines, the MTS (Promega, Madison, WI, USA) assay was used according to manufacturer instructions. Briefly, Eagle’s minimum essential medium (Gibco, Thermo Fisher Scientific, Waltham, MA, USA) was used to culture the cells. The following supplements were added to the medium: l-glutamine (2 mM; Sigma-Aldrich, St. Louis, MO, USA), penicillin (100 U/mL; Sigma-Aldrich, St. Louis, MO, USA), streptomycin (100 μg/mL; Sigma-Aldrich, St. Louis, MO, USA), and fetal bovine serum (10%; Gibco, Thermo Fisher Scientific, Waltham, MA, USA). The cells were maintained at 37 °C in a humidified 5% CO_2_ atmosphere. The cells were plated in 96-well plates at a density of 2000 cells per well and incubated for 24 h. Then, the cells were treated with selected compounds, positive control (50 μM etoposide), or vehicle control (0.5% DMSO) and incubated for 72 h. The CellTiter96 Aqueous One Solution Reagent (10 μL; Promega, Madison, WI, USA) was then added to each well, and the plate was incubated for another 3 h. Absorbance at 490 nm was measured with a BioTek Synergy 4 Hybrid Microplate Reader (Winooski, VT, USA). Independent experiments were repeated three times, each performed in triplicate. Statistical significance (*p* < 0.05) was calculated with two-tailed Student’s *t* test between treated groups and DMSO. IC_50_ values (concentration of the compound that gives a half-maximal response) are given as average values from the independent measurements and were determined using GraphPad Prism 5.0 software (San Diego, CA, USA)

### In Vitro Cell Micronucleus Test

Genetic toxicity analysis of compound **7a** was performed at Medina (Fundación Medina, Granada, Spain) in an in vitro micronucleus test (MNT) using a protocol that follows the recommendations of OECD Test Guideline 487 (TG-487) for the testing of chemicals.^52^ Details of the MNT assay can be found in the Supporting Information.

### Mitochondrial Toxicity Assay

In vitro mitochondrial toxicity of **7a** was tested in RISE (Research Institutes of Sweden) with glu–gal assay. **7a** was assayed using HepG2 cells cultured in either glucose or galactose according to the published protocols.^53,54^ HepG2 cells were seeded in two 96-well plates at a density of 50,000 cells per well in 100 μL of growth medium. For the first plate, the growth medium contained glucose and for the second plate, it contained galactose. Cells were incubated overnight in a 5% CO_2_ atmosphere at 37 °C and allowed to attach onto the wells. After 24 h, medium was removed, wells were washed with PBS, and 100 μL of medium (containing glucose for plate 1 and galactose for plate 2) was added prior to the addition of test compound. The medium contained positive control (Rotenone). Plates were then incubated for another 24 h upon which 100 μL of CellTiter-Glo 2.0 cell viability assay (Promega, Madison, WI, USA) was added to the wells to measure the amount of ATP. The plates were shaken for 2 min and incubated at room temperature for 15 min in the dark. Luminescence was measured with Tecan Infinite 200 pro luminometer, and the values from each well treated with compound were compared to the values given by the negative control (medium only). The inhibition of mitochondrial function was determined by comparing ATP levels in galactose and glucose exposed cells. The compound was assayed at 10, 50, 100, 300, 600, and 1000 μM concentrations in six replicates.

### Na_V_1.5 and hERG Ion Channel Cardiac Safety Assays

hERG and Na_V_1.5 inhibition was measured with the patch-clamp method on stably transfected CHO cells according to previously described procedures.^34^ The test compound **7a** was assayed at 50 μM (hERG) or 10 μM (Na_V_1.5). The normalized hERG/Na_V_1.5 current is reported as mean ± standard error of the mean (SEM, *N* = 5–6).

hERG inhibition was additionally determined in whole-cell patch-clamp experiments on HEK293 cells stably expressing the channel, as previously described.^55^ The peak current upon repolarization to −40 mV was monitored under the continuous perfusion of 50 μM of **7a**. No inhibitory effect was observed (current reduction: 0 ± 12%, mean ± SD, *N* = 6) under these conditions.

### Hemolysis Assay

**7a** was evaluated for hemolytic activity using red blood cells (RBCs) from heparinized human blood. RBCs were washed three times in Tyrode buffer (130 mM NaCl, 4 mM KCl, 2.8 mM Na acetate, 1 mM MgCl_2_, 10 mM HEPES, 10 mM glucose, 1 mM CaCl_2_, adjusted to pH 7.4) and resuspended in the same buffer. Final concentrations in the hemolysis assay were 100 μM compound, 1% DMSO, and 50% RBC, assayed in a 200 μL volume in a microtiter plate. The mixture was incubated at 37 °C for 45 min with shaking (250 rpm). After incubation, RBCs were removed by centrifugation, clear plasma was transferred to a fresh plate, and the amount of hemoglobin was measured using a spectrophotometer at 540 nm. The complete lysis control contained 2% Triton X-100 (in Tyrode buffer) instead of compound; the negative control contained Tyrode buffer but no compound. Percent hemolysis was calculated as: [Abs compound] – [Abs negative control]/[Abs complete lysis control] – [Abs negative control] × 100. Values greater than 1% hemolysis at 100 μM were regarded as a red flag.

### Formulation Assay

Formulation work was carried out in RISE (Research Institutes of Sweden). Melting point of **7a** was determined with the differential scanning calorimetry technique on a Mettler Toledo DCS 822^e^ calorimeter using 40 μL aluminum crucibles with lid (open). The heating rate was 10 K/min, and temperature intervals were 25–350 and 25–450 °C.

X-ray powder diffraction analyses were performed at room temperature on a PANalytical X’Pert PRO instrument, equipped with a Cu X-ray tube and a PIXcel detector. Automatic divergence- and anti-scatter slits were used together with 0.02 rad Soller slits and a Ni-filter. To increase the randomness, the samples were spun during the analyses. The samples were analyzed on cut silicon zero background holders (ZBH), scan range 2–40 [° 2θ]. The scan rate was 17 min.

Solubility analysis was performed on an Acquity UPLC instrument (Waters, Milford, MA, USA). A Waters BEH C18 column (2.1 × 50 mm, 1.7 μm; Waters) was used at *T* = 40 °C, with the flow rate of 0.5 mL/min; PDA UV detection at λ = 256 nm; mobile phase A: 0.1% formic acid in ultrapure water; mobile phase B: 0.1% formic acid in acetonitrile. Gradient: 0–4 min, 5–100% B; 4–5 min, 100% B; 5–5.1 min, 100–5% B; 5.1–7 min, 5% B.

Osmolality was measured with a FiskeMicro-Osmometer, model 210 (Advanced Instruments, MA, USA).

### In Vivo Assay

The neutropenic mouse thigh infection model study was performed by NeoSome Life Sciences, LLC (Lexington, MA, USA) according to NeoSome Institutional Animal Care and Use Committee (IACUC) policies and guidelines as well as NIH’s Office of Laboratory Animal Welfare (OLAW) standards.

Neutropenic mouse thigh infection experiments were performed on female CD-1 mice (Charles River Laboratories, USA). To induce neutropenia, mice received two doses of cyclophosphamide on days −4 and −1 with 150 and 100 mg/kg delivered intraperitoneally (IP), respectively. The inoculum of the testing strain, *S. aureus* ATCC 700699-P1 (VISA), was prepared from overnight agar plate cultures. To prepare bacterial inoculums, a portion of the plate was resuspended in sterile saline and adjusted to an OD of 0.12 at 625 nm. Next, the resulted bacterial suspension was further diluted to reach an infecting inoculum of 6.0 × 10^5^ cfu per each mouse. Plate counts of the inoculum were also performed in each case to confirm the inoculum concentration, and the actual inoculum size was 7.25 × 10^5^ cfu/mouse. Mice were inoculated with 100 μL of the prepared bacterial suspension via an intramuscular injection into the right rear thigh. Prior to infection, **7a** was dissolved in a formulation of 20% hydroxypropyl β-cyclodextrin prepared with 100 mM sodium bicarbonate (pH 8.4). The test agent was then dosed via IV administration, to ensure optimal distribution, at 2, 10, and 18 h post-infection. Beginning at 2 h, post-infection mice were dosed with either test article or positive control antibiotic. Mice receiving test agents were dosed intravenously at 10 mL/kg. Linezolid was delivered as a SC dose to ensure optimized exposure, distribution, and efficacy. Four animals were dosed per group. One group of four mice was euthanized at the initiation of therapy (*t* = 2 h) and cfus determined. All remaining mice were euthanized at 26 h post-infection. At termination, thighs were aseptically excised, weighed, and homogenized to a uniform consistency in 2 mL of sterile saline. The homogenate was serially diluted and plated on bacterial growth media. The cfus were enumerated after overnight incubation.

## Supporting Information Available

Enzyme inhibition and antibacterial activity data for type II and type III compounds; more MIC data for **7a** and **7h**; enzyme inhibition graphs and gel images for **7a**; selectivity data (IC_50_ against hTopoIIα and residual activities of kinases) and cytotoxicity, mitotoxicity, genotoxicity, cardiotoxicity, and hemolytic activity data; in vivo efficacy data; data on preformulation studies and additional thermodynamic solubility data; details on methods for determining enzyme inhibition, thermodynamic solubility, details on protein kinase assay, X-ray crystallography, and micronucleus test; ^1^H and ^13^C NMR spectra of representative final compounds; and HRMS data and HPLC traces for lead compound **7a** (PDF)
Assay conditions for kinase assay (XLSX)
MIC data for 100 *A. baumannii* strains (XLSX)
MIC data for *N. gonorrhoeae*, *H. influenzae*, *E. faecium*, and *S. pneumoniae* (XLSX)
Molecular formula strings (CSV)

## Accession Codes

PDB ID codes: the coordinates and experimental data for the PaGyrB24-**7a** complex have been deposited in the Protein Data Bank with accession code 8BN6.

## Notes

Authors will release the atomic coordinates and experimental data upon article publication.

## Notes

Disclaimer: the views expressed in this article are the views of the authors, and neither IMI nor the European Union or EFPIA is responsible for any use that may be made of the information contained herein.

## Abbreviations Used

1,2-DCE: 1,2-dichloroethene
ATCC: American Type Culture Collection
BHI: brain–heart infusion medium
CDC: Centers for Disease Control and Prevention
CFU: colony-forming unit
CLSI: Clinical and Laboratory Standards Institute
DCM: dichloromethane
DIPEA: *N*,*N*-diisopropylethylamine
DMAP: 4-dimethylaminopyridine
DMF: *N*,*N*-dimethylformamide
DMSO: dimethylsulfoxide
FBS: fetal bovine serum
GHKL: gyrase, Hsp90, histidine kinase, MutL
GyrA: DNA gyrase subunit A
GyrB: DNA gyrase subunit B
HepG2: human hepatocellular carcinoma cell line
hTopoIIα: human DNA topoisomerase IIα
MCF-7: breast cancer cell line
MDR: multidrug-resistant
MHBII: Mueller Hinton II broth
MRSA: methicillin-resistant *Staphylococcus aureus*
MTS: 3-(4,5-dimethylthiazol-2-yl)-5-(3-carboxymethoxyphenyl)-2-(4-sulfophenyl)-2*H*-tetrazolium
ParC: topoisomerase IV subunit C
ParE: topoisomerase IV subunit E
PBS: phosphate-buffered saline
PDR: pandrug-resistant
RA: residual activity
TFA: trifluoroacetic acid
THF: tetrahydrofuran
Topo IV: topoisomerase IV
VISA: vancomycin-intermediate *S. aureus*
WHO: World Health Organization
