# Supplementary material for: New Dual Inhibitors of Bacterial Topoisomerases with Broad-Spectrum Antibacterial Activity and In Vivo Efficacy against Vancomycin-Intermediate Staphylococcus aureus
Source: J Med Chem. 2023 Mar 6;66(6):3968–94. doi: 10.1021/acs.jmedchem.2c01905 (PMC10041525; doi:10.1021/acs.jmedchem.2c01905)
Supplement: Supplementary file 1 — jm2c01905_si_001.pdf [file jm2c01905_si_001.pdf]

SUPPORTING INFORMATION

New dual inhibitors of bacterial topoisomerases with broad-spectrum antibacterial activity and *in vivo* efficacy against vancomycin-intermediate *Staphylococcus aureus*

Martina Durcik,[1] Andrej Emanuel Cotman,[1] Žan Toplak,[1] Štefan Možina,[1,◊] Žiga Skok,[1,◊] Petra Eva Szili,[2] Márton Czikkely,[2] Elvin Maharramov,[2] Thu Hien Vu,[2] Maria Vittoria Piras,[1] Nace Zidar,[1] Janez Ilaš,[1] Anamarija Zega,[1] Jurij Trontelj,[1] Luis A. Pardo,[3] Diarmaid Hughes,[4] Douglas Huseby,[4] Tália Berruga-Fernández,[4] Sha Cao,[4] Ivailo Simoff,[5] Richard Svensson,[5] Sergiy V. Korol,[6] Zhe Jin,[6] Francisca Vicente,[7] Maria C. Ramos,[7] Julia E. A. Mundy,[8] Anthony Maxwell,[8] Clare E. M. Stevenson,[8] David M. Lawson,[8] Björn Glinghammar,[9] Eva Sjöström,[10] Martin Bohlin,[10] Joanna Oreskär,[10] Sofie Alvér,[10] Guido V. Janssen,[11] Geert Jan Sterk,[11] Danijel Kikelj,[1] Csaba Pal,[2] Tihomir Tomašič,[*,1] Lucija Peterlin Mašič[*,1]

[1] University of Ljubljana, Faculty of Pharmacy, Aškerčeva cesta 7, 1000 Ljubljana, Slovenia

[2] Synthetic and Systems Biology Unit, Institute of Biochemistry, Biological Research Centre, Szeged H-6726, Hungary

[3] Max Planck Institute for Multidisciplinary Sciences, Oncophysiology, Hermann-Rein-Str. 3, 37075 Göttingen, Germany

[4] Uppsala University, Department of Medical Biochemistry and Microbiology, Husargatan 3, 75123 Uppsala, Sweden

[5] Uppsala University, Drug Optimization and Pharmaceutical Profiling Platform (UDOPP) Department of Pharmacy, Husargatan 3, 75123 Uppsala, Sweden

[6] Uppsala University, Department of Medical Cell Biology, Husargatan 3, 75123 Uppsala, Sweden

[7] Fundación Medina, Avenida del Conocimiento 34, Parque Tecnológico Ciencias de la Salud, 18016 Granada, Spain

[8] Department of Biochemistry and Metabolism, John Innes Centre, Norwich Research Park, Norwich NR4 7UH, U.K.

[9] RISE Research Institutes of Sweden, Department of Chemical and Pharmaceutical Toxicology, 15136 Södertälje, Sweden

[10] RISE Research Institutes of Sweden, Department of Chemical Processes and Pharmaceutical Development, 15136 Södertälje, Sweden

[11] Vrije Universiteit Amsterdam, Medicinal Chemistry Division, De Boelelaan 1108, 1081 HZ Amsterdam, The Netherlands

*Corresponding author (e-mail: Tihomir.Tomasic@ffa.uni-lj.si (T.T.), Lucija.PeterlinMasic@ffa.uni-lj.si (L.P.M.))

Table of contents

Table of contents S2

Enzyme inhibition and antibacterial activity S3

Thermodynamic solubility and plasma protein binding S8

In-depth microbiological profiling of **7a** and **7h** S9

Selectivity and toxicity data S19

In vivo and formulation studies S30

Determination of inhibitory activities on *S. aureus* and *A. baumannii* DNA gyrase and topoisomerase IV and *P. aeruginosa* topoisomerase IV S32

Protein kinase assay S34

Thermodynamic solubility assay S36

X-ray crystallography S39

In vitro cell micronucleus test S42

^{1}H and ^{13}C NMR spectra for representative compounds S44

HRMS data for lead compound **7a** S56

HPLC traces for lead compound **7a** S58

References S59

Enzyme inhibition and antibacterial activity

Table S1. Enzyme inhibition and antibacterial activity for type II compounds **7j**, **7l**, **7m-p**, **7r**, **7s**, **8a-c**, and **14**.

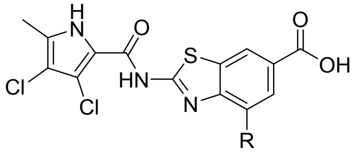

ID	7j	7l	7m	7n	7o	7p	7r	7s	8a	8b	8c	14	E
R =	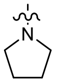	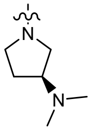 × HCl	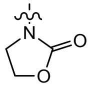	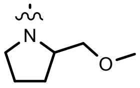	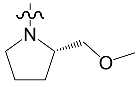	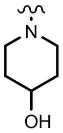	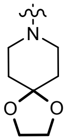	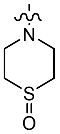	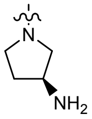 × HCl	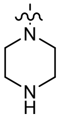 × HCl	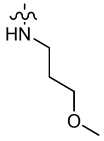	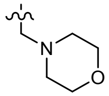 × HCl	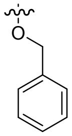
**Enzyme**						IC$_{50}$ [nM][a]							
E. coli gyrase	<10	<10	<10	14 ± 6	25 ± 0	<10	17 ± 11	<10	<10	<10	15 ± 5	13 ± 5	<10
E. coli topo IV	68 ± 16	210 ± 80	180 ± 16	210 ± 7	280 ± 14	140 ± 3	nt[b]	72 ± 3	38 ± 10	120 ± 15	190 ± 6	460 ± 120	350 ± 50
**Species**						MIC [µg/mL][c]							
Gram-positive bacteria													
S. aureus	0.25	4	64	0.0625	0.0625	4	<0.03125	>64	32	32	<0.03125	16	0.5
S. aureus (MRSA)	0.125	2	64	0.0625	0.0625	4	<0.03125	64	16	nt	<0.03125	nt	0.0625
S. aureus (VISA)	0.0625	0.5	8	0.0625	0.0625	0.5	<0.03125	>64	8	nt	nt	nt	<0.03125
E. faecalis	0.125	1	4	<0.03125	<0.03125	<0.03125	<0.03125	16	32	nt	>0.03125	nt	<0.03125
E. faecium	0.25	0.25	4	<0.03125	<0.03125	<0.03125	<0.03125	16	32	nt	nt	nt	0.0625
Gram-negative bacteria													
E. coli	16	>64	>64	8	16	>64	4	>64	>32	16	8	>64	16
P. aeruginosa	4	>64	>64	8	16	>64	8	>64	>32	>64	8	>64	2
A. baumannii	4	>64	>64	8	16	>64	8	>64	>32	64	2	>64	2
K. pneumoniae	8	>64	32	8	16	4	2	32	4	64	2	64	4
Enterobacter cloacae spp. *cloacae*	>32	nt	>64	>64	>64	>64	32	>64	>32	nt	>64	nt	>64

[a]IC$_{50}$, concentration (mean ± SD of three independent experiments) that inhibits enzyme activity by 50%.

[b]nt, not tested

[c]MIC, minimum inhibitory concentration

Bacterial strains that were used: *S. aureus* ATCC 29213, *S. aureus* (MRSA) ATCC 43300, *S. aureus* (VISA) ATCC 700699, *E. faecalis* ATCC 29212, *E. faecium* ATCC 700221, *E. coli* ATCC 25922, *P. aeruginosa* ATCC 27853 (PAO1 for compounds **7l**, **8b**, **14**), *A. baumannii* ATCC 17978 (ATCC 19606 for compounds **7l**, **8b**, **14**), *K. pneumoniae* ATCC 10031 (ATCC 13883 for compounds **7l**, **8b**, **14**), and *Enterobacter cloacae* spp. *cloacae* ATCC 13047.

Measurements were performed according to the Clinical and Laboratory Standards Institute guidelines, with three independent measurements.

Table S2. Enzyme inhibition and antibacterial activity for type III morpholino analogs **16a-c** with pyrrole substitutions.

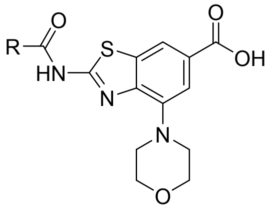

16a-c

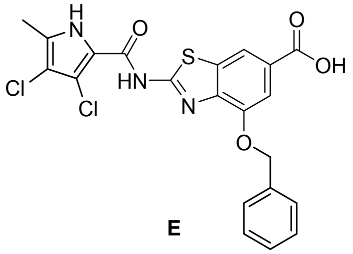

E

ID	16a	16b	16c	E
R =	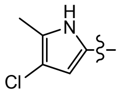	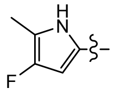	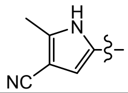	
**Enzyme**		**IC_{50} [nM]**[a]		
E. coli gyrase	13 ± 5	31 ± 6	32 ± 9	<10
E. coli topo IV	38 ± 10	120 ± 15	440 ± 10	350 ± 50
**Species**		**MIC [µg/mL]**[b]		
**Gram-positive bacteria**				
S. aureus	8	64	>64	0.5
S. aureus (MRSA)	2	32	nt[c]	0.0625
S. aureus (VISA)	0.5	8	nt	<0.03125
E. faecalis	1	16	nt	<0.03125
E. faecium	1	2	nt	0.0625
**Gram-negative bacteria**				
E. coli	>64	>64	>64	16
P. aeruginosa	64	>64	>64	2
A. baumannii	>64	>64	>64	2
K. pneumoniae	2	>64	>64	4
Enterobacter cloacae spp. *cloacae*	>64	nt	nt	>64

[a]IC_{50}, concentration (mean ± SD of three independent experiments) that inhibits enzyme activity by 50%.

[b]MIC, minimum inhibitory concentration

[c]nt, not tested

Bacterial strains that were used: *S. aureus* ATCC 29213, *S. aureus* (MRSA) ATCC 43300, *S. aureus* (VISA) ATCC 700699, *E. faecalis* ATCC 29212, *E. faecium* ATCC 700221, *E. coli* ATCC 25922, *P. aeruginosa* ATCC 27853 (PAO1 for compounds **16b** and **16c**), *A. baumannii* ATCC 17978 (ATCC 19606 for compounds **16b** and **16c**), *K. pneumoniae* ATCC 10031 (ATCC 13883 for compounds **16b** and **16c**), and *Enterobacter cloacae* spp. *cloacae* ATCC 13047.

Measurements were performed according to the Clinical and Laboratory Standards Institute guidelines, with three independent measurements.

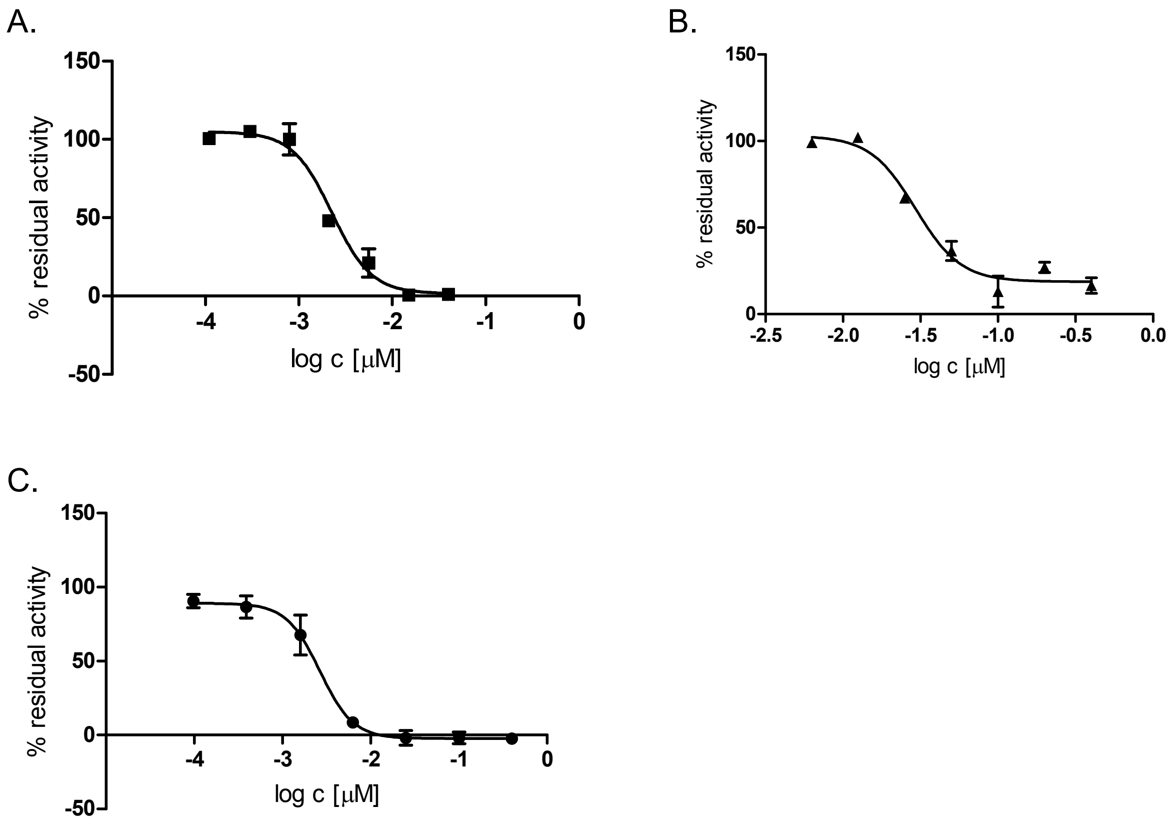

Figure S1. Dose-response curve for **7a** against *E. coli* DNA gyrase (A.), *E. coli* topo IV (B.), and *P. aeruginosa* DNA gyrase (C.) in supercoiling (for DNA gyrase) and relaxation (for topo IV) HTS assay, shown for an independent measurement in triplicate. The IC_{50} (mean ±SD) is the result of three independent measurements.

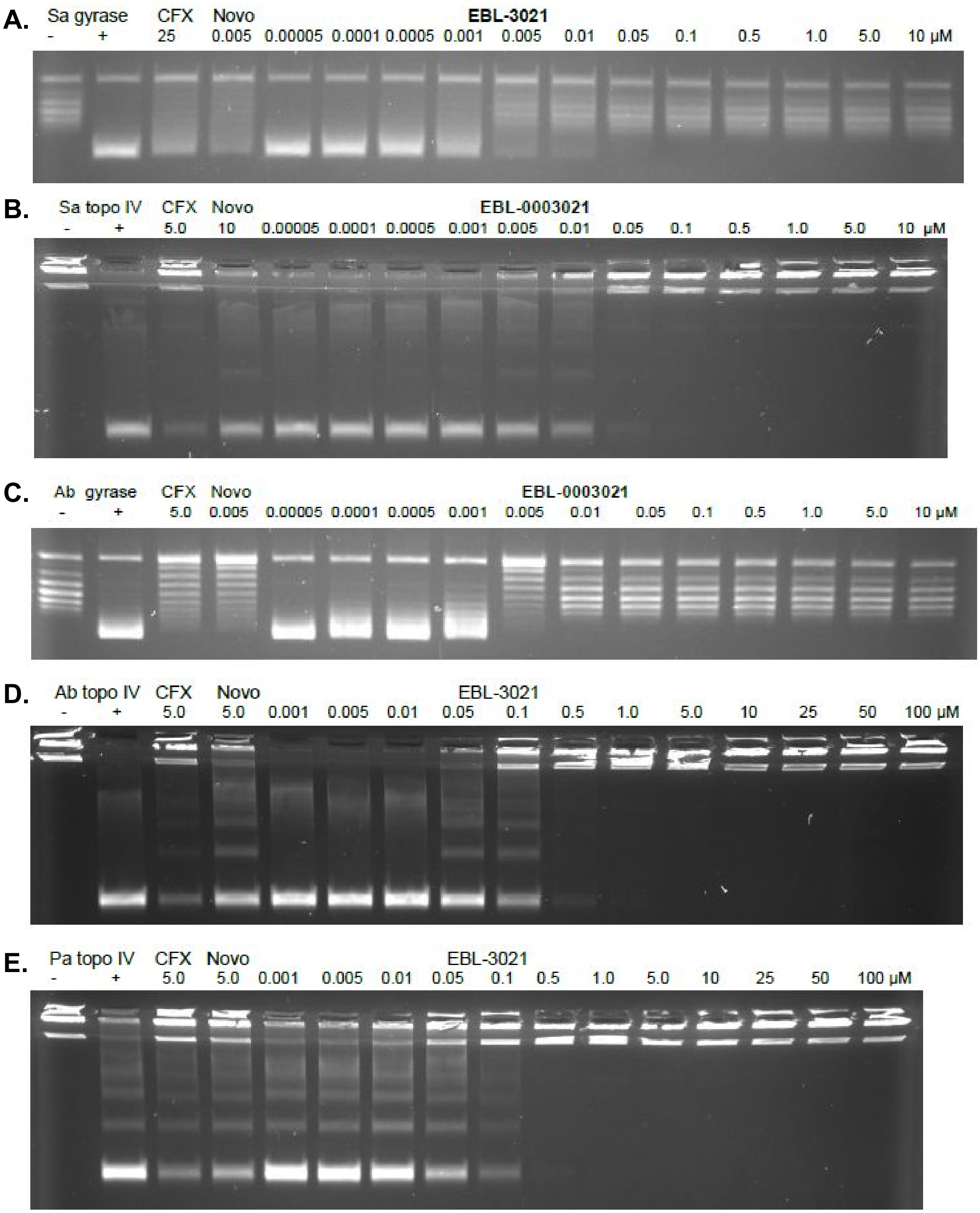

Figure S2. Images of gels for **7a** against *S. aureus* DNA gyrase (A.), *S. aureus* topo IV (B.), *A. baumannii* DNA gyrase (C.), *A. baumannii* topo IV (D.), and *P. aeruginosa* topo IV (E.) in supercoiling (for DNA gyrase) and decatenation (for topo IV) assay, shown for an independent experiment.

Thermodynamic solubility and plasma protein binding

Table S3. Thermodynamic solubility[a]

Compound ID	Thermodynamic solubility (pH = 7.4) [µM]
**E**	6.6
**7a**	98.3 (167.9)*
**7b**	68.1
**7c**	10.1
**7d**	5.8
**7e**	80.4
**7f**	0.92
**7g**	0.26
**7h**	3.8
**7i**	10.7
**7n**	11.2
**7o**	11.0
**7p**	183.7
**7r**	27.0
**16a**	137.6
**16b**	131.4

[a]Thermodynamic solubility in PBS buffer (pH = 7.4). The measurement was performed in two parallels, and the results are presented as means. Result of each independent experiment is available in Table S25.

*Thermodynamic solubility of **7a** was determined in two independent assays. The value in the brackets is from the second assay which was not used for other compounds.

Table S4. Plasma protein binding (mouse) of compound **E** and type I compounds **7a-d** and **7g-i**.[a]

Compound ID	E	7a	7b	7c	7d	7g	7h	7i
**fu [%]**[b]	<0.1	1.8	1.3	1.6	1.1	0.4	0.9	<0.1
**Recovery [%]**	94.1	81.5	84.5	93.0	89.5	93.5	89.0	109.8

[a]Male CD-1 mice. Results are means of two measurements.

[b]fu, fraction unbound.

In-depth microbiological profiling of 7a and 7h

Table S5. Frequency-of-resistance of *S. aureus* MRSA and VISA strains after exposure to increasing concentrations of **7a** or novobiocin.

	MIC [µg/mL] (wild-type strains)		Multiple of MIC	Frequency-of-resistance	
	**7a**	novobiocin		**7a**	novobiocin
***S. aureus* ATCC 43300 (MRSA)**	0.0625	0.125	2x	1.0 ± 0 (× 10^{-6})	1.5 ± 0.43 (× 10^{-8})
			4x	6.8 ± 4.5 (× 10^{-7})	1.1 ± 0.38 (× 10^{-8})
			8x	4.4 ± 5.9 (× 10^{-9})	9.3 ± 3.5 (× 10^{-9})
			20x	5.0 ± 0 (× 10^{-12})	9.0 ± 4.5 (× 10^{-10})
***S. aureus* ATCC 700699 (VISA)**	<0.03125	0.03125	2x	3.3 ± 4.7 (× 10^{-7})	2.5 ± 0.24 (× 10^{-8})
			4x	2.9 ± 4.4 (× 10^{-9})	1.2 ± 0.21 (× 10^{-8})
			8x	1.3 ± 1.7 (× 10^{-9})	7.5 ± 1.6 (× 10^{-9})
			20x	5.1 ± 0.12 (× 10^{-12})	5.3 ± 1.3 (× 10^{-9})

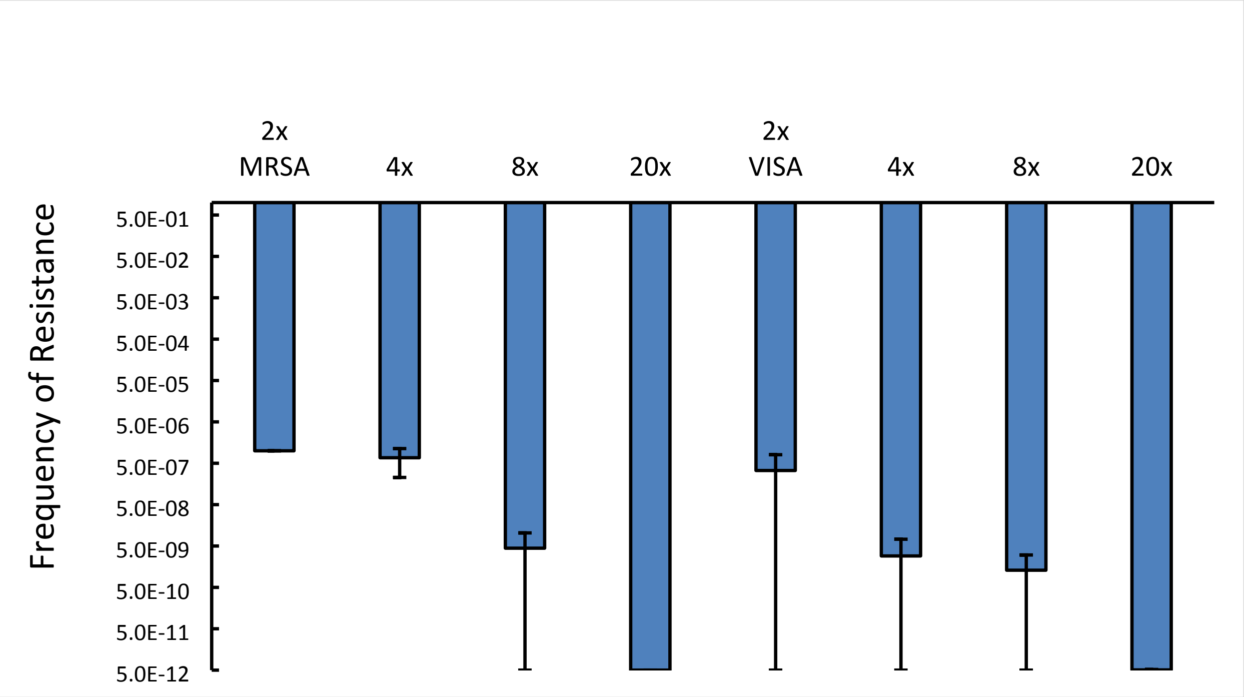

Figure S3. Frequency-of-resistance of *S. aureus* MRSA (ATCC 43300) and VISA (ATCC 700699) against **7a**.

Table S6. MIC values of **7a** and novobiocin against mutated MRSA and VISA strains.

	MIC [µg/mL] for the mutated, resistant strains		MIC fold change, compared to the wild type	
	**7a**	novobiocin	**7a**	novobiocin
S. aureus **ATCC 43300 (MRSA)**	2	16	32	128
S. aureus **ATCC 700699 (VISA)**	0.125	4	4	128

Table S7. Antibacterial activity of **7a** an **7h** against 3 sensitive and 7 MDR *A. baumannii* clinical isolates. Specific antibiotic resistance for each strain is shown in Figure S3.

**Species (strain + comment)**	**MIC [µg/mL][a]**	
	**7a**	**7h**
A. baumannii wt[b] 2/30	2	4
A. baumannii wt 2/1	2	4
A. baumannii wt 1/96	2	8
A. baumannii MDR[c] SZTE-ACI9	2	8
A. baumannii MDR SZTE-ACI4 (colistin-resistant; PDR[d])	2	4
A. baumannii MDR SZTE-ACI3 (colistin-resistant; PDR)	2	8
A. baumannii MDR SZTE-ACI2 (colistin-resistant)	2	8
A. baumannii MDR ACI75	2	8
A. baumannii MDR ACI100	4	8
A. baumannii MDR 2/2	4	8

[a]MIC, minimum inhibitory concentration

[b]wt, wild-type

[c]MDR, multidrug-resistant

[d]PDR, pandrug-resistant

Table S8. Cumulative % at MIC data for *A. baumannii* tested (n = 10)

	MIC (µg/mL)																	
**Compound**	**0**	**0.0039**	**0.0078**	**0.016**	**0.0313**	**0.0625**	**0.125**	**0.25**	**0.5**	**1**	**2**	**4**	**8**	**16**	**32**	**64**	**128**	**256**
**7a**											80	100						
**7h**												40	100					
meropenem								10		30					40	100		
imipenem									20	30						60	80	100
Imipenem/ relebactame									20		30					70	90	100
Ciprofloxacin									20	30			100					
Cefepime													20			100		
Levofloxacin						30							40	70	80	100		
Moxifloxacin						20			30				40		70	100		
Doxycycline								30			40	60	70			100		
Colistin										50	70	100						
Doripenem						10		20			30						60	100
Amikacin											10	20	30	40				100
Tobramycin										10	20	40	60	70				100
Trimethoprim/ sulfamethoxazole														20	70	100		

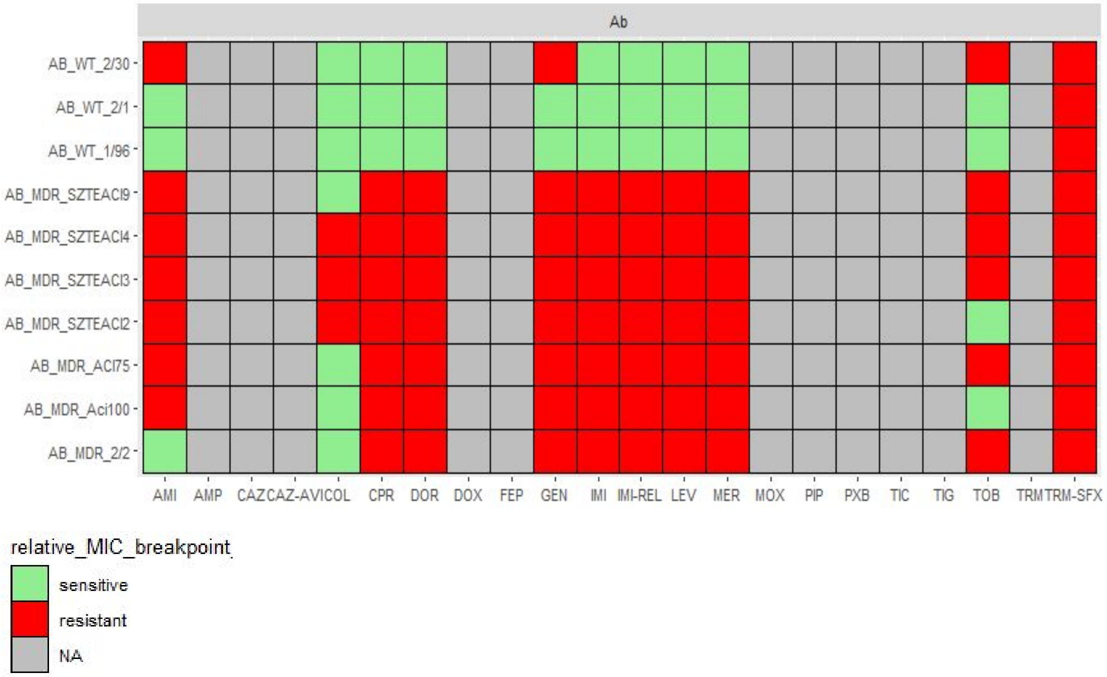

Figure S4. Resistance of each *A. baumannii* strain from Tables S5 and S6 against specific antimicrobial agent. A square is colored red if the MIC of the strain reached the EUCAST clinical breakpoint of the given antibiotic and green if not.

Table S9. Antibacterial activity of **7a** against *E. coli*, *A. baumannii* and *P. aeruginosa* strains.

**Species (strain + comment)**	**MIC [µg/mL]**[a]
E. coli ATCC 25922	8
E. coli CH3130 ΔtolC[b]	<0.125
E. coli CGSC 5163 D22[c]	2
P. aeruginosa PAO1	>64
P. aeruginosa PAO750[d]	0.25
A. baumannii BM4652	2
A. baumannii BM4652[e]	<0.125
**A. baumannii* NMI 2692/14	1
**A. baumannii* NMI 2699/14	1
**A. baumannii* NMI 2704/14	1
**A. baumannii* NMI 2715/14	4

[a]MIC, minimum inhibitory concentration

[b]efflux-defective (isogenic to *E. coli* ATCC 25922)

[c]lps mutant, hypersensitive

[d]efflux-defective (isogenic to *P. aeruginosa* PAO1)

[e]efflux-defective

*clinical multidrug-resistant (MDR) strain

Tables S10-S17 show the data on MIC distribution and cumulative % at MIC for compounds **7a**, **7h** and the control antibiotics against *N. gonorrhoeae*, *E. faecium*, *H. influenzae* and *S. pneumoniae*. The underlying data for these tables can be found in the excel file *Supplemental Materials_NG,EF,HI,SP_MIC*.

Table S10. MIC distribution against 12 *N. gonorrhoeae* tested

	MIC (µg/mL)																		
**Compound**	**0.004**	**0.015**	**≤0.03**	**0.03**	**0.06**	**0.12**	**≤0.25**	**0.25**	**≤0.5**	**0.5**	**1**	**2**	**4**	**8**	**>8**	**>16**	**32**	**>32**	**64**
**7a**			6		6														
**7h**			8		4														
Ciprofloxacin	5												3	2	2				
Cefpodoxime		1		1	3			2		1	2	2							
Penicillin								2		2	2	3	1			2			

Table S11. Cumulative % at MIC data for *N. gonorrhoeae* tested (n = 12)

	MIC (µg/mL)																		
**Compound**	**0.004**	**0.015**	**≤0.03**	**0.03**	**0.06**	**0.12**	**≤0.25**	**0.25**	**≤0.5**	**0.5**	**1**	**2**	**4**	**8**	**>8**	**>16**	**32**	**>32**	**64**
**7a**			50.0		100.0														
**7h**			66.7		100.0														
Ciprofloxacin	41.7												66.7	83.3	100.0				
Cefpodoxime		8.3		16.7	41.7			58.3		66.7	83.3	100.0							
Penicillin								16.7		33.3	50.0	75.0	83.3			100.0			

Table S12. MIC distribution against 13 *E. faecium* tested

	MIC (µg/mL)																		
**Compound**	**0.004**	**0.015**	**≤0.03**	**0.03**	**0.06**	**0.12**	**≤0.25**	**0.25**	**≤0.5**	**0.5**	**1**	**2**	**4**	**8**	**>8**	**16**	**32**	**>32**	**64**
**7a**					8	5													
**7h**			7		5	1													
Ampicillin																		13	
Levofloxacin																	2	11	
Penicillin																		13	
Imipenem																1		12	
Vancomycin																3	2	8	

Table S13. Cumulative % at MIC data for *E. faecium* tested (n = 13)

	MIC (µg/mL)																		
**Compound**	**0.004**	**0.015**	**≤0.03**	**0.03**	**0.06**	**0.12**	**≤0.25**	**0.25**	**≤0.5**	**0.5**	**1**	**2**	**4**	**8**	**>8**	**16**	**32**	**>32**	**64**
**7a**					61.5	100.0													
**7h**			53.8		92.3	100.0													
Ampicillin																		100.0	
Levofloxacin																	15.4	100.0	
Penicillin																		100.0	
Imipenem																7.7		100.0	
Vancomycin																23.1	38.5	100.0	

Table S14. MIC distribution against 14 *H. influenzae* tested

	MIC (μg/mL)																			
**Compound**	**0.0015**	**≤0.004**	**0.015**	**≤0.03**	**0.03**	**0.08**	**0.12**	**≤0.25**	**0.25**	**≤0.5**	**0.5**	**1**	**2**	**4**	**8**	**>8**	**16**	**32**	**>32**	**64**
**7a**							3		6		4	1								
**7h**							5		4		4	1								
Ampicillin													2	2	4		4	2		
Levofloxacin	1	1	10			1	1													
Penicillin														2	1		7	3	1	
Imipenem							2		1		9	2								
Vancomycin																		5	9	

Table S15. Cumulative % at MIC data for *H. influenzae* tested (n = 14)

	MIC (μg/mL)																			
**Compound**	**0.0015**	**≤0.004**	**0.015**	**≤0.03**	**0.03**	**0.08**	**0.12**	**≤0.25**	**0.25**	**≤0.5**	**0.5**	**1**	**2**	**4**	**8**	**>8**	**16**	**32**	**>32**	**64**
**7a**							21.4		64.3		92.9	100.0								
**7h**							35.7		64.3		92.9	100.0								
Ampicillin													14.3	28.6	57.1		85.7	100.0		
Levofloxacin	7.1	14.3	85.7			92.9	100.0													
Penicillin														14.3	21.4		71.4	92.9	100.0	
Imipenem							14.3		21.4		85.7	100.0								
Vancomycin																		35.7	100.0	

Table S16. MIC distribution against 12 *S. pneumoniae* tested

	MIC (µg/mL)																		
**Compound**	**≤0.004**	**≤0.015**	**≤0.03**	**0.03**	**0.06**	**0.12**	**≤0.25**	**0.25**	**≤0.5**	**0.5**	**1**	**2**	**4**	**8**	**>8**	**16**	**32**	**>32**	**64**
**7a**					1	1		1		9									
**7h**					2			9		1									
Ampicillin			1							2	1	4	2	2					
Levofloxacin										5	6							1	
Penicillin			1							2	2	5	2						
Imipenem		2			1	3		5			1								
Vancomycin						5		6			1								

Table S17. Cumulative % at MIC data for *S. pneumoniae* tested (n = 12)

	MIC (µg/mL)																		
**Compound**	**≤0.004**	**≤0.015**	**≤0.03**	**0.03**	**0.06**	**0.12**	**≤0.25**	**0.25**	**≤0.5**	**0.5**	**1**	**2**	**4**	**8**	**>8**	**16**	**32**	**>32**	**64**
**7a**					8.3	16.7		25.0		100.0									
**7h**					16.7			91.7		100.0									
Ampicillin			8.3							25.0	33.3	66.7	83.3	100.0					
Levofloxacin										41.7	91.7							100.0	
Penicillin			8.3							25.0	41.7	83.3	100.0						
Imipenem		16.7			25.0	50.0		91.7			100.0								
Vancomycin						41.7		91.7			100.0								

Selectivity and toxicity data

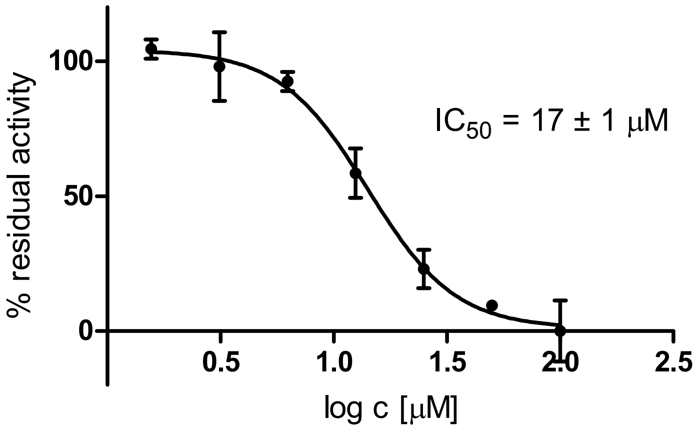

Figure S5. Dose-response curve for **7a** against human topoisomerase IIα in relaxation assay, shown for an independent measurement in triplicate. The IC_{50} (mean ±SD) is the result of three independent measurements.

Table S18. Selectivity against protein kinases. Residual activities (% of control) of 335 wild-type protein kinases after treatment with 1 µM or 10 µM concentration of **7a**. Highlighted are mean residual activities that are <50%.

#	Kinase Name	Kinase Family*	1 µM	10 µM
1	ABL1	TK	105	112
2	ABL2	TK	90	96
3	ACK1	TK	50	2
4	ACVR1	TKL	88	138
5	ACVR1B	TKL	88	106
6	ACVR2A	TKL	88	87
7	ACVR2B	TKL	52	2
8	ACVRL1	TKL	100	76
9	AKT1	AGC	90	106
10	AKT2	AGC	111	96
11	AKT3	AGC	113	104
12	ALK	TK	90	14
13	AMPKalpha1	CAMK	88	90
14	ARK5	CAMK	92	83
15	ASK1	STE	98	94
16	AuroraA	OTHER	87	39
17	AuroraB	OTHER	106	63
18	AuroraC	OTHER	99	56
19	AXL	TK	98	48
20	BLK	TK	74	19
21	BMPR1A	TKL	108	135
22	BMPR1B	TKL	98	100
23	BMX	TK	96	8
24	BRAF	TKL	92	69
25	BRK	TK	85	14
26	BRSK1	CAMK	99	100
27	BRSK2	CAMK	103	95
28	BTK	TK	98	21
29	BUB1B	OTHER	95	82
30	CAMK1D	CAMK	100	96
31	CAMK2A	CAMK	100	96
32	CAMK2B	CAMK	101	79
33	CAMK2D	CAMK	98	92
34	CAMK2G	CAMK	91	56
35	CAMK4	CAMK	103	87
36	CAMKK1	OTHER	98	28
37	CAMKK2	OTHER	97	90
38	CDC42BPA	AGC	97	92
39	CDC42BPB	AGC	103	60
40	CDC7/DBF4	OTHER	98	57
41	CDK1/CycA2	CMGC	105	96
42	CDK1/CycB1	CMGC	98	90
43	CDK1/CycE1	CMGC	90	90
44	CDK12/CycK	CMGC	107	108
45	CDK13/CycK	CMGC	105	84
46	CDK16/CycY	CMGC	98	104
47	CDK17/p35NCK	CMGC	94	79
48	CDK18/CycY	CMGC	102	94

**49**	**CDK19/CycC**	**CMGC**	95	75
**50**	**CDK2/CycA2**	**CMGC**	87	94
**51**	**CDK2/CycD1**	**CMGC**	101	111
**52**	**CDK2/CycE1**	**CMGC**	94	79
**53**	**CDK20/CycH**	**CMGC**	97	74
**54**	**CDK20/CycT1**	**CMGC**	104	83
**55**	**CDK3/CycC**	**CMGC**	105	77
**56**	**CDK3/CycE1**	**CMGC**	105	93
**57**	**CDK4/CycD1**	**CMGC**	92	92
**58**	**CDK4/CycD2**	**CMGC**	95	91
**59**	**CDK4/CycD3**	**CMGC**	95	84
**60**	**CDK5/p25NCK**	**CMGC**	96	77
**61**	**CDK5/p35NCK**	**CMGC**	99	97
**62**	**CDK6/CycD1**	**CMGC**	101	103
**63**	**CDK6/CycD2**	**CMGC**	92	75
**64**	**CDK6/CycD3**	**CMGC**	107	98
**65**	**CDK7/CycH/MAT1**	**CMGC**	104	91
**66**	**CDK8/CycC**	**CMGC**	98	51
**67**	**CDK9/CycK**	**CMGC**	95	88
**68**	**CDK9/CycT1**	**CMGC**	101	88
**69**	**CHK1**	**CAMK**	100	94
**70**	**CHK2**	**CAMK**	98	22
**71**	**CK1alpha1**	**CK1**	107	83
**72**	**CK1delta**	**CK1**	101	74
**73**	**CK1epsilon**	**CK1**	110	74
**74**	**CK1gamma1**	**CK1**	93	65
**75**	**CK1gamma2**	**CK1**	97	74
**76**	**CK1gamma3**	**CK1**	99	82
**77**	**CK2alpha1**	**OTHER**	98	60
**78**	**CK2alpha2**	**OTHER**	108	62
**79**	**CLK1**	**CMGC**	91	30
**80**	**CLK2**	**CMGC**	104	78
**81**	**CLK3**	**CMGC**	109	108
**82**	**CLK4**	**CMGC**	99	32
**83**	**COT**	**STE**	97	83
**84**	**CSF1R**	**TK**	67	11
**85**	**CSK**	**TK**	90	9
**86**	**DAPK1**	**CAMK**	102	103
**87**	**DAPK2**	**CAMK**	110	89
**88**	**DAPK3**	**CAMK**	95	102
**89**	**DCAMKL2**	**CAMK**	87	98
**90**	**DDR2**	**TK**	100	102
**91**	**DMPK**	**AGC**	93	21
**92**	**DNAPK**	**ATYPICAL**	97	68
**93**	**DYRK1A**	**CMGC**	96	84
**94**	**DYRK1B**	**CMGC**	105	86
**95**	**DYRK2**	**CMGC**	102	91
**96**	**DYRK3**	**CMGC**	98	75
**97**	**DYRK4**	**CMGC**	93	84
**98**	**EEF2K**	**ATYPICAL**	83	85
**99**	**EGFR**	**TK**	58	10
**100**	**EIF2AK2**	**OTHER**	82	16
**101**	**EIF2AK3**	**OTHER**	93	102
**102**	**EPHA1**	**TK**	93	41
**103**	**EPHA2**	**TK**	89	24
**104**	**EPHA3**	**TK**	91	35

**105**	**EPHA4**	**TK**	98	56
**106**	**EPHA5**	**TK**	49	14
**107**	**EPHA6**	**TK**	85	16
**108**	**EPHA7**	**TK**	84	19
**109**	**EPHA8**	**TK**	90	22
**110**	**EPHB1**	**TK**	63	3
**111**	**EPHB2**	**TK**	97	83
**112**	**EPHB3**	**TK**	82	5
**113**	**EPHB4**	**TK**	97	33
**114**	**ERBB2**	**TK**	89	72
**115**	**ERBB4**	**TK**	63	9
**116**	**ERK1**	**CMGC**	107	86
**117**	**ERK2**	**CMGC**	101	80
**118**	**ERK5**	**CMGC**	90	90
**119**	**ERK7**	**CMGC**	98	84
**120**	**FAK**	**TK**	103	60
**121**	**FER**	**TK**	37	11
**122**	**FES**	**TK**	84	18
**123**	**FGFR1**	**TK**	89	29
**124**	**FGFR2**	**TK**	64	3
**125**	**FGFR3**	**TK**	99	13
**126**	**FGFR4**	**TK**	93	33
**127**	**FGR**	**TK**	91	46
**128**	**FLT3**	**TK**	104	90
**129**	**FRK**	**TK**	69	24
**130**	**FYN**	**TK**	100	24
**131**	**GRK2**	**AGC**	91	105
**132**	**GRK3**	**AGC**	111	105
**133**	**GRK4**	**AGC**	102	62
**134**	**GRK5**	**AGC**	99	71
**135**	**GRK6**	**AGC**	112	84
**136**	**GRK7**	**AGC**	95	71
**137**	**GSG2**	**OTHER**	96	80
**138**	**GSK3alpha**	**CMGC**	108	93
**139**	**GSK3beta**	**CMGC**	99	81
**140**	**HCK**	**TK**	57	32
**141**	**HIPK1**	**CMGC**	97	82
**142**	**HIPK2**	**CMGC**	84	105
**143**	**HIPK3**	**CMGC**	83	79
**144**	**HIPK4**	**CMGC**	91	85
**145**	**HRI**	**OTHER**	96	85
**146**	**IGF1R**	**TK**	90	24
**147**	**IKKalpha**	**OTHER**	105	87
**148**	**IKKbeta**	**OTHER**	93	81
**149**	**IKKepsilon**	**OTHER**	93	86
**150**	**INSR**	**TK**	98	129
**151**	**INSRR**	**TK**	105	106
**152**	**IRAK1**	**TKL**	90	95
**153**	**IRAK4**	**TKL**	96	61
**154**	**ITK**	**TK**	93	37
**155**	**JAK1**	**TK**	91	93
**156**	**JAK2**	**TK**	97	83
**157**	**JAK3**	**TK**	102	87
**158**	**JNK1**	**CMGC**	99	80
**159**	**JNK2**	**CMGC**	84	86
**160**	**JNK3**	**CMGC**	108	107

**161**	**KIT**	**TK**	86	30
**162**	**LCK**	**TK**	93	35
**163**	**LIMK1**	**TKL**	88	85
**164**	**LIMK2**	**TKL**	97	88
**165**	**LRRK2**	**TKL**	91	58
**166**	**LTK**	**TK**	83	11
**167**	**LYN**	**TK**	67	17
**168**	**MAP3K1**	**STE**	99	27
**169**	**MAP3K10**	**STE**	96	57
**170**	**MAP3K11**	**STE**	75	69
**171**	**MAP3K7/MAP3K7IP1**	**STE**	96	72
**172**	**MAP3K9**	**STE**	103	77
**173**	**MAP4K2**	**STE**	99	49
**174**	**MAP4K4**	**STE**	108	86
**175**	**MAP4K5**	**STE**	103	88
**176**	**MAPKAPK2**	**CAMK**	95	71
**177**	**MAPKAPK3**	**CAMK**	87	35
**178**	**MAPKAPK5**	**CAMK**	103	74
**179**	**MARK1**	**CAMK**	95	90
**180**	**MARK2**	**CAMK**	105	93
**181**	**MARK3**	**CAMK**	100	89
**182**	**MARK4**	**CAMK**	103	95
**183**	**MASTL**	**AGC**	117	100
**184**	**MATK**	**TK**	88	14
**185**	**MEK1**	**STE**	115	84
**186**	**MEK2**	**STE**	107	93
**187**	**MEK5**	**STE**	91	84
**188**	**MEKK2**	**STE**	99	87
**189**	**MEKK3**	**STE**	94	86
**190**	**MELK**	**CAMK**	89	57
**191**	**MERTK**	**TK**	88	26
**192**	**MET**	**TK**	115	95
**193**	**MINK1**	**STE**	88	78
**194**	**MKK4**	**STE**	86	73
**195**	**MKK6 SDTD**	**STE**	96	108
**196**	**MKK7**	**STE**	97	82
**197**	**MKNK1**	**CAMK**	95	30
**198**	**MKNK2**	**CAMK**	97	78
**199**	**MLK4**	**TKL**	80	48
**200**	**MST1**	**STE**	99	97
**201**	**MST2**	**STE**	96	96
**202**	**MST3**	**STE**	86	81
**203**	**MST4**	**STE**	87	67
**204**	**MTOR**	**ATYPICAL**	87	77
**205**	**MUSK**	**TK**	101	81
**206**	**MYLK**	**CAMK**	118	11
**207**	**MYLK2**	**CAMK**	89	82
**208**	**MYLK3**	**CAMK**	104	113
**209**	**NEK1**	**OTHER**	82	79
**210**	**NEK11**	**OTHER**	105	91
**211**	**NEK2**	**OTHER**	91	83
**212**	**NEK3**	**OTHER**	101	90
**213**	**NEK4**	**OTHER**	99	77
**214**	**NEK6**	**OTHER**	103	109
**215**	**NEK7**	**OTHER**	109	93
**216**	**NEK9**	**OTHER**	101	84

**217**	**NIK**	**STE**	103	84
**218**	**NLK**	**CMGC**	95	96
**219**	**p38alpha**	**CMGC**	90	84
**220**	**p38beta**	**CMGC**	88	87
**221**	**p38delta**	**CMGC**	100	82
**222**	**p38gamma**	**CMGC**	96	90
**223**	**PAK1**	**STE**	101	25
**224**	**PAK2**	**STE**	83	22
**225**	**PAK3**	**STE**	86	26
**226**	**PAK4**	**STE**	103	63
**227**	**PAK6**	**STE**	101	109
**228**	**PAK7**	**STE**	98	34
**229**	**PASK**	**CAMK**	86	72
**230**	**PBK**	**OTHER**	75	84
**231**	**PDGFRalpha**	**TK**	99	92
**232**	**PDGFRbeta**	**TK**	103	99
**233**	**PDK1**	**AGC**	98	42
**234**	**PHKG1**	**CAMK**	111	56
**235**	**PHKG2**	**CAMK**	85	78
**236**	**PIM1**	**CAMK**	94	60
**237**	**PIM2**	**CAMK**	97	105
**238**	**PIM3**	**CAMK**	92	79
**239**	**PKA**	**AGC**	78	47
**240**	**PKCalpha**	**AGC**	97	82
**241**	**PKCbeta1**	**AGC**	92	57
**242**	**PKCbeta2**	**AGC**	108	71
**243**	**PKCdelta**	**AGC**	107	86
**244**	**PKCepsilon**	**AGC**	83	94
**245**	**PKCeta**	**AGC**	109	93
**246**	**PKCgamma**	**AGC**	104	78
**247**	**PKCiota**	**AGC**	96	90
**248**	**PKCmu**	**AGC**	96	87
**249**	**PKCnu**	**AGC**	93	59
**250**	**PKCtheta**	**AGC**	89	79
**251**	**PKCzeta**	**AGC**	98	109
**252**	**PKMYT1**	**OTHER**	90	82
**253**	**PKMzeta**	**AGC**	102	75
**254**	**PKN3**	**AGC**	104	42
**255**	**PLK1**	**OTHER**	105	89
**256**	**PLK3**	**OTHER**	101	77
**257**	**PRK1**	**AGC**	98	120
**258**	**PRK2**	**AGC**	86	95
**259**	**PRKD2**	**CAMK**	93	95
**260**	**PRKG1**	**AGC**	96	50
**261**	**PRKG2**	**AGC**	94	95
**262**	**PRKX**	**AGC**	92	78
**263**	**PYK2**	**TK**	108	58
**264**	**RAF1 YDYD**	**TKL**	76	91
**265**	**RET**	**TK**	84	17
**266**	**RIPK2**	**TKL**	85	84
**267**	**RIPK4**	**TKL**	95	92
**268**	**RIPK5**	**TKL**	87	88
**269**	**ROCK1**	**AGC**	90	30
**270**	**ROCK2**	**AGC**	99	86
**271**	**RON**	**TK**	81	15
**272**	**ROS**	**TK**	102	104

**273**	**RPS6KA1**	**AGC**	117	99
**274**	**RPS6KA2**	**AGC**	102	50
**275**	**RPS6KA3**	**AGC**	105	47
**276**	**RPS6KA4**	**AGC**	95	89
**277**	**RPS6KA5**	**AGC**	89	90
**278**	**RPS6KA6**	**AGC**	91	89
**279**	**S6K**	**AGC**	99	92
**280**	**S6Kbeta**	**AGC**	80	86
**281**	**SAK**	**OTHER**	89	70
**282**	**SGK1**	**AGC**	92	56
**283**	**SGK2**	**AGC**	97	89
**284**	**SGK3**	**AGC**	101	89
**285**	**SIK1**	**CAMK**	96	94
**286**	**SIK2**	**CAMK**	99	88
**287**	**SIK3**	**CAMK**	94	87
**288**	**SLK**	**STE**	87	72
**289**	**SNARK**	**CAMK**	107	76
**290**	**SNK**	**OTHER**	100	93
**291**	**SRC**	**TK**	91	20
**292**	**SRMS**	**TK**	98	55
**293**	**SRPK1**	**CMGC**	108	72
**294**	**SRPK2**	**CMGC**	98	40
**295**	**STK17A**	**CAMK**	106	82
**296**	**STK23**	**CAMK**	98	70
**297**	**STK25**	**STE**	100	76
**298**	**STK33**	**CAMK**	91	93
**299**	**STK39**	**STE**	97	78
**300**	**SYK**	**TK**	96	25
**301**	**TAOK2**	**STE**	90	122
**302**	**TAOK3**	**STE**	119	88
**303**	**TBK1**	**OTHER**	96	81
**304**	**TEC**	**TK**	90	57
**305**	**TGFBR1**	**TKL**	95	81
**306**	**TGFBR2**	**TKL**	88	56
**307**	**TIE2**	**TK**	53	9
**308**	**TLK1**	**AGC**	98	66
**309**	**TLK2**	**AGC**	100	50
**310**	**TNK1**	**TK**	79	16
**311**	**TRKA**	**TK**	87	51
**312**	**TRKB**	**TK**	51	9
**313**	**TRKC**	**TK**	89	35
**314**	**TSF1**	**OTHER**	102	92
**315**	**TSK2**	**CAMK**	88	92
**316**	**TSSK1**	**CAMK**	100	79
**317**	**TTBK1**	**CK1**	106	56
**318**	**TTBK2**	**CK1**	110	89
**319**	**TTK**	**OTHER**	97	84
**320**	**TXK**	**TK**	112	99
**321**	**TYK2**	**TK**	94	97
**322**	**TYRO3**	**TK**	94	8
**323**	**ULK2**	**OTHER**	104	101
**324**	**VEGFR1**	**TK**	70	23
**325**	**VEGFR2**	**TK**	97	57
**326**	**VEGFR3**	**TK**	74	26
**327**	**VRK1**	**CK1**	103	103
**328**	**VRK2**	**CK1**	113	106

**329**	**WEE1**	**OTHER**	100	93
**330**	**WNK1**	**OTHER**	112	93
**331**	**WNK2**	**OTHER**	90	87
**332**	**WNK3**	**OTHER**	92	75
**333**	**YES**	**TK**	84	21
**334**	**ZAK**	**TKL**	98	56
**335**	**ZAP70**	**TK**	91	14
**Selectivity score (< 50% residual activity):**			0.006	0.233

*Classification of protein kinase families (Manning et al. Science 6 December 2002: Vol. 298 no. 5600 pp. 1912-1934):
AGC: containing PKA, PKG and PKC families
CAMK: Calcium/Calmoduline-dependent protein kinases
CK1: Casein kinase 1 -like
CMGC: containing CDK, MAPK, GSK3 and CLK families
TK: Tyrosine Kinase
TKL: Tyrosine Kinase-like
STE: Homologs of Yeast Sterile 7, Sterile 11, Sterile 20 Kinases
MKK6 SDTD, RAF1 YDYD: Constitutively active variants

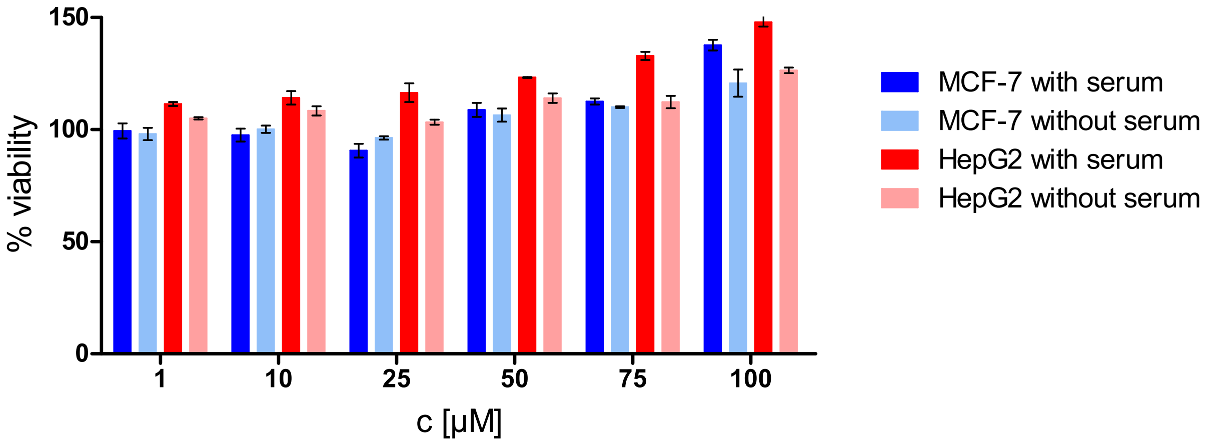

Figure S6. Cell viability. Results for MCF-7 and HepG2 cells in the MTS assay after treatment with compound **7a** at six concentrations. Data are means ±SD of three independent experiments, each performed in triplicate.

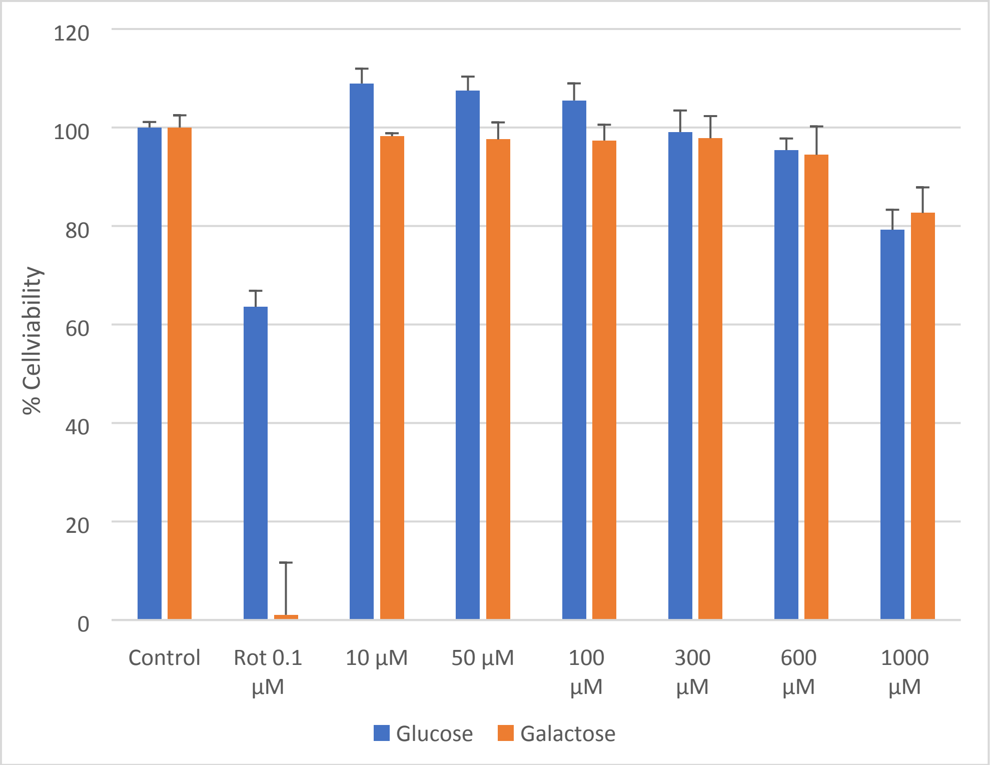

Figure S7. Mitochondrial toxicity (mean ±SD) for **7a** in glu/gal assay on HepG2 cells. Rotenone was used as positive control and compounds were assayed in six replicates.

Table S19. Mitochondrial toxicity data for compound **7a**.

		Control	Rot 0.1 µM	10 µM	50 µM	100 µM	300 µM	600 µM	1000 µM
**Glucose**	**Mean**	2170065	1379493.7	2363964	2332689	2289184	2150040	2070581	1719436
	**SD**	24285.73	45213.754	71216.62	66290.64	79431.71	94057.14	48691.95	69926.07
	**%**	100	64	109	107	105	99	95	79
	**%SD**	1	3	3	3	3	4	2	4
	**Mitotox ratio**			**1.1**	**1.1**	**1.1**	**1.0**	**1.0**	**1.0**
		**Control**	**Rot 0.1 µM**	**10 µM**	**50 µM**	**100 µM**	**300 µM**	**600 µM**	**1000 µM**
**Galactose**	**Mean**	2246116	23594.833	2206371	2191828	2186714	2197021	2121364	1856148
	**SD**	55665.5	2502.6693	13306.33	75731.22	70204.36	98948.15	122557	96631.28
	**%**	100	1	98	98	97	98	94	83
	**%SD**	2	11	1	3	3	5	6	5

Table S20. Genotoxicity assay. Results for **7a** and positive controls mitomycin C and benzo[*a*]pyrene in micronucleus test in Chinese hamster ovary-K1 cells without and with metabolic activation by a rat liver S9 fraction. **7a** was assayed at five different concentrations (without S9) and three different concentrations (with S9) and the results are calculated in terms of number of binucleate cells, cytostasis percentage and percentage of micronuclei in binucleate cells. Compounds were assayed in six replicates.

**Assay**	**Compound**	**c [µM]**	**No. binucleate cells**	**% Cytostasis**	**% Micronuclei**
Without S9	Control		2563	-	2.5
	**7a**	6.25	3160	-	2.8
		12.5	2783	-	2.7
		25	2644	-	2.5
		50	1687	0.1	4.4
		100	642	40.8	7
	Mitomycin C	1	920	41.6	23.3
With S9	Control		1913	-	2.9
	**7a**	25	1707	-	2.7
		50	1296	1.4	4.5
		100	-	tox	-
	Benzo[*a*]pyrene	150	1702	8.3	16.9

Table S21. Cardiotoxicity and haemolytic activity of **7a**.

**Na_v1.5 current fraction at 10 µM 7a (normalized to neg. control) (mean ± SEM)**	0.97 ± 0.03
**hERG current fraction at 50 µM 7a (normalized to neg. control) (mean ± SEM)**	1.01 ± 0.02
**hERG inhibition at 50 µM 7a (mean ± SD [%])**	0 ± 12
**Haemolysis at 100 µM 7a [%]**	0.28 ± 0.01

In vivo and formulation studies

Formulation study

Table S22. Formulation study data for inhibitor **7a**.

**X-Ray Powder Diffraction (XRPD)**	crystalline
**melting point - Differential Scanning Calorimetry (DSC)**	331 °C
**Solubility** (100 mM carbonate buffer, pH 9.0, with 20% CD – kleptose), osmolality **511** mOsm/kg (target 280-600 mOsm/kg)	13.2 mg/mL (target >10 mg/mL)
**Solubility** (100 mM carbonate buffer, pH 8.0, with 20% CD – kleptose), osmolality **245** mOsm/kg (target 280-600 mOsm/kg)	3.8 mg/mL

In vivo assay

Table S23. Data for Figure 7 featured in the manuscript.

						**Change in log_{10}CFU/gr of thigh from**	
**ID**	**Dose [mg/kg]**	**Total dose [mg/kg]**	**Route/regimen**	**Average log_{10}CFU/ gr of thigh**	**SD**	**2 hour control**	**26 hours control**
**2 hr infection control**	n/a	n/a	n/a	6.29	0.15		-1.80
**26 hr infection control**	n/a	n/a	n/a	8.10	0.23	1.80	
**7a**	10	30	TID, IV	8.29	0.11	2.00	0.19
	25	75	TID, IV	6.63	0.69	0.33	-1.47
	50	150	TID, IV	5.34	0.43	-0.96	-2.76
**Linezolid**	50	100	BID/SC	5.69	0.29	-0.61	-241

Table S24. Underlying raw data for Figure 7 featured in the manuscript and for Table S23. Four animals were dosed per group.

Group ID	CFU/ g of thigh	log CFU	Average/gr	S.D.
**2 hr. Infection Control**	1.62E+06	6.21	6.29	0.15
	2.13E+06	6.33		
	1.41E+06	6.15		
	3.10E+06	6.49		
**7a 10 mg/kg**	1.82E+08	8.26	8.29	0.11
	2.23E+08	8.35		
	2.51E+08	8.40		
	1.42E+08	8.15		
**7a 25 mg/kg**	4.64E+05	5.67	6.63	0.69
	4.16E+06	6.62		
	1.83E+07	7.26		
	9.23E+06	6.97		
**7a 50 mg/kg**	1.72E+05	5.24	5.34	0.43
	1.68E+05	5.23		
	8.81E+05	5.95		
	8.82E+04	4.95		
**Linezolid**	7.27E+05	5.86	5.69	0.29
	7.86E+05	5.90		
	5.42E+05	5.73		
	1.84E+05	5.26		
**26 hr. Infection Control**	6.20E+07	7.79	8.10	0.23
	1.97E+08	8.29		
	1.90E+08	8.28		
	1.56E+08	8.19		
	8.45E+07	7.93		

Determination of inhibitory activities on *S. aureus* and *A. baumannii* DNA gyrase and topoisomerase IV and *P. aeruginosa* topoisomerase IV

7a was tested for its inhibitory activities against *S. aureus* and *A. baumannii* DNA gyrase and topoisomerase IV and *P. aeruginosa* topoisomerase IV. In all experiments, the activity of the enzymes was determined prior to the testing of the compound and 1 unit (U) was defined as the amount of enzyme required to just fully supercoil or decatenate the substrate. This amount of enzyme was initially used in determination of control inhibitor activity. These experiments were performed in duplicate. Compound **7a** was dissolved in 100% (v/v) DMSO. The final DMSO concentration was 1% for all assays. All measurements were performed in quadruplicates. The condition for each reaction were as follows:

– ***S. aureus* gyrase supercoiling assay**

1 U of *S. aureus* gyrase was incubated with 0.5 μg of relaxed pBR322 DNA in a 30 μL reaction at 37 °C for 30 minutes under the following conditions: 40 mM HEPES-KOH (pH 7.6), 10 mM magnesium acetate, 10 mM DTT, 2 mM ATP, 500 mM potassium glutamate and 0.05 mg/mL BSA.

– ***S. aureus* topo IV decatenation assay**

1 U of *S. aureus* topo IV was incubated with 20 μg kDNA in a 30 μL reaction at 37 °C for 30 minutes under the following conditions: 50 mM Tris-HCl (7.5), 5 mM $MgCl_2$, 5 mM DTT, 1.5 mM ATP, 350 mM potassium glutamate and 0.05 mg/mL albumin.

– ***A. baumannii* gyrase supercoiling assay**

1 U of *A. baumannii* gyrase was incubated with 0.5 μg of relaxed pBR322 DNA in a 30 μL reaction at 37 °C for 30 minutes under the following conditions: 35 mM Tris-HCl (pH 7.5), 24 mM KCl, 4 mM $MgCl_2$, 2 mM DTT, 1.8 mM spermidine, 1 mM ATP, 6.5 % (w/v) glycerol and 0.1 mg/mL albumin.

– ***P. aeruginosa* and *A. baumannii* topo IV decatenation assay**

1 U of *P. aueruginosa* or *A. baumannii* topo IV was incubated with 0.2 μg of kDNA in a 30 μL reaction at 37 °C for 30 minutes under the following conditions: 50 mM HEPES-KOH (pH 7.9), 6 mM magnesium acetate, 4 mM DTT, 1 mM ATP, 100 mM potassium glutamate, 2 mM spermidine and 0.05 mg/mL albumin.

Each reaction was stopped by the addition of 30 μL chloroform/iso-amyl alcohol (24:1) and 30 μL Stop Dye (40% sucrose (w/v), 100 mM Tris-HCl (pH 7.5), 10 mM EDTA, 0.5 μg/mL bromophenol blue), before being loaded on a 1.0% TAE gel run at 80V for 2 hours. Bands were visualised by ethidium bromide staining for 20 minutes and destaining for 20 minutes. Gels were scanned using documentation equipment (GeneGenius, Syngene, Cambridge, UK) and % inhibition levels were measured by determining the relative fluorescence of the supercoiled band using gel scanning software. (GeneTools, Syngene, Cambridge, UK).

Protein kinase assay

The kinase inhibition profile of compound **7a** was tested at 10 μM and 1 μM concentration in singlicate. Compound was dissolved in DMSO and the final DMSO concentration in all reaction cocktails (including high and low controls) was 1 %. All kinase assays were performed in 96-well FlashPlatesTM from Perkin Elmer (Boston, MA, USA) in a 50 μL reaction volume. The reaction cocktail was pipetted in 4 steps in the following order: 10 μL of non-radioactive ATP solution (in H_2O), 25 μL of assay buffer/ [γ-33P]-ATP mixture, 5 μL of test sample in 10% DMSO, and 10 μL of enzyme/substrate mixture. The assay for all protein kinases contained 70 mM HEPES-NaOH pH 7.5, 3 mM MgCl2, 3 mM $MnCl_2$, 3 μM Na-orthovanadate, 1.2 mM DTT, 50 μg/ml PEG20000, ATP (variable amounts, corresponding to the apparent ATP-Km of the respective kinase), [γ-33P]-ATP (approx. 8 × 1005 cpm per well), protein kinase, and substrate.

The concentrations of enzymes and substrates as well as the ATP-concentrations used are shown for each assay in the table *Assay conditions* in *Supplemental Materials_kinase_assay* file.

All PKC assays (except the PKC-mu and the PKC-nu assay) additionally contained 1 mM $CaCl_2$, 4 mM EDTA, 5 μg/ml Phosphatidylserine and 1 μg/ml 1,2-Dioleyl-glycerol. The CAMK1D, CAMK2A, CAMK2B, CAMK2D, CAMK2G, CAMK4, CAMKK1, CAMKK2, DAPK2, EEF2K, MYLK, MYLK2 and MYLK3 assays additionally contained 1 μg/ml Calmodulin and 0.5 mM $CaCl_2$. The PRKG1 and PRKG2 assays additionally contained 1 μM cGMP. The DNA-PK assay additionally contained 2.5 μg/ml DNA.

The protein kinase reaction cocktails were incubated at 30 °C for 60 minutes. The reaction was stopped with 50 μL of 2% (v/v) H_3PO_4, plates were aspirated and washed two times with 200 μL 0.9 % (w/v) NaCl. Incorporation of 33Pi (counting of "cpm") was determined with a microplate scintillation counter (Microbeta, Wallac). All protein kinase assays were performed with a BeckmanCoulter Biomek 2000/SL robotic system.

All protein kinases provided by ProQinase were expressed in Sf9 insect cells or in E. coli as recombinant GST-fusion proteins or His-tagged proteins, either as full-length or enzymatically active fragments. All kinases were produced from human cDNAs and purified by either GSH-affinity chromatography or immobilized metal. Affinity tags were removed from a number of kinases during purification. The purity of the protein kinases was examined by SDS-PAGE/Coomassie staining, the identity was checked by mass spectroscopy.

Kinases from external vendors (CAR = Carna Biosciences Inc.; INV = Life Technologies (Invitrogen Corporation); MIL = Merck-Millipore (Millipore Corporation)) were expressed, purified and quality-controlled by virtue of the vendors readings.

Calculation of Residual Activities

For each kinase, the median value of the cpm of three wells was defined as **"low control"** (n=3). This value reflects unspecific binding of radioactivity to the plate in the absence of a protein kinase but in the presence of the substrate. Additionally, for each kinase the median value of the cpm of three other wells was taken as the **"high control"**, i.e. full activity in the absence of any inhibitor (n=3). The difference between high and low control of each enzyme was taken as 100% activity.

As part of the data evaluation the low control of each kinase was subtracted from the high control value as well as from their corresponding "compound values". The residual activity (in %) for each compound well was calculated by using the formula (F1):

Res. Activity (%) = 100 × [(signal of compound – low control) / (high control – low control)] (F1)

Calculation of Selectivity Scores

The selectivity score, according to Karaman et al.,[1] is a compound concentration-dependent parameter describing the portion of kinases, which are inhibited to more than a predefined degree (e.g. more than 50%), in relation to all tested kinases of the particular project.

The selectivity score of the compound(s) at the tested concentrations was calculated for a residual activity < 50%, i.e. an inhibition of > 50%. The selectivity score for a particular compound at a particular concentration was calculated by using the formula (F2):

Selectivity Score = (count of data points < 50%) / (total number of data points) (F2)

Thermodynamic solubility assay

To determine thermodynamic solubility of compounds in PBS, calibration curves for the assayed compounds were prepared using seven concentrations. First, DMSO stock solutions of compounds at 10 mM and 0.5 mM concentration were prepared and then diluted (0.1% TFA in water/acetonitrile (1:1)) to a final concentration of 100 µM; 70 µM; 50 µM; 30 µM; 15 µM; 5 µM and 1 µM (or 10 µM; 7 µM; 5 µM; 3 µM; 1.5 µM; 0.5 µM and 0.1 µM for compound **7f** or 1 µM; 0.7 µM; 0.5 µM; 0.3 µM; 0.15 µM; 0.05 µM and 0.01 µM for compound **7g**).

Quality control (QC) samples were diluted from stock solutions with a 1:1 mixture of 0.1% TFA in water and acetonitrile to final concentrations of 60 µM, 20 µM, and 2 µM (6 µM, 2 µM, and 0.2 µM for **7f** or 0.6 µM, 0.2 µM, and 0.02 µM for **7g**).

Analytical reversed-phase UHPLC analyses were performed on Thermo Scientific Dionex UltiMate 3000 modular system (Thermo Fisher Scientific Inc., MA, USA). Waters Acquity UPLC® HSS C18 SB column (2.1 × 50 mm, 1.8 µm; Waters, Milford, MA, USA) was used at T = 40 °C, with injection volume of 5 µL, flow rate of 0.4 mL/min, and detection at λ = 254 nm. Mobile phase consisted of 0.1% trifluoroacetic acid in ultrapure water (solvent A) and acetonitrile (solvent B). Gradient (for solvent B): 0-12 min, 10-90%; 12-14 min, 90%; 14-15 min, 90-10%. The obtained data was processed in Chromeleon CDS software (Thermo Fisher Scientific Inc., MA, USA)

Analytical method was tested for accuracy, precision and linear rang. Accuracy was determined (n = 3) by comparing the measured concentrations of the quality control samples (20 (or 2 or 0.2) µM and 60 (or 6 or 0.6) µM) to their true values and expressed in (%). Values were within 80-120%. Method repeatability was determined by calculating the RSD from the separately prepared and analysed quality control samples (n = 3). RSD values were ≤ 2 %. Linearity of the method (1 (or 0.1 or 0.01) µM to 100 (or 10 or 1) µM) was evaluated from the 7-point calibration curves (100 µM; 70 µM; 50 µM; 30 µM; 15 µM; 5 µM; 1 µM; or 10-times lower or 100-times lower) determining R^2, which was ≥ 0.996.

Solubility was determined in two independent experiments and results were calculated in Excel (Microsoft, USA). The individual results are given in Table S25.

Table S25. Thermodynamic solubility data.

	Thermodynamic solubility [µM]			Recovery [%]
**Compound ID**	Experiment 1	Experiment 2	Average Result	
**7a**	100.02	96.64	98.33	104.5
**7b**	59.58	76.63	68.10	100.1
**7c**	8.67	11.60	10.14	99.6
**7d**	5.60	6.07	5.84	110.7
**7e**	71.87	88.82	80.35	114.5
**7f**	0.95	0.89	0.92	97.4
**7g**	0.33	0.19	0.26	90.0
**7h**	3.83	3.80	3.82	103.3
**7i**	10.65	no data	10.65	119.7
**7n**	10.99	11.36	11.18	104.4
**7o**	10.60	11.37	10.98	90.5
**7p**	279.08	88.26	183.67	103.8
**7r**	26.74	27.27	27.00	87.7
**16a**	133.66	141.49	137.58	105.1
**16b**	140.21	122.62	131.42	101.6

Thermodynamic solubility of compounds **E** and **7a** (second determination) was determined by the following methods:

- Solid compound **E** was weighed in HPLC vial (1.45 mg). 0.5 mL of 67 mM phosphate buffer (pH 7.4, isotonic, Sorensens) was added. The vial was sealed and incubated for 48h in a rotational shaker (400 rpm) at 23 °C. After the incubation an aliquot of 200 µL was transferred to a conical glass insert and centrifuged for 30 min at 10 000 × g at 23 °C or 37 °C. 1 µL of the supernatant was transferred to 96-well plate in triplicate wells, diluted 100× with acetonitrile/H_2O (60/40) containing 50 nM warfarin as internal standard and analyzed by LC-MS/MS in ESI+ MRM (as described in the article's chapter 6). Quantitation was performed comparing to a separately constructed standard series (10 nM to 1000 nM). Solubility was determined to be 6.6 ± 0.9 µM (mean ± SD) in two independent experiments.
- Solid **7a** was weighed in HPLC vial (2.91 mg) followed by the addition of 0.4 mL of 100 mM phosphate buffer (pH 7.4). The vial was sealed and incubated for 24h in a rotational shaker (900 rpm) at 37 °C. After the incubation an aliquot of 200 µL was transferred to a conical glass insert and centrifuged for 30 min at 10 000 × g at 37 °C. The supernatant was

transferred to a new HPLC vial appropriately diluted (100×, 1000×, 5000×) with acetonitrile/H_2O (50/50) containing 50 nM warfarin as internal standard and analyzed by LC-MS/MS in ESI+ MRM (as described in the article's chapter 6). Quantitation was performed comparing to a separately constructed standard curve between 3 nM to 20000 nM. Solubility was determined to be 167.9 ± 67.0 µM (mean ± SD) in three independent experiments.

X-ray crystallography

Protein expression and purification. The ATPase subdomain of *Pseudomonas aeruginosa* PAO1 DNA gyrase B, corresponding to residues 1-221 of the full-length wild-type protein (UniProtKB entry Q9I7C2) was cloned into a modified pTTQ18 vector and expressed in T7 Express *E. coli* cells (New England BioLabs) without an affinity tag. The protein was purified using successive Q-sepharose, monoQ and phenylsepharose columns. The protein was concentrated in 50 mM Tris×HCl pH 7.5, 10% (v/v) glycerol, 1 mM EDTA, 1 mM DTT to approximately 10 mg/mL. The resulting protein had a calculated molecular weight of 24.502 Da and is designated PaGyrB24.

Crystallization, X-ray data collection and structure solution. Crystals were grown from the above sample in the presence of 1 mM **7a** using the sitting drop vapor diffusion method. Commercially available (Molecular Dimensions, Quiagen) and proprietary crystallization screens were set up in MRC2 96-well crystallization plates (Swissci) with drops of 0.3 μL precipitant and 0.3 μL protein solution using an Oryx 8 liquid handling robot (Douglas Instruments) and then equilibrated against 50 μL of reservoir solution at a constant temperature of 19˚C. Crystals were mounted in Litholoops (Molecular Dimensions) before flash-cooling by immersion in liquid nitrogen prior to transport to the synchrotron. X-ray data were acquired on beamline I04 at Diamond Light Source (Oxfordshire, UK) using an Eiger2 XE 16M hybrid photon counting detector (Dectris), with crystals maintained at 100 K using a Cryojet cryocooler (Oxford Instruments).

X-ray data were integrated and scaled using DIALS[2] via the XIA2 expert system[3] and merged using AIMLESS[4] (data statistics can be found in *Supporting Information*, Table S26). All subsequent data processing was performed using CCP4 suite programs via the CCP4i2 graphical user interface.[5] The structure was solved via molecular replacement in PHASER,[6] and the resulting model was finalized by successive iterations of model building in COOT,[7] and restrained refinement in REFMAC5[8] until no further improvements could be made.

Starting coordinates and restraints for the ligand were generated using AceDRG[9] before docking into a suitable electron density. The final model was validated using MOLPROBITY[10] and the PDB-validation server (https://validate-rcsb-2.wwpdb.org). Refinement and validation statistics are summarized in Table S26.

Crystals of the PaGyrB24-**7a** complex were obtained with a precipitant of 28% (w/v) PEG 3350, 200 mM calcium acetate, and were cryo-cooled directly from the crystallization drop. Data were

recorded to 1.6 Å resolution in space group $P2_12_12$. The structure was solved by molecular replacement using a non-isomorphic structure of PaGyrB24 (PDB accession code 7PTF) giving a single copy of the protein chain in the asymmetric unit with an estimated solvent content of 40%.

Table S26. X-ray data collection and refinement statistics for *P. aeruginosa* GyrB24-**7a** complex

Data collection	
Beamline	I04 Diamond
Wavelength (Å)	0.9795
Detector	Eiger2 XE 16M
Resolution range (Å)	38.63 – 1.60 (1.63 – 1.60)
Space Group	$P2_12_12$
Cell parameters (Å/°)	a = 42.2, b = 115.7, c = 38.6
Total no. of measured intensities	363437 (17268)
Unique reflections	27590 (1351)
Multiplicity	13.2 (12.8)
Mean $I/\sigma(I)$	13.8 (1.0)
Completeness (%)	100.0 (100.0)
R_{merge}[a]	0.097 (2.649)
R_{meas}[b]	0.101 (2.759)
$CC_{½}$[c]	1.000 (0.655)
Wilson B value (Å^2)	25.9
Refinement	
Resolution range (Å)	38.63-1.60 (1.64-1.60)
Reflections: working/free[d]	26202/1336
R_{work}/ R_{free}[e]	0.181/0.223 (0.402/0.399)
Ramachandran plot: favoured/allowed/disallowed[f] (%)	98.5/1.5/0.0
R.m.s. bond distance deviation (Å)	0.008
R.m.s. bond angle deviation (°)	1.39
RSCC (inhibitor)[g]	0.99
No. protein residues/residue ranges	206/14-219
No. of water/inhibitor/calcium	216/1/2
Mean B factors: protein/water/inhibitor/calcium (Å^2)	32/40/24/44
Accession code	8BN6

Values in parentheses are for the outer resolution shell.

[a] $R_{merge} = \sum_{hkl} \sum_i |I_i(hkl) - \langle I(hkl)\rangle| / \sum_{hkl} \sum_i I_i(hkl)$.

[b] $R_{meas} = \sum_{hkl} [N/(N-1)]^{1/2} \times \sum_i |I_i(hkl) - \langle I(hkl)\rangle| / \sum_{hkl} \sum_i I_i(hkl)$, where $I_i(hkl)$ is the ith observation of reflection *hkl*, $\langle I(hkl)\rangle$ is the weighted average intensity for all observations i of reflection *hkl* and N is the number of observations of reflection *hkl*.

[c] $CC_{½}$ is the correlation coefficient between symmetry equivalent intensities from random halves of the dataset.

[d] The dataset was split into "working" and "free" sets consisting of 95 and 5% of the data respectively. The free set was not used for refinement.
[e] The R-factors R_{work} and R_{free} are calculated as follows: $R = \sum(| F_{obs} - F_{calc} |)/\sum| F_{obs} |$, where F_{obs} and F_{calc} are the observed and calculated structure factor amplitudes, respectively.
[f] As calculated using MolProbity.
[g] Real Space Correlation Coefficient as calculated by the PDB validation server.

In vitro cell micronucleus test

Micronucleus assay was performed on Chinese hamster ovary (CHO) cells, the cells were seeded at a density of 2000 cells per well in a black 96-well plate with clear bottom and incubated in a humidified atmosphere at 37 °C with 5% CO_2. To estimate the micronuclei frequency, the cells scored must have completed one mitosis during the treatment or the post-treatment incubation period. For the test without metabolic activation by rat liver S9 fraction, **7a** was prepared at 10 mM in DMSO 100% and assayed at 6.25, 12.5, 25, 50, and 100 μM, during 24 h. DMSO should not exceed 1% according to TG-487. Mitomycin C (MitC, Sigma Aldrich), a known inducer of micronuclei formation, was the positive control used to demonstrate the sensitivity of the test, and cells untreated are negative control. For the test with metabolic activation by rat liver S9 fraction, **7a** was assayed at 25, 50, and 100 μM. Benzo(a)pyrene (Sigma Aldrich) was the positive control for the system. Regent used for metabolic activation was a 2% of the mix of co-factor-supplemented post-mitochondrial fraction (S9) prepared from the livers of rats treated with enzyme-inducing agents Aroclor 1254, Glucose-6-phosphate, NADP and buffer salts (Xenometrix). After treatment, Cytochalasin B (cytoB) was used as cytokinesis-blocker of cultures during 28 h (without S9 activation) or 42 h (with S9 activation). Cells were then fixed with 3.7% formaldehyde and 1% Triton X-100 and nuclei were stained with bisbenzimide (Hoechst dye no. 33258) for 30 min at room temperature. Imaging acquisition was performed by using Operetta CLS High-Content Analysis System (Perkin Elmer). Analysis was performed using Harmony software of Perkin Elmer and the in-house App NucleusFinder, based on an open source processing image program, ImageJ. The cytokinesis-block proliferation index (CBPI), which indicates the average number of cell cycles per cell during the period of exposure to cytoB, is used to estimate the cytostatic activity of a treatment by comparing values in the treated and control cultures. Cytostasis percentage should not to exceed 60% because higher levels may induce micronuclei as a secondary effect of cytotoxicity and is calculated with formula F3:

$$\% \text{ Cytostasis} = 100 - 100 \times [(CBPI_T - 1) / (CBPI_C - 1)] \qquad (F3),$$

where T is test chemical treatment culture, C is vehicle control culture and CBPI is defined with the following equation (F4):

CBPI = [(No. mononucleate cells) + (2 × No. binucleate cells) + (3 × No. multinucleate cells)] / (total number of cells) (F4)

7a was assayed in six replicates, counting between 1000-2000 binucleate cells, when there was no cytotoxicity or cytostasis, in six independent wells with at least 35 image fields captured per well with magnification of 20×.

^{1}H and ^{13}C NMR spectra for representative compounds

^{1}H and ^{13}C NMR spectrum of 2-(3,4-dichloro-5-methyl-1*H*-pyrrole-2-carboxamido)-4-morpholinobenzo[*d*]thiazole-6-carboxylic acid (**7a**).

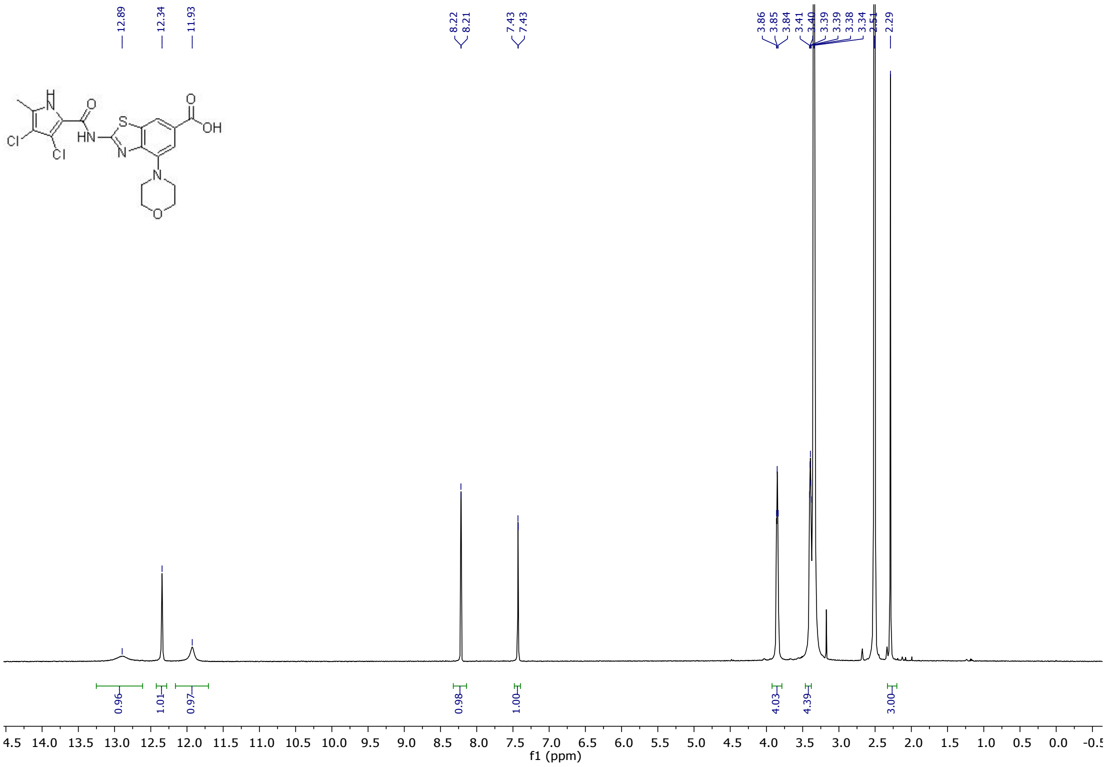

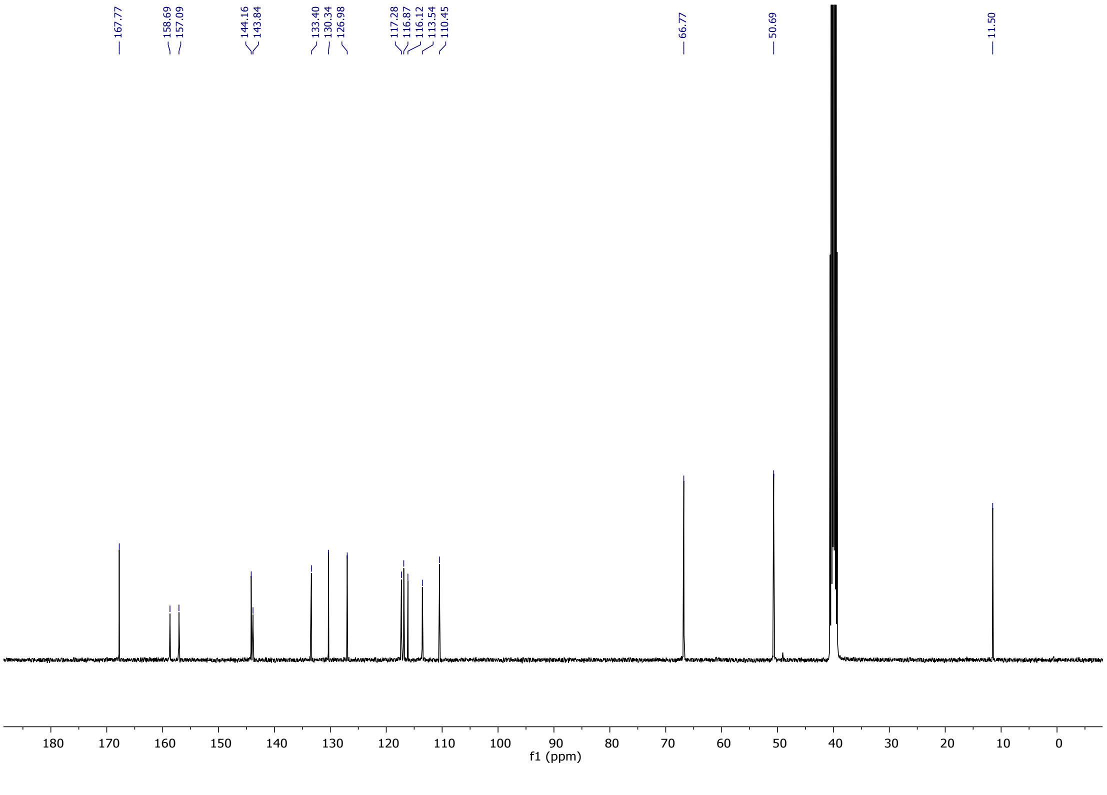

^{1}H and ^{13}C NMR spectrum of 2-(3,4-dichloro-5-methyl-1*H*-pyrrole-2-carboxamido)-4-(2-methylmorpholino)benzo[*d*]thiazole-6-carboxylic acid (**7e**).

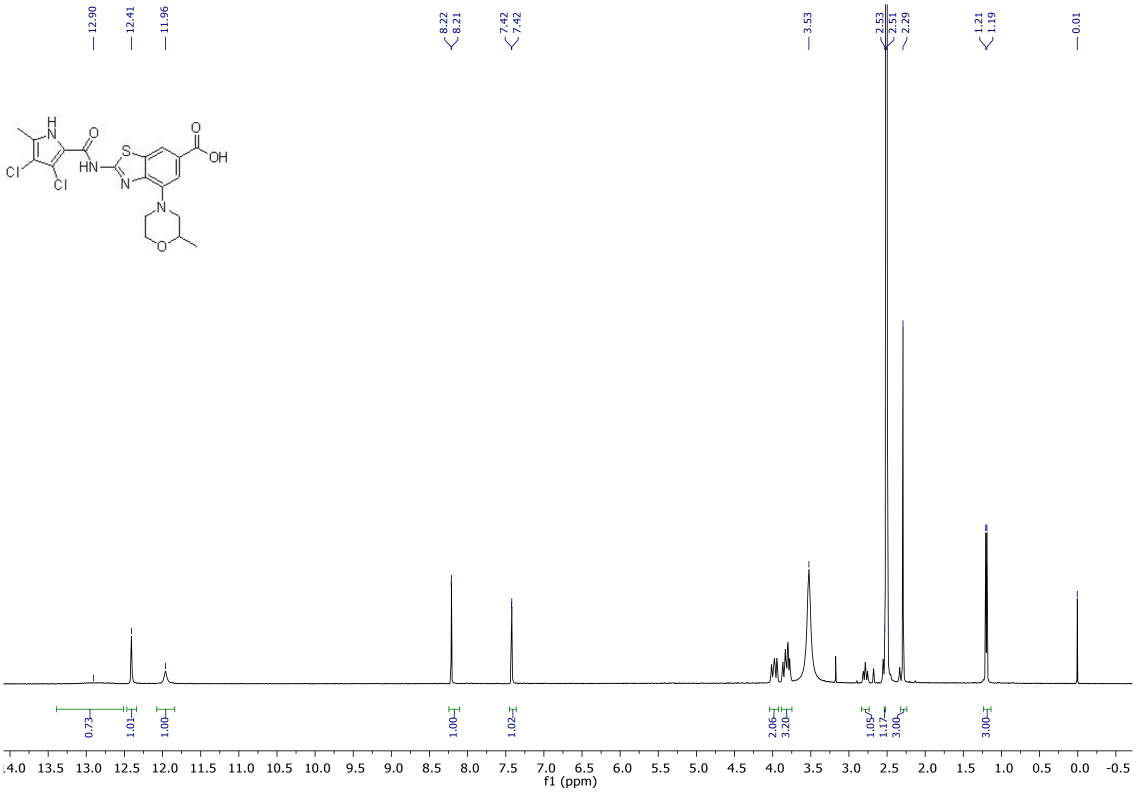

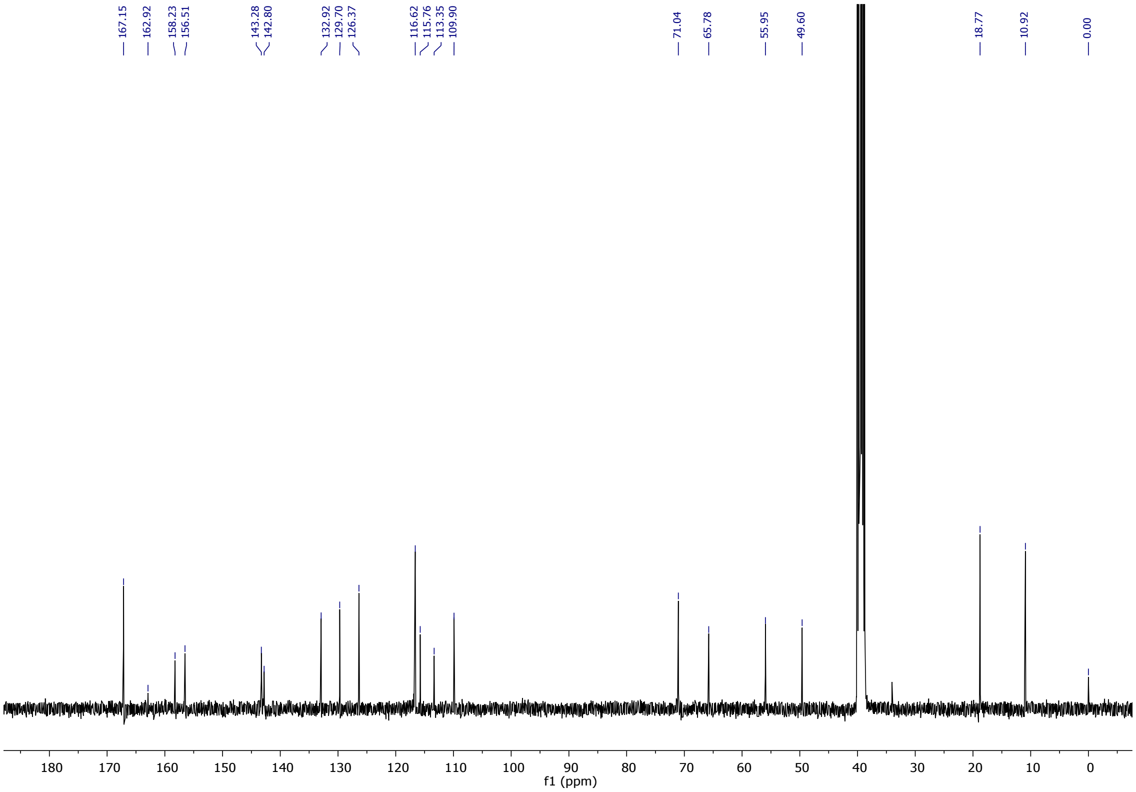

^{1}H and ^{13}C NMR spectrum of 2-(3,4-Dichloro-5-methyl-1*H*-pyrrole-2-carboxamido)-4-(2,6-dimethylmorpholino)benzo[*d*]thiazole-6-carboxylic acid (**7f**).

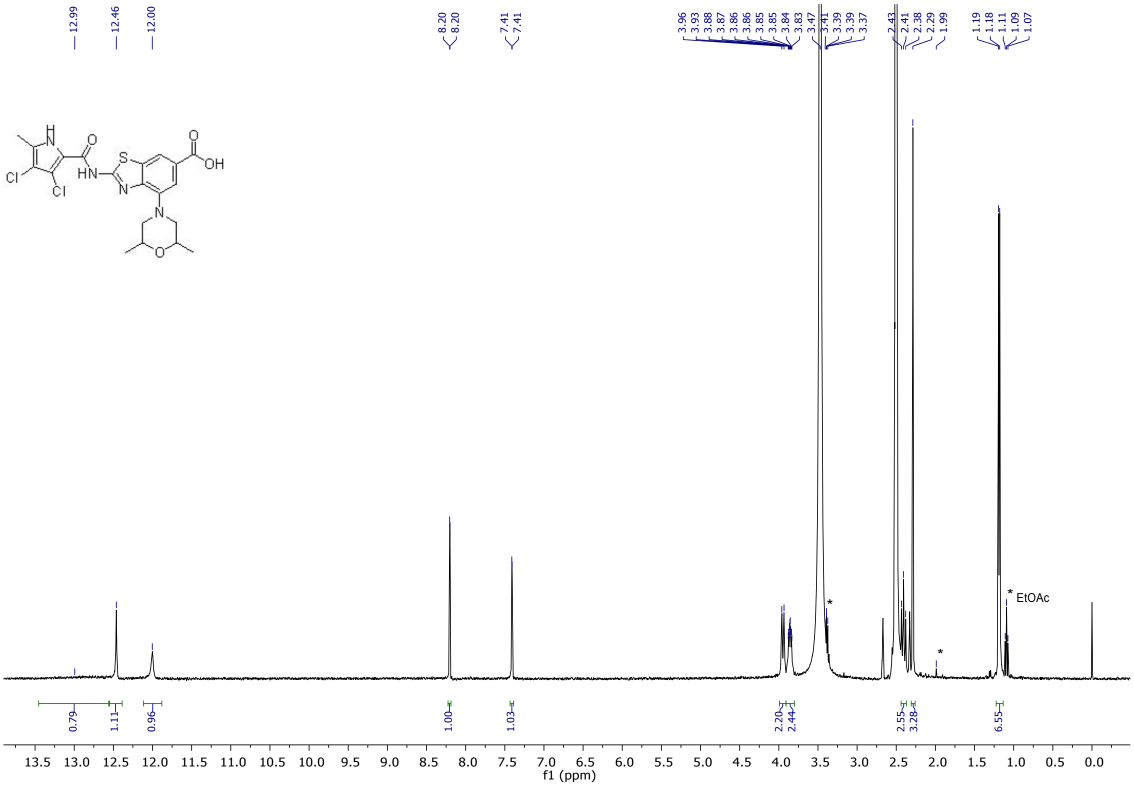

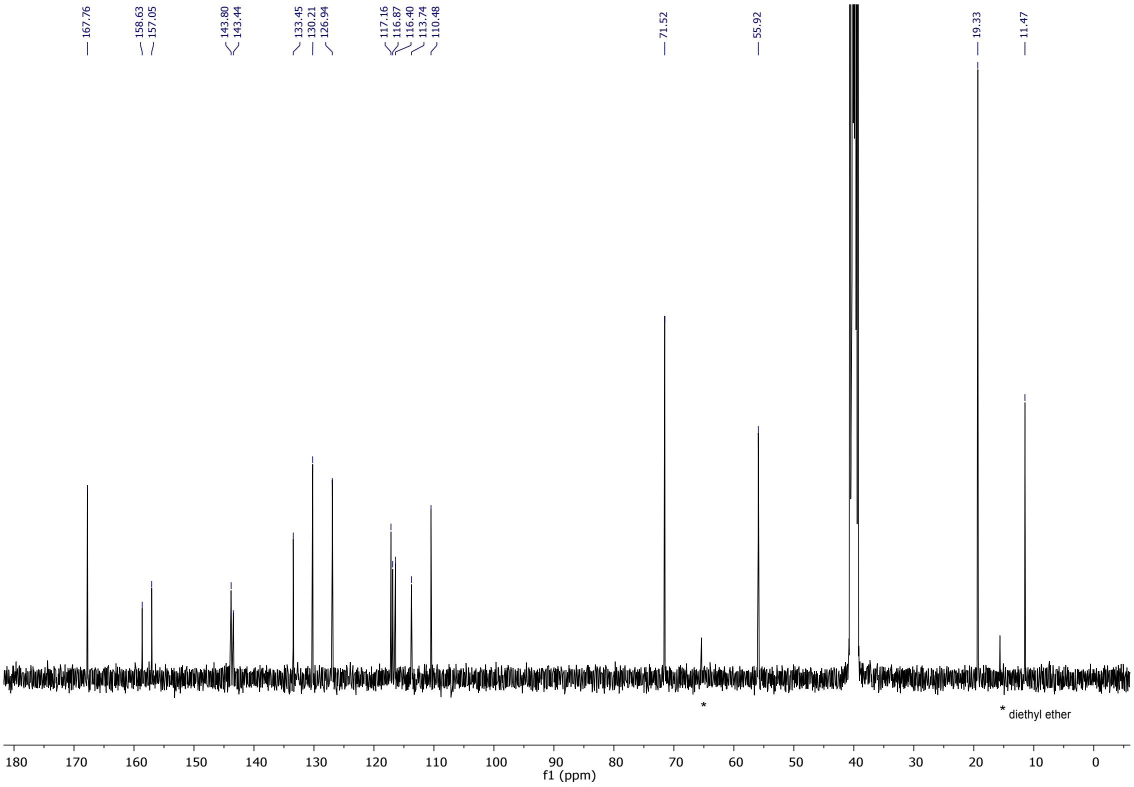

^{1}H and ^{13}C NMR spectrum of 4-((1*R*,5*S*)-8-oxa-3-azabicyclo[3.2.1]octan-3-yl)-2-(3,4-dichloro-5-methyl-1*H*-pyrrole-2-carboxamido)benzo[*d*]thiazole-6-carboxylic acid(**7g**).

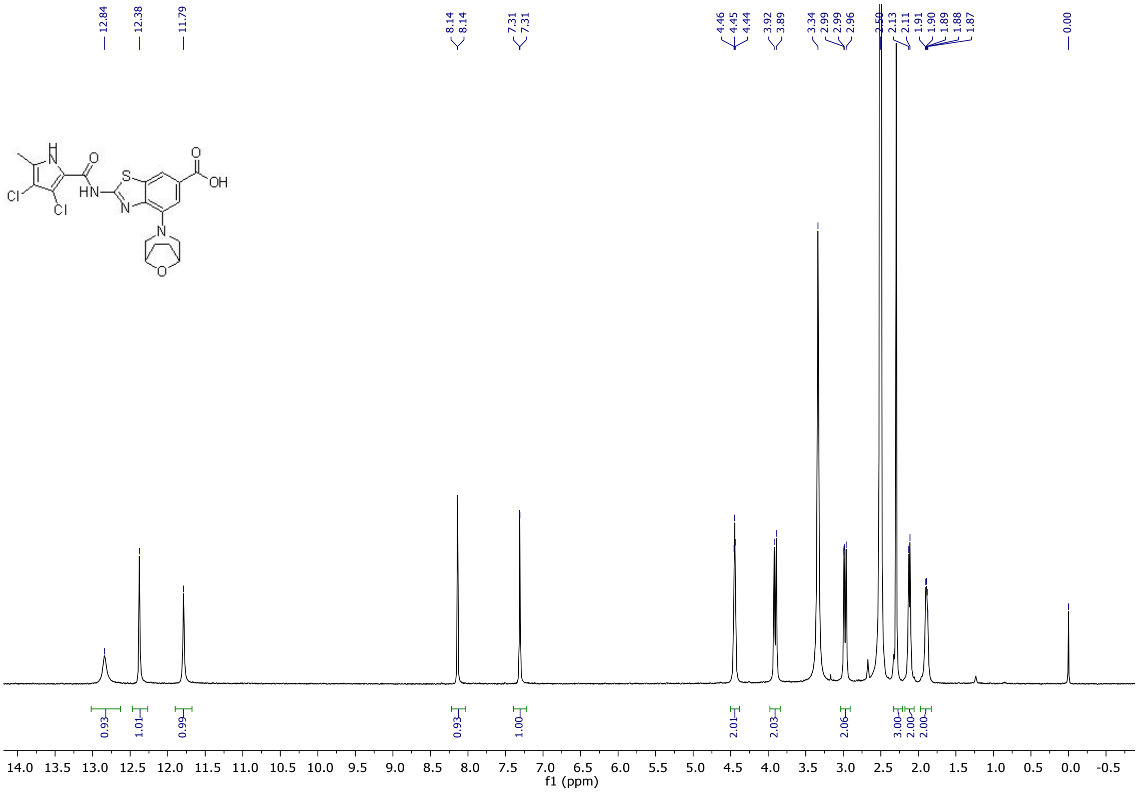

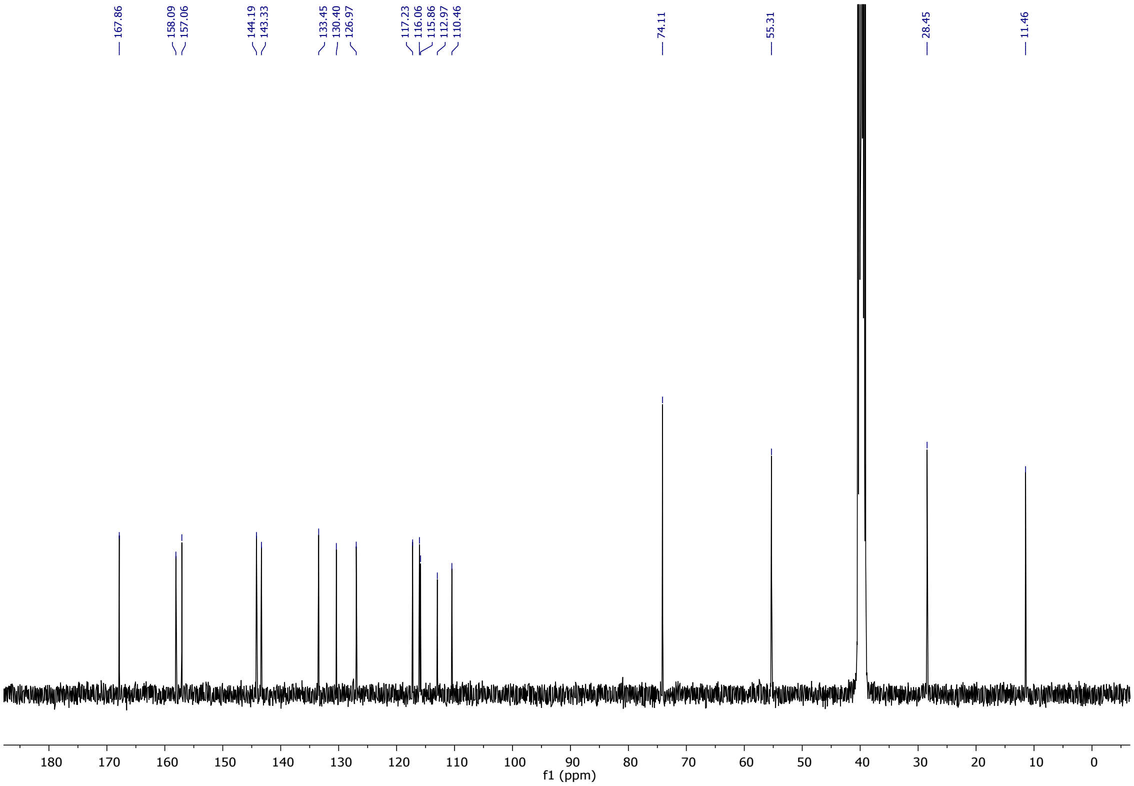

^{1}H and ^{13}C NMR spectrum of 4-((1*R*,5*S*)-3-oxa-8-azabicyclo[3.2.1]octan-8-yl)-2-(3,4-dichloro-5-methyl-1*H*-pyrrole-2-carboxamido)benzo[*d*]thiazole-6-carboxylic acid (**7h**).

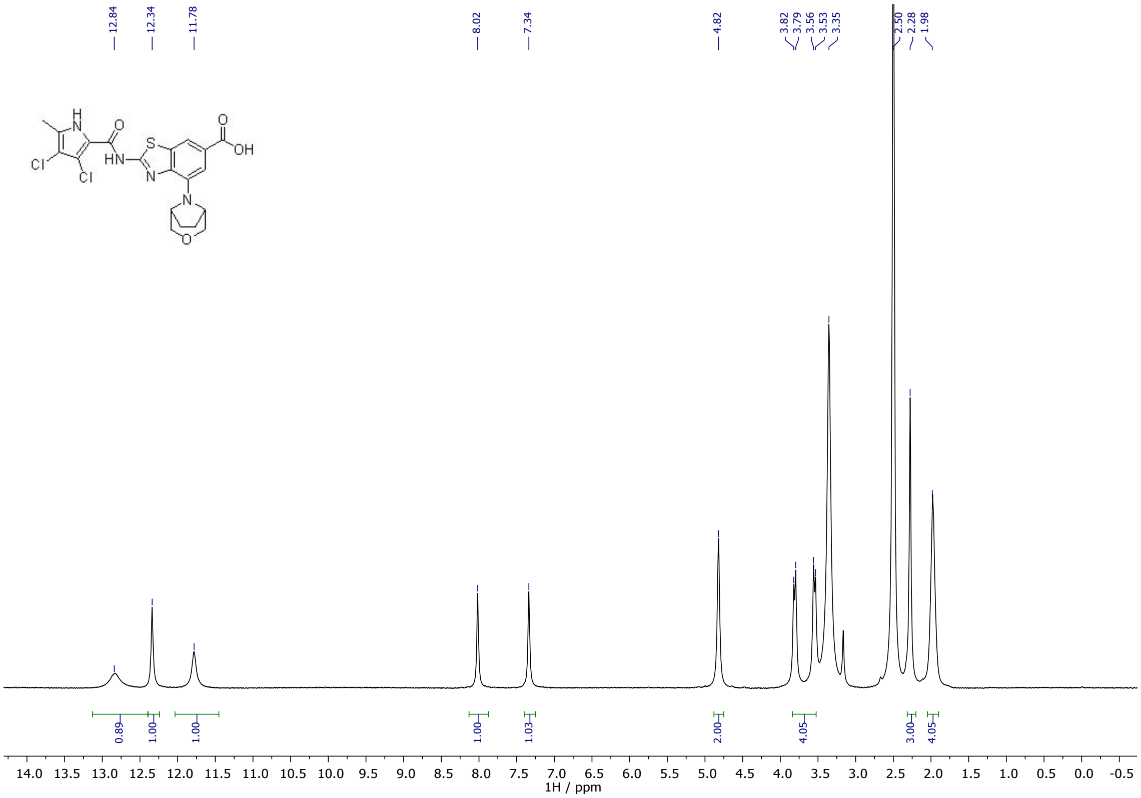

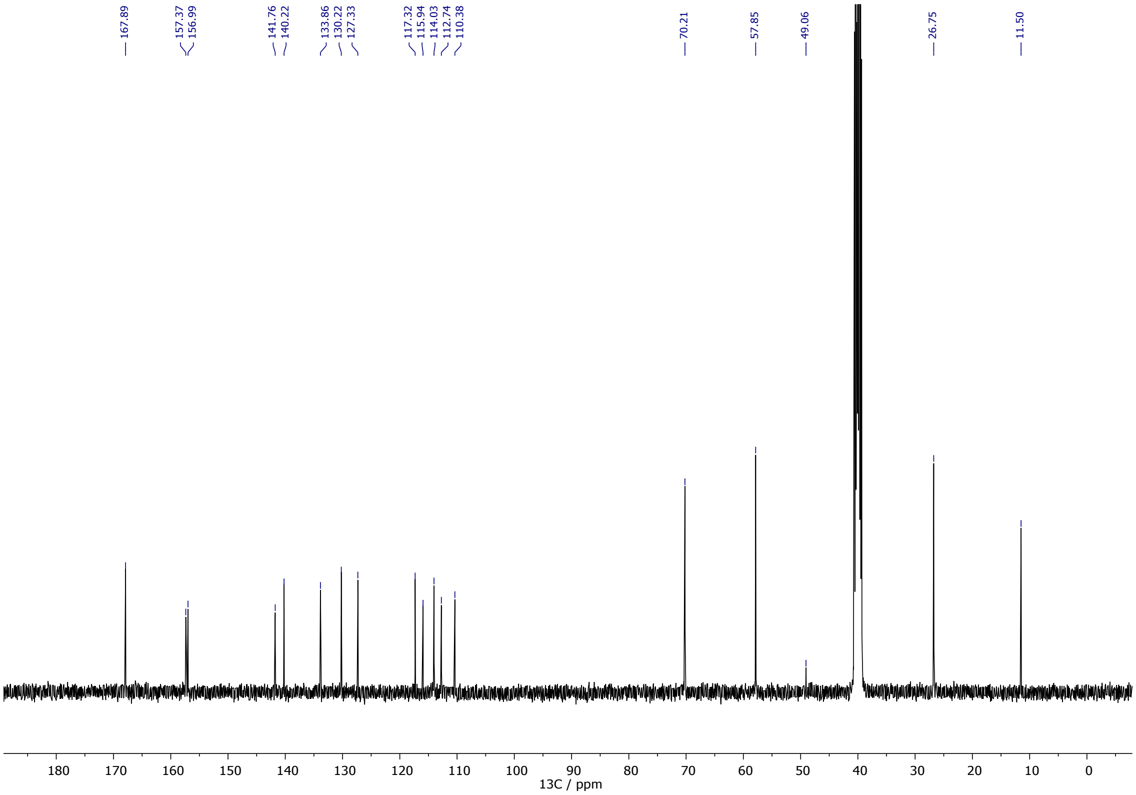

^{1}H and ^{13}C NMR spectrum of 2-(3,4-dichloro-5-methyl-1*H*-pyrrole-2-carboxamido)-4-(2-(trifluoromethyl)morpholino)benzo[*d*]thiazole-6-carboxylic acid (**7i**).

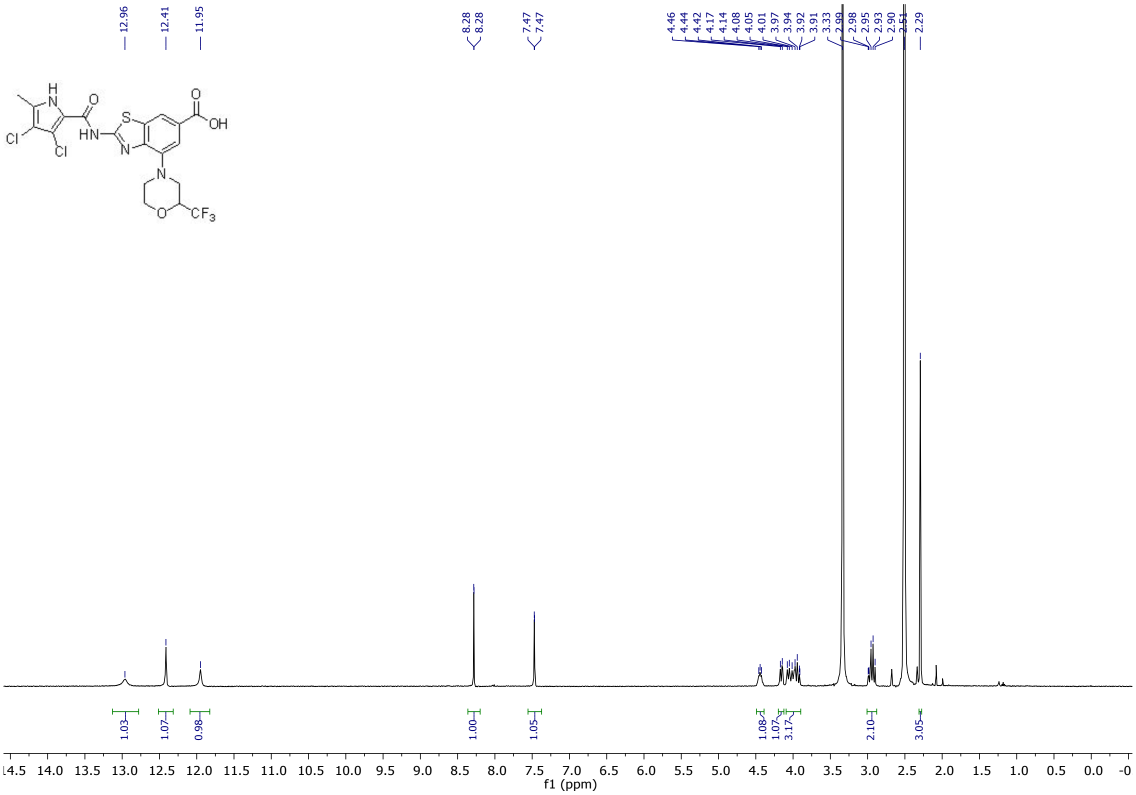

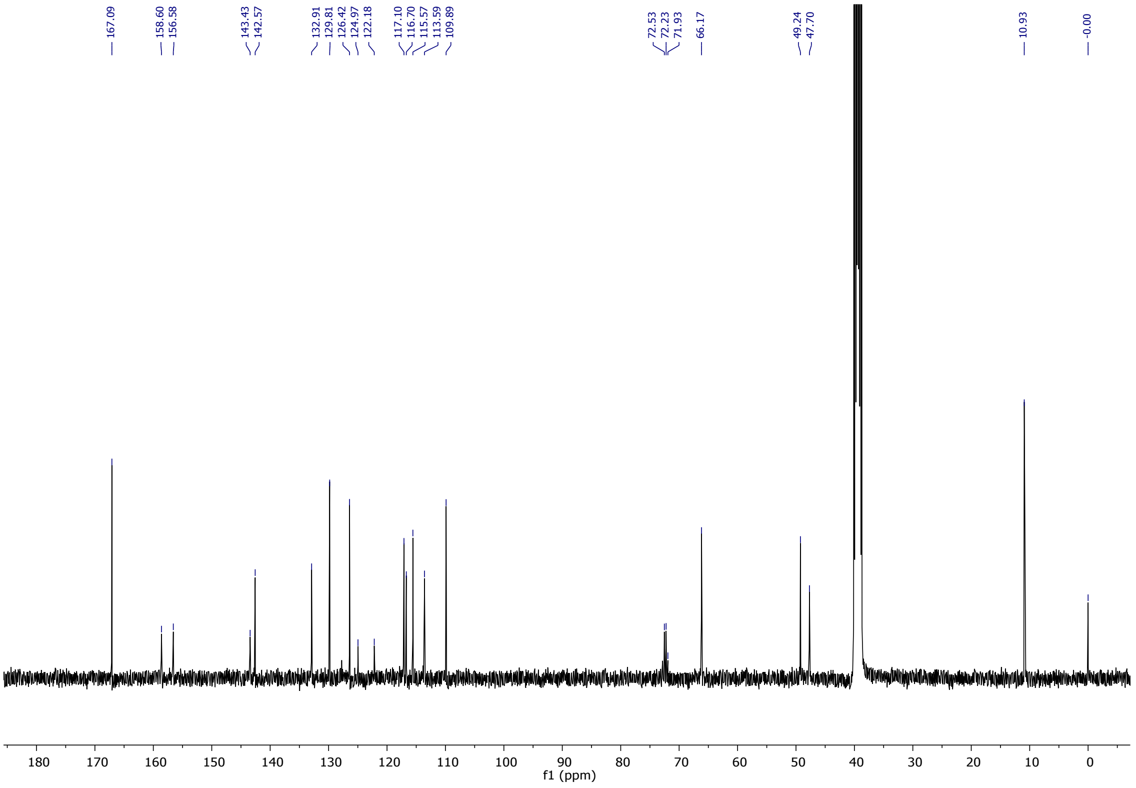

^{1}H and ^{13}C NMR spectrum of 2-(3,4-dichloro-5-methyl-1*H*-pyrrole-2-carboxamido)-4-(2-oxooxazolidin-3-yl)benzo[*d*]thiazole-6-carboxylic acid (**7m**).

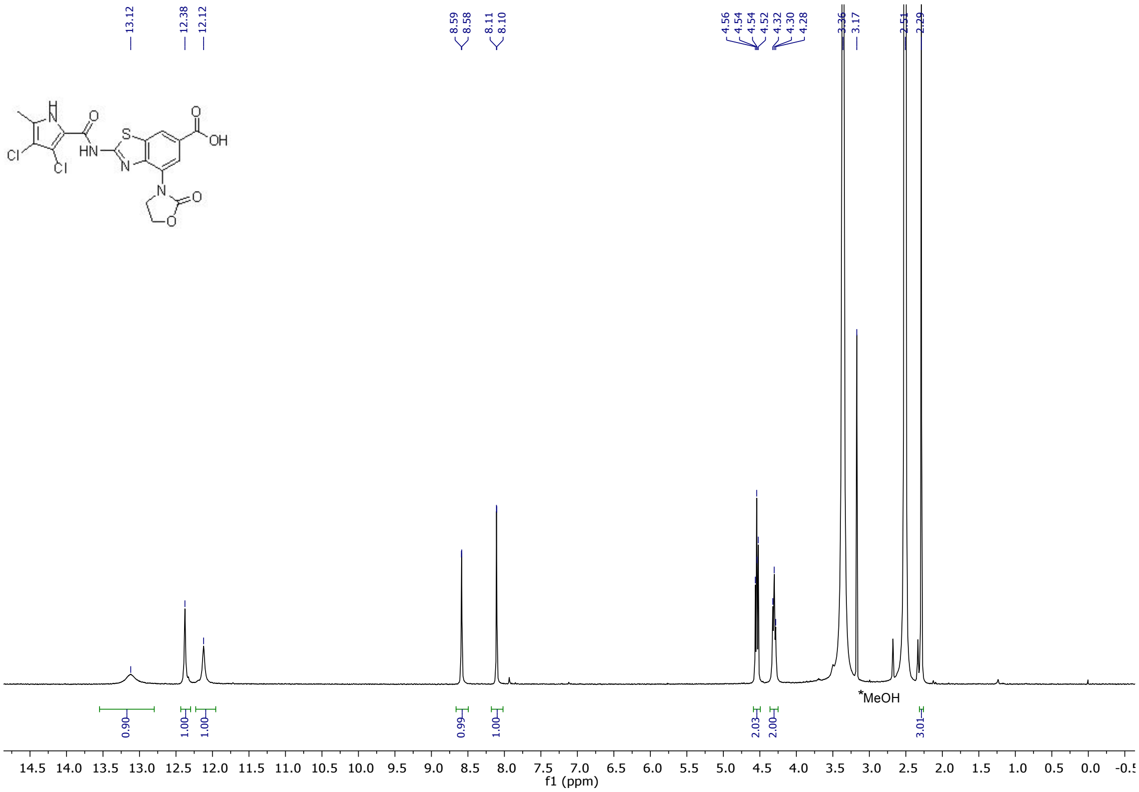

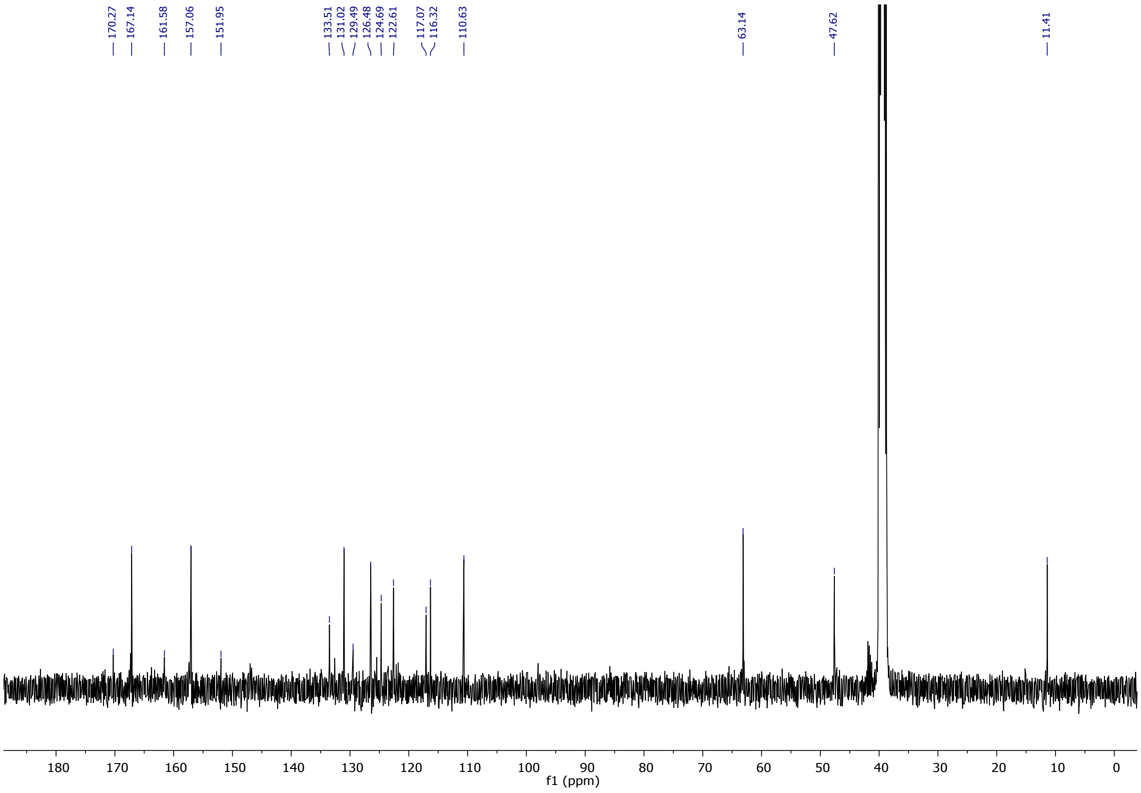

^{1}H and ^{13}C NMR spectrum of 2-(3,4-dichloro-5-methyl-1*H*-pyrrole-2-carboxamido)-4-(1,4-dioxa-8-azaspiro[4.5]decan-8-yl)benzo[*d*]thiazole-6-carboxylic acid (**7r**).

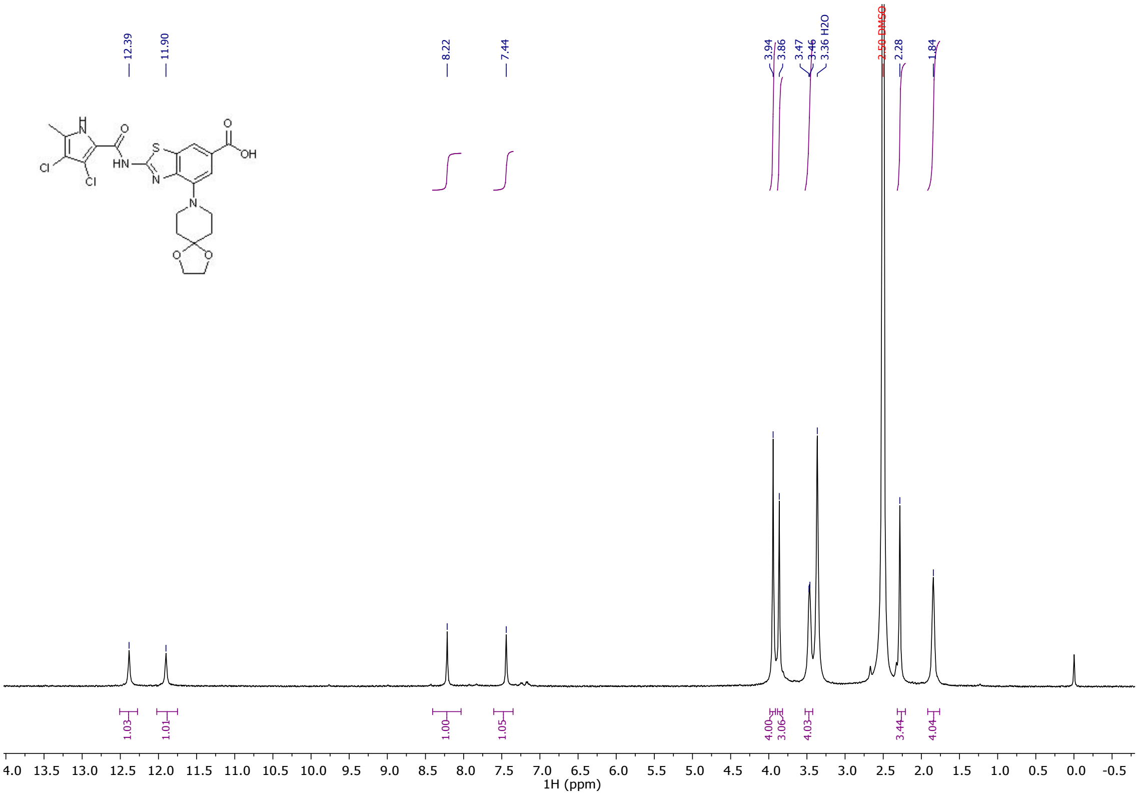

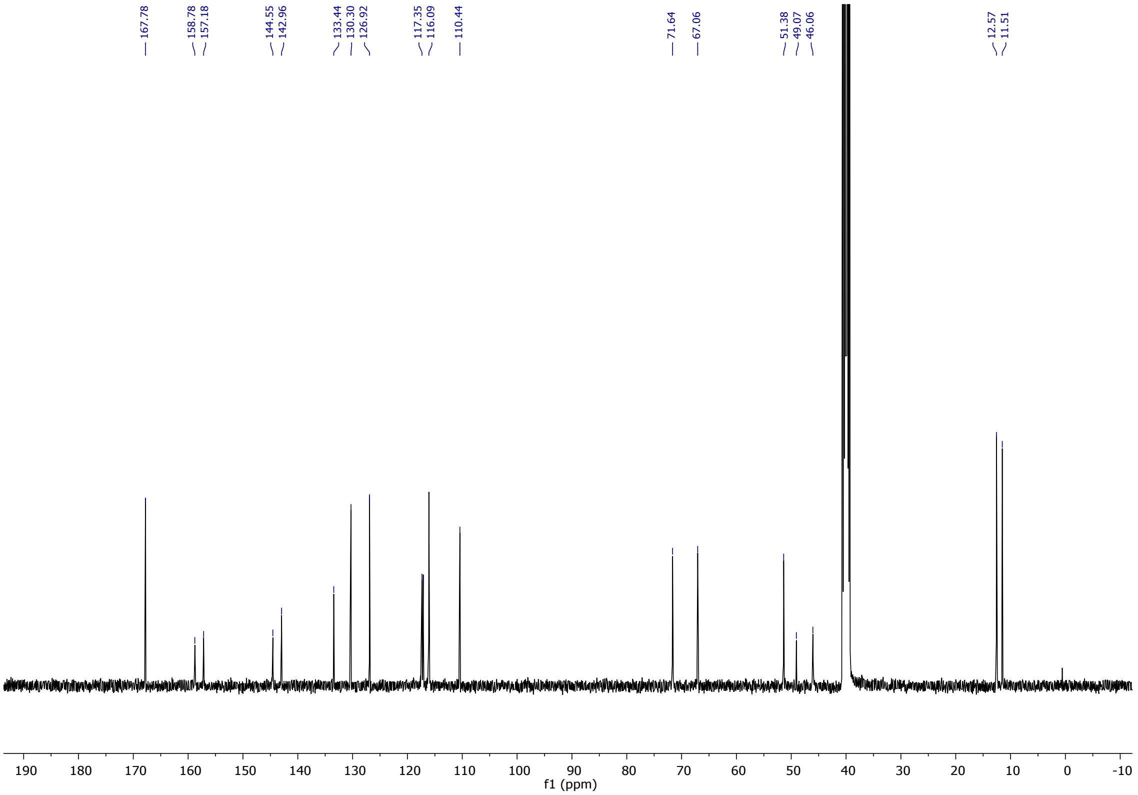

^{1}H and ^{13}C NMR spectrum of 2-(3,4-dichloro-5-methyl-1*H*-pyrrole-2-carboxamido)-4-((3-methoxypropyl)amino)benzo[*d*]thiazole-6-carboxylic acid (**8c**).

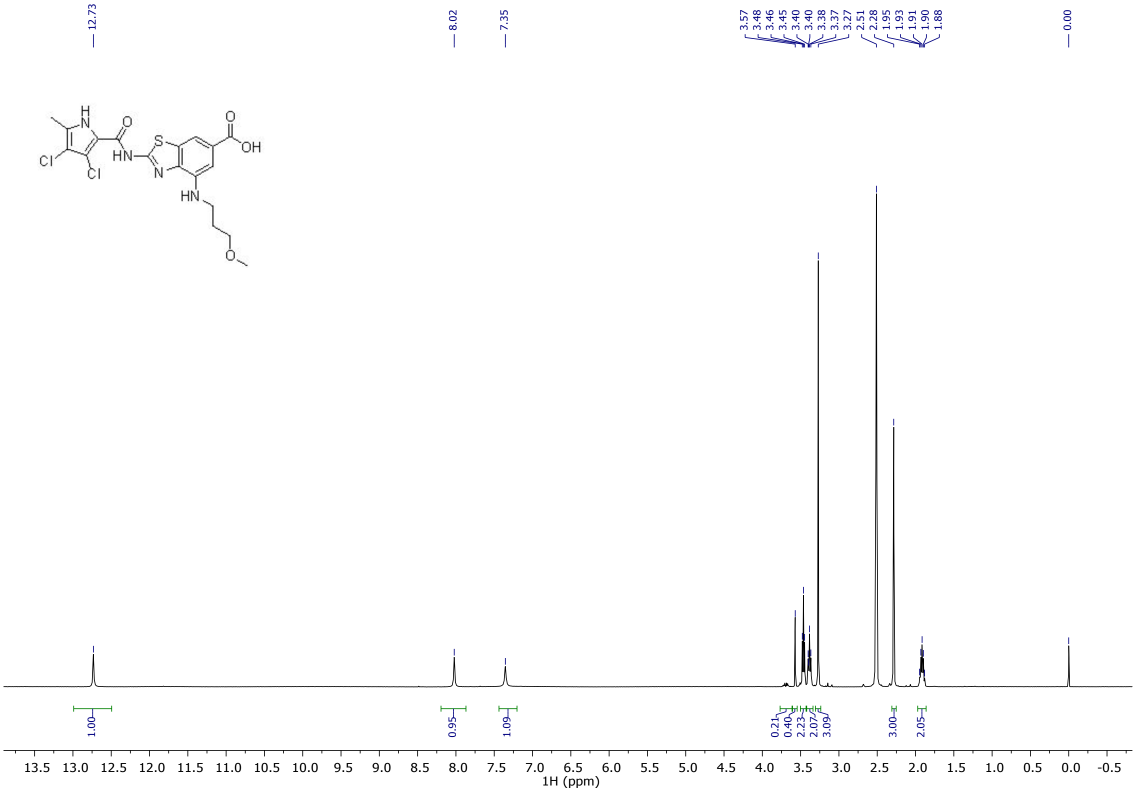

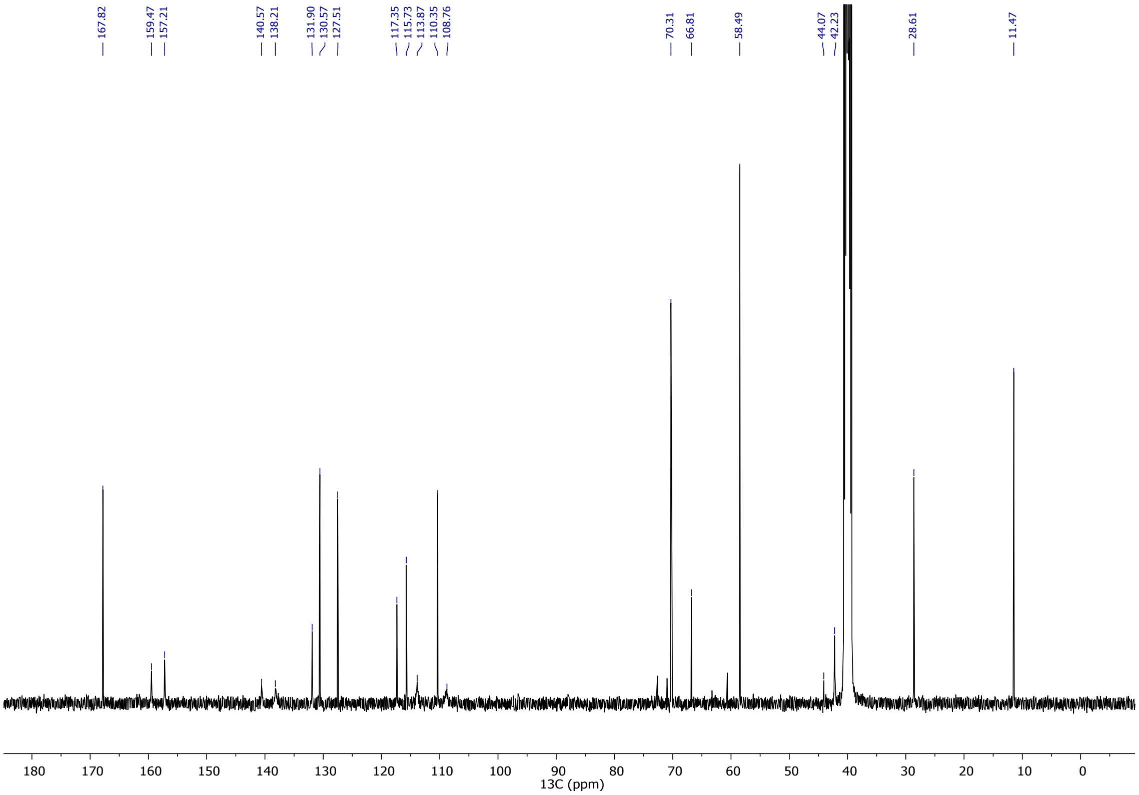

^{1}H and ^{13}C NMR spectrum of methyl 2-(4-chloro-5-methyl-1*H*-pyrrole-2-carboxamido)-4-morpholinobenzo[*d*]thiazole-6-carboxylate (**16a**).

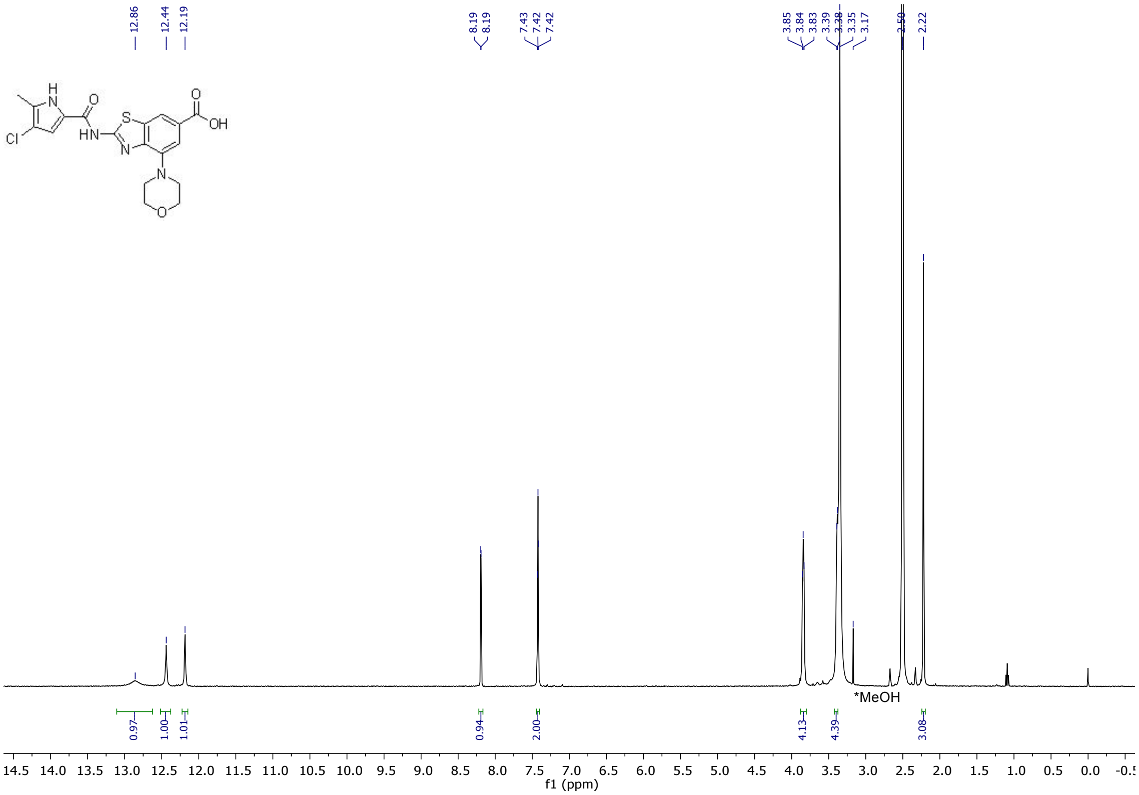

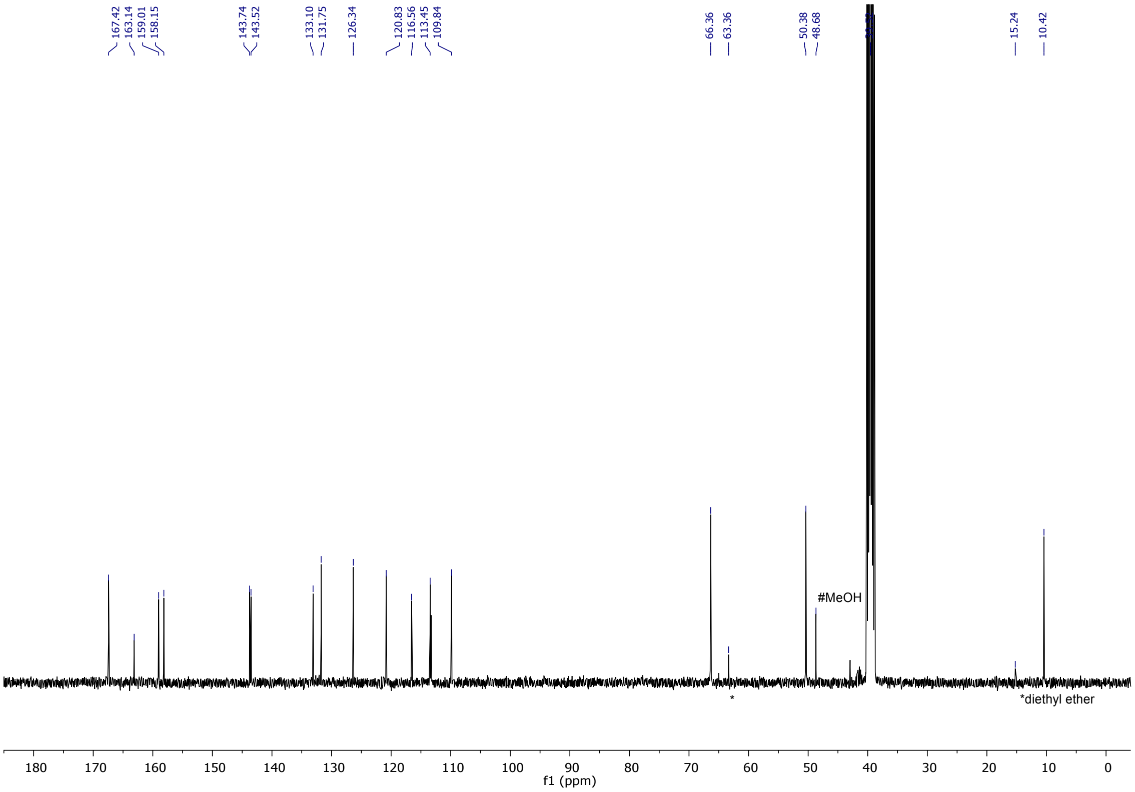

^{1}H and ^{13}C NMR spectrum of 2-(4-fluoro-5-methyl-1*H*-pyrrole-2-carboxamido)-4-morpholinobenzo[*d*]thiazole-6-carboxylic acid (**16b**).

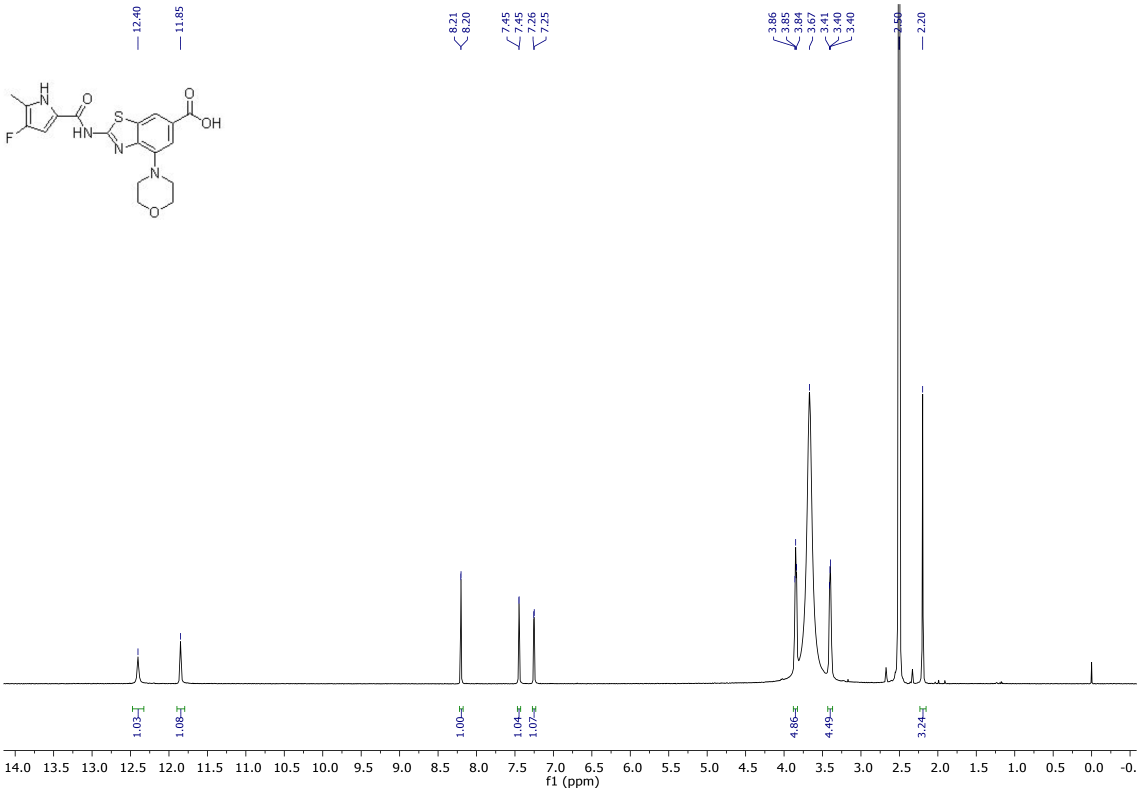

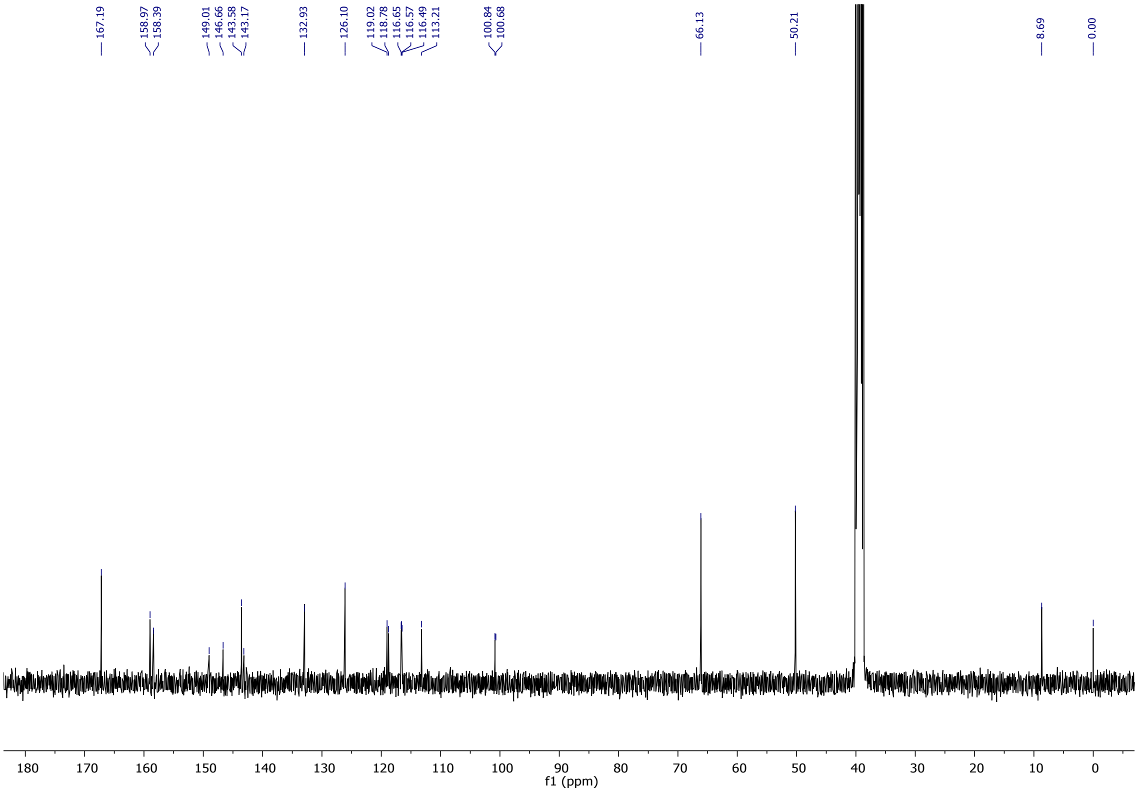

^{1}H and ^{13}C NMR spectrum of 2-(4-cyano-5-methyl-1*H*-pyrrole-2-carboxamido)-4-morpholinobenzo[*d*]thiazole-6-carboxylic acid (**16c**).

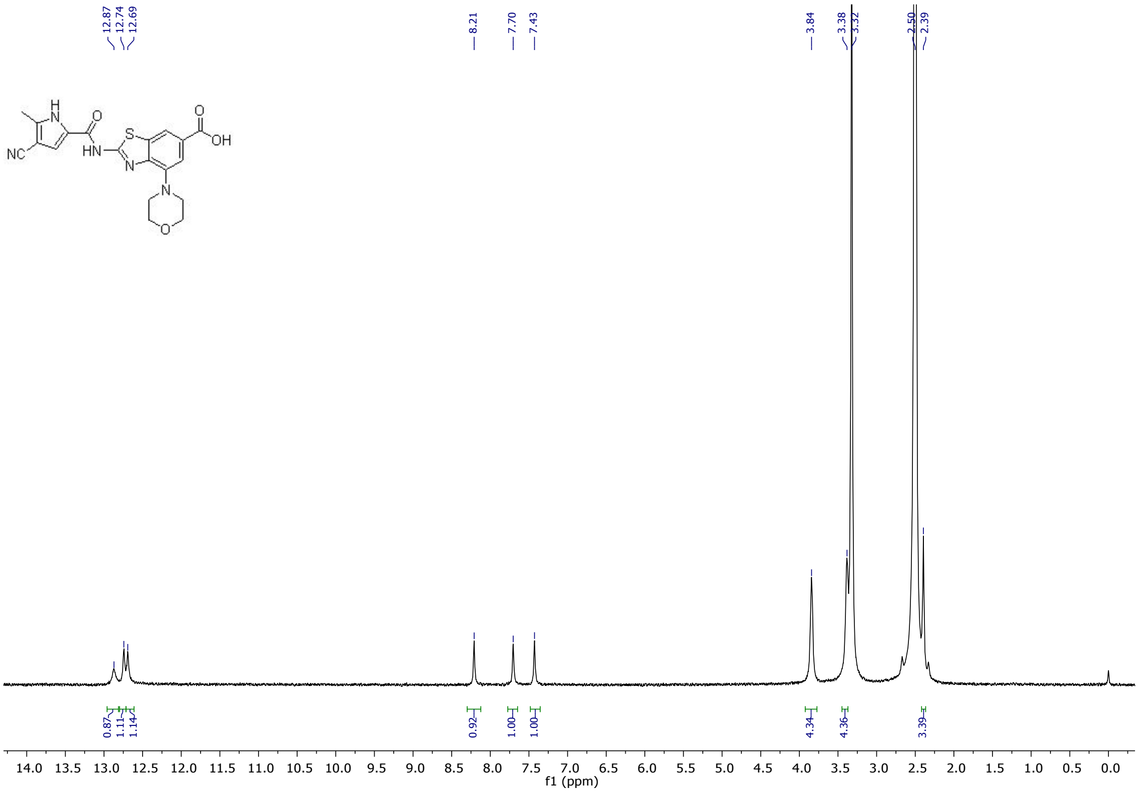

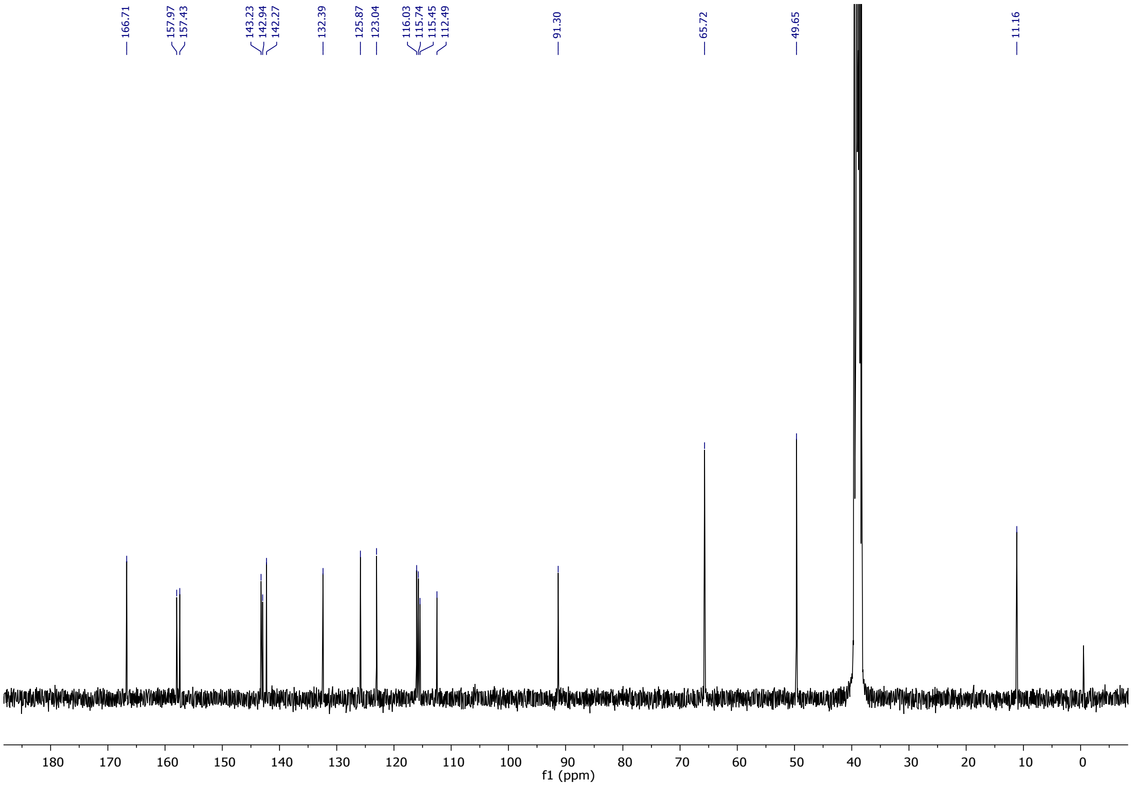

HRMS data for lead compound 7a

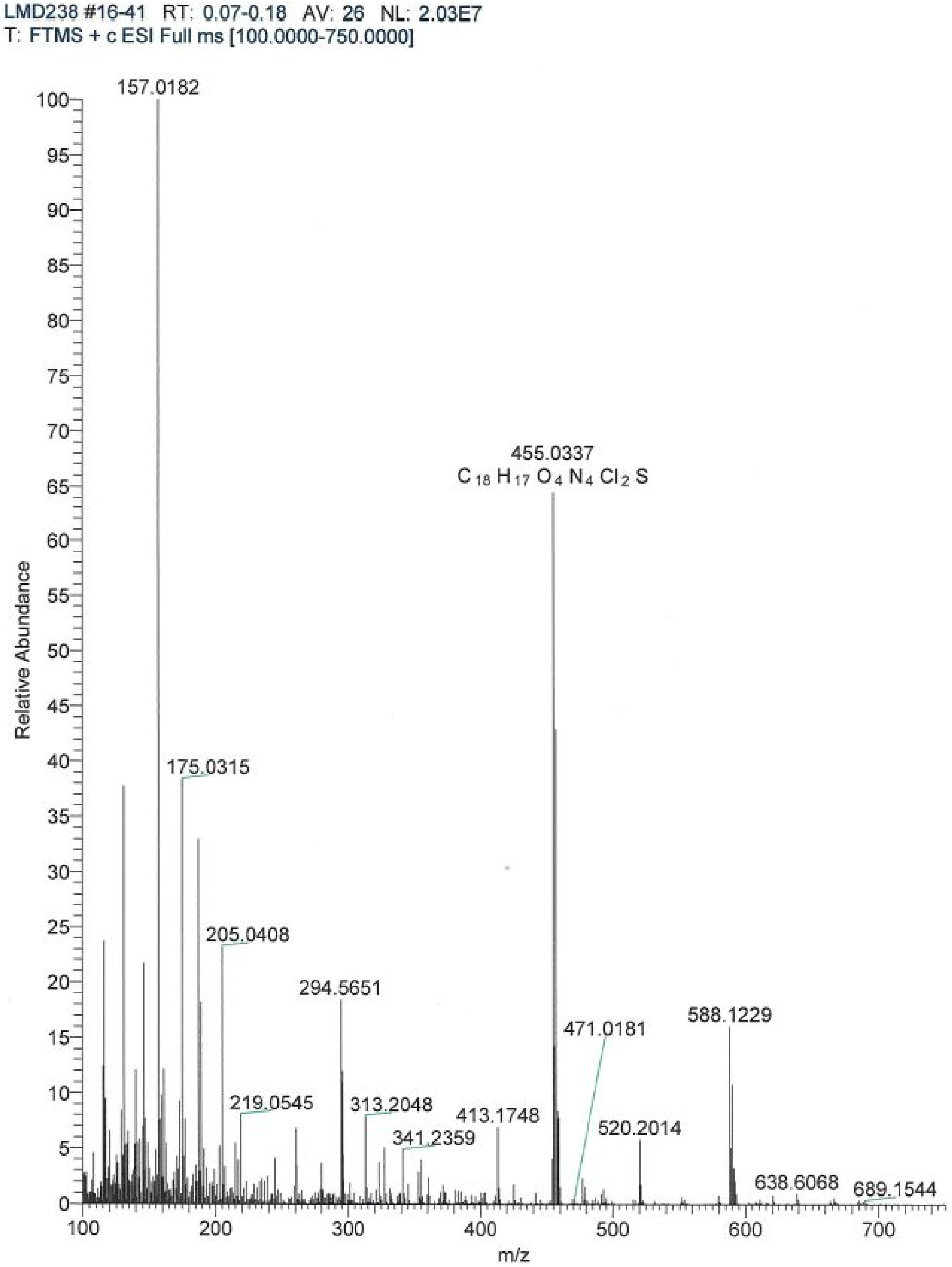

Elemental composition search on mass 455.0338

m/z= 450.0338-460.0338

m/z	Theo. Mass	Delta (ppm)	RDB equiv.	Composition
455.03375	455.03421	-1.01	11.5	$C_{18} H_{17} O_4 N_4 Cl_2 S$

HPLC traces for lead compound 7a

- Thermo Scientific Dionex UltiMate 3000 modular system (Thermo Fisher Scientific Inc., MA, USA).
- column: Waters Acquity UPLC® HSS C18 SB (2.1 × 50 mm, 1.8 µm; Waters, Milford, MA, USA),
- column temperature: T = 40 °C,
- injection volume: 5 µL,
- flow rate: 0.4 mL/min,
- detection UV 254 nm,
- mobile phase: 0.1% trifluoroacetic acid in ultrapure water (solvent A) and acetonitrile (solvent B). Gradient (for solvent B): 0-8 min, 10-90%; 8-10 min, 90%; 10-11 min, 90-10%.

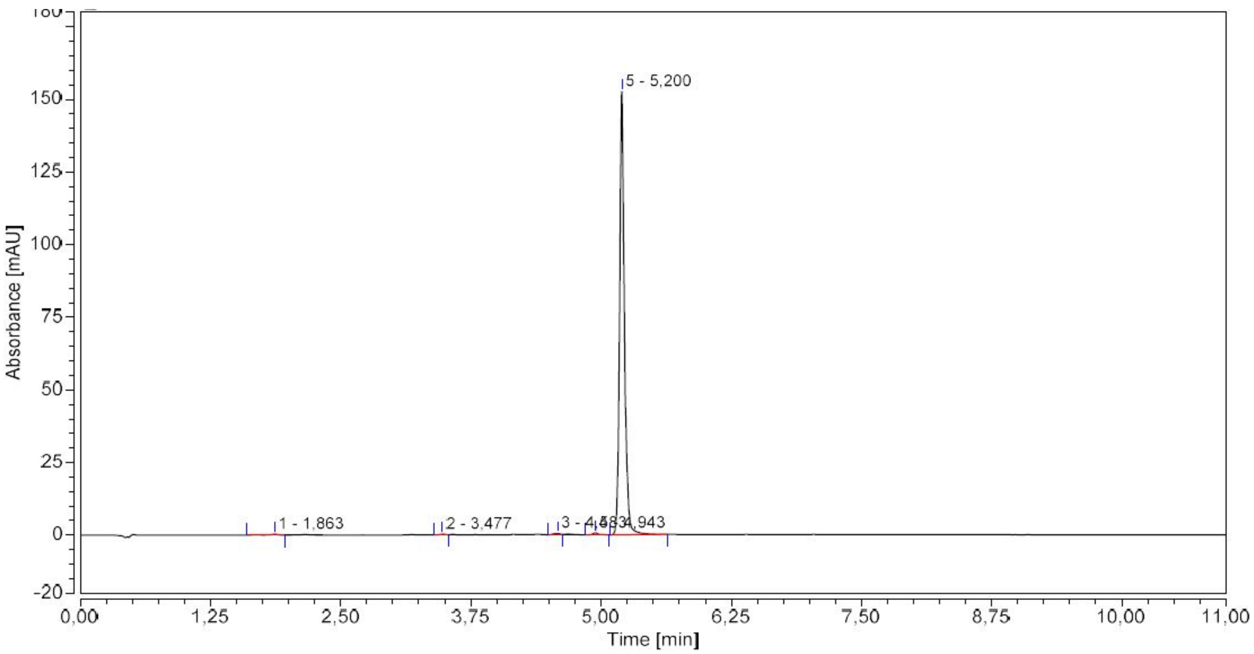

Integration Results

No.	Peak Name	Retention Time min	Area mAU*min	Height mAU	Relative Area %	Relative Height %	Amount n.a.
1		1,863	0,032	0,346	0,39	0,22	n.a.
2		3,477	0,018	0,291	0,21	0,19	n.a.
3		4,583	0,034	0,471	0,42	0,30	n.a.
4		4,943	0,042	0,738	0,51	0,48	n.a.
5		5,200	8,106	152,583	98,46	98,81	n.a.
**Total:**			**8,233**	**154,429**	**100,00**	**100,00**	

References

(1) Karaman, M. W.; Herrgard, S.; Treiber, D. K.; Gallant, P.; Atteridge, C. E.; Campbell, B. T.; Chan, K. W.; Ciceri, P.; Davis, M. I.; Edeen, P. T.; Faraoni, R.; Floyd, M.; Hunt, J. P.; Lockhart, D. J.; Milanov, Z. V.; Morrison, M. J.; Pallares, G.; Patel, H. K.; Pritchard, S.; Wodicka, L. M.; Zarrinkar, P. P. A Quantitative Analysis of Kinase Inhibitor Selectivity. *Nat. Biotechnol.* **2008**, *26* (1), 127–132. https://doi.org/10.1038/nbt1358.

(2) Kabsch, W. *XDS*. *Acta Crystallogr. D Biol. Crystallogr.* **2010**, *66* (2), 125–132. https://doi.org/10.1107/S0907444909047337.

(3) Winter, G. *Xia2* : An Expert System for Macromolecular Crystallography Data Reduction. *J Appl. Crystallogr.* **2010**, *43* (1), 186–190. https://doi.org/10.1107/S0021889809045701.

(4) Evans, P. R.; Murshudov, G. N. How Good Are My Data and What Is the Resolution? *Acta Crystallogr. D Biol. Crystallogr.* **2013**, *69* (7), 1204–1214. https://doi.org/10.1107/S0907444913000061.

(5) Potterton, L.; Agirre, J.; Ballard, C.; Cowtan, K.; Dodson, E.; Evans, P. R.; Jenkins, H. T.; Keegan, R.; Krissinel, E.; Stevenson, K.; Lebedev, A.; McNicholas, S. J.; Nicholls, R. A.; Noble, M.; Pannu, N. S.; Roth, C.; Sheldrick, G.; Skubak, P.; Turkenburg, J.; Uski, V.; von Delft, F.; Waterman, D.; Wilson, K.; Winn, M.; Wojdyr, M. *CCP* 4 *i* 2: The New Graphical User Interface to the *CCP* 4 Program Suite. *Acta Crystallogr. D Struct. Biol.* **2018**, *74* (2), 68–84. https://doi.org/10.1107/S2059798317016035.

(6) McCoy, A. J.; Grosse-Kunstleve, R. W.; Adams, P. D.; Winn, M. D.; Storoni, L. C.; Read, R. J. *Phaser* Crystallographic Software. *J. Appl. Crystallogr.* **2007**, *40* (4), 658–674. https://doi.org/10.1107/S0021889807021206.

(7) Emsley, P.; Cowtan, K. Coot: Model-Building Tools for Molecular Graphics. *Acta Crystallogr. D Biol. Crystallogr.* **2004**, *60* (Pt 12 Pt 1), 2126–2132. https://doi.org/10.1107/S0907444904019158.

(8) Murshudov, G. N.; Skubák, P.; Lebedev, A. A.; Pannu, N. S.; Steiner, R. A.; Nicholls, R. A.; Winn, M. D.; Long, F.; Vagin, A. A. REFMAC5 for the Refinement of Macromolecular Crystal Structures. *Acta Crystallogr. D Biol. Crystallogr.* **2011**, *67* (Pt 4), 355–367. https://doi.org/10.1107/S0907444911001314.

(9) Long, F.; Nicholls, R. A.; Emsley, P.; Gražulis, S.; Merkys, A.; Vaitkus, A.; Murshudov, G. N. AceDRG: A Stereochemical Description Generator for Ligands. *Acta Crystallogr. D Struct. Biol.* **2017**, *73* (Pt 2), 112–122. https://doi.org/10.1107/S2059798317000067.

(10) Davis, I. W.; Leaver-Fay, A.; Chen, V. B.; Block, J. N.; Kapral, G. J.; Wang, X.; Murray, L. W.; Arendall, W. B.; Snoeyink, J.; Richardson, J. S.; Richardson, D. C. MolProbity: All-Atom Contacts and Structure Validation for Proteins and Nucleic Acids. *Nucleic Acids Res.* **2007**, *35*, W375-383. https://doi.org/10.1093/nar/gkm216.
